# Obstetric Outcomes in Women on Lithium: A Systematic Review and Meta-Analysis

**DOI:** 10.3390/jcm13164872

**Published:** 2024-08-18

**Authors:** Tommaso Callovini, Silvia Montanari, Francesca Bardi, Sara Barbonetti, Sara Rossi, Romina Caso, Giuseppe Mandracchia, Stella Margoni, Andrea Brugnami, Marco Paolini, Giovanni Manfredi, Luca Lo Giudice, Daniele Segatori, Andrea Zanzarri, Luca Onori, Claudia Calderoni, Elisabetta Benini, Giuseppe Marano, Marco Massetti, Federica Fiaschè, Federica Di Segni, Delfina Janiri, Alessio Simonetti, Lorenzo Moccia, Flavia Grisoni, Sara Ruggiero, Giovanni Bartolucci, Marco Biscosi, Ottavia Marianna Ferrara, Evelina Bernardi, Leonardo Monacelli, Alessandro Michele Giannico, Domenico De Berardis, Giulia Battisti, Michele Ciliberto, Caterina Brisi, Francesco Maria Lisci, Antonio Maria D’Onofrio, Antonio Restaino, Luca Di Benedetto, Maria Benedetta Anesini, Gianluca Boggio, Elettra Specogna, Arianna Crupi, Emanuela De Chiara, Emanuele Caroppo, Valentina Ieritano, Laura Monti, Daniela Pia Rosaria Chieffo, Lucio Rinaldi, Giovanni Camardese, Ilaria Cuomo, Roberto Brugnoli, Georgios D. Kotzalidis, Gabriele Sani, Marianna Mazza

**Affiliations:** 1 Department of Psychiatry, Fondazione Policlinico Universitario Agostino Gemelli IRCCS, L.Go Agostino Gemelli 8, 00168 Rome, Italy; t.callovini@gmail.com (T.C.); silvia.montanari.rm@gmail.com (S.M.); francesca.bardi97@gmail.com (F.B.); sara.barbonetti@gmail.com (S.B.); sara.rossi1349@gmail.com (S.R.); casoromina@gmail.com (R.C.); peppemandracchia@gmail.com (G.M.); stella.margoni98@gmail.com (S.M.); andreabrugnami@gmail.com (A.B.); logiudiceluca@outlook.it (L.L.G.); dottorsegatori@gmail.com (D.S.); andreazanzarri@gmail.com (A.Z.); luca.onori@yahoo.it (L.O.); elisabetta.bnn@gmail.com (E.B.); marcomassetti1@gmail.com (M.M.); delfina.janiri@unicatt.it (D.J.); alessio.simonetti@policlinicogemelli.it (A.S.); lorenzomoccia27@gmail.com (L.M.); dr.flaviagrisoni@gmail.com (F.G.); sara.ruggi95@gmail.com (S.R.); giovannibartolucci94@hotmail.it (G.B.); mb140594@gmail.com (M.B.); ottaviaferrara@icloud.com (O.M.F.); evelinabernardi@gmail.com (E.B.); monacelli.leonardo@gmail.com (L.M.); alessandromichele.giannico@gmail.com (A.M.G.); giulia.battisti7@gmail.com (G.B.); micheleciliberto@libero.it (M.C.); caterinabrisi@libero.it (C.B.); fmlisci@gmail.com (F.M.L.); antoniomdonofrio@gmail.com (A.M.D.); restainoantonio11@gmail.com (A.R.); luca.dibenedetto91@gmail.com (L.D.B.); mbenedetta@hotmail.it (M.B.A.); gianlu88us22@hotmail.it (G.B.); elettraspecogna@libero.it (E.S.); ariannacrupi1@gmail.com (A.C.); dechiaraemanuela@gmail.com (E.D.C.); valentinaieritano.nutrizione@gmail.com (V.I.); lucio.rinaldi@policlinicogemelli.it (L.R.); giovanni.camardese@policlinicogemelli.it (G.C.); roberto.brugnoli@uniroma1.it (R.B.); giorgio.kotzalidis@gmail.com (G.D.K.); gabriele.sani@unicatt.it (G.S.); 2 Department of Neuroscience, Section of Psychiatry, Università Cattolica del Sacro Cuore, Largo Francesco Vito 1, 00168 Rome, Italy; 3 Department of Psychiatry, Università Vita-Salute San Raffaele, 20132 Milan, Italy; paolini.marco@hsr.it; 4 UOC Psichiatria, Sant’Andrea University Hospital, Università La Sapienza of Rome, Via di Grottarossa 1035-1039, 00189 Rome, Italy; giovanniwalter.manfredi@gmail.com; 5 ASL RM1, Presidio Ospedaliero San Filippo Neri, Servizio Psichiatrico di Diagnosi e Cura, Via Giovanni Martinotti, 20, 00135 Rome, Italy; federica.fiasche@aslroma1.it; 6 ASL Roma 2, Dipartimento di Salute Mentale, Servizio Per Le Dipendenze Patologiche Distretto 7, Via dei Sestili 7, 00174 Rome, Italy; federica.disegni@aslroma2.it; 7 Department of Mental Health, ASL 4 Teramo, 64100 Teramo, Italy; domenico.deberardis@aslteramo.it; 8 Department of Mental Health, Local Health Authority Roma 2, 00159 Rome, Italy; emanuele.caroppo@aslroma2.it; 9 Clinical Psychology Unit, Fondazione Policlinico Universitario Agostino Gemelli IRCCS, 00168 Rome, Italy; laura.monti@policlinicogemelli.it (L.M.); danielapiarosaria.chieffo@unicatt.it (D.P.R.C.); 10 Department Women Children and Public Health, Catholic University of Sacred Heart, 00168 Rome, Italy; 11 ASL RM1, UOC SM Distretto XIII ASL Roma 1, CSM Via Boccea 271271, 00168 Rome, Italy; ilaria.cuomo@aslroma1.it

**Keywords:** lithium salts, pregnancy, foetal outcomes, congenital malformations, Ebstein’s anomaly, small for gestational age, large for gestational age

## Abstract

**Background/Objectives**: Lithium taken during pregnancy was linked in the past with increased risk for foetal/newborn malformations, but clinicians believe that it is worse for newborn children not to treat the mothers’ underlying psychiatric illness. We set to review the available evidence of adverse foetal outcomes in women who received lithium treatment for some time during their pregnancy. **Methods**: We searched four databases and a register to seek papers reporting neonatal outcomes of women who took lithium during their pregnancy by using the appropriate terms. We adopted the PRISMA statement and used Delphi rounds among all the authors to assess eligibility and the Cochrane Risk-of-Bias tool to evaluate the RoB of the included studies. **Results**: We found 28 eligible studies, 10 of which met the criteria for inclusion in the meta-analysis. The studies regarded 1402 newborn babies and 2595 women exposed to lithium. Overall, the systematic review found slightly increased adverse pregnancy outcomes for women taking lithium for both the first trimester only and any time during pregnancy, while the meta-analysis found increased odds for cardiac or other malformations, preterm birth, and a large size for gestational age with lithium at any time during pregnancy. **Conclusions**: Women with BD planning a pregnancy should consider discontinuing lithium when euthymic; lithium use during the first trimester and at any time during pregnancy increases the odds for some adverse pregnancy outcomes. Once the pregnancy has started, there is no reason for discontinuing lithium; close foetal monitoring and regular blood lithium levels may obviate some disadvantages of lithium administration during pregnancy.

## 1. Introduction

Lithium salts were introduced in clinical psychiatry in 1949 by the Australian psychiatrist John Cade [1]. Its introduction in Europe was due to Mogens Schou’s work in Denmark five years later; Schou first conducted a randomised, placebo-controlled study in patients with mania and showed the usefulness of this molecule [2]. Although Samuel Gershon and Arthur Yuwiler had already advocated the use of lithium salts to treat mania in the early sixties in the US [3], the drug was not introduced in that country earlier than 1970 [4].

The first observations of possible detrimental effects of the use of lithium in pregnant women date back to the mid-1970s [5,6,7]. The focus was on malformations of the foetus and the newborn, especially cardiovascular [7]. It was Schou himself who coined the term “lithium babies” [5] and later wondered what the outcome of those babies born apparently healthy from mothers taking lithium was [8]. Although the risk for foetal malformations is considered to be generally low with continuing lithium use during pregnancy [9] and less than the previously reported risk [10], the question of whether malformations may be attributed to the use of lithium or to the underlying disorder is still unresolved. In fact, bipolar disorder (BD) per se constitutes a risk factor in pregnancy for future altered foetal growth and birth defects like microcephaly, adverse central nervous system (CNS) outcomes, small size for gestational age, and other congenital anomalies [11]. Hence, the risk of taking lithium during pregnancy must be weighed against the perils of untreated BD (or treating it with other mood-stabilising drugs). When analysing the literature, lithium was found to bear a reduced teratogenic risk compared to valproate and carbamazepine [12], while inconsistent results have been reported for lamotrigine [13]; generally, the risks of lamotrigine and oxcarbazepine are considered to be low and similar, but these data regard pregnancies of women with epilepsy [14,15] and they differ depending on mood stabiliser dosages.

The general attitude of the lay public (and some physicians) towards the use of lithium during pregnancy is ambiguous. This attitude does not involve only lithium, but also any medication, with self-medicating and lack of knowledge of drugs being quite frequent [16]. In the past, as soon as pregnancy was ascertained or planned, lithium was to discontinue immediately, but fortunately, the current tendency is to maintain treatment and control lithium blood levels frequently [17]. However, humans base their decision making on early accumulated data that start being built-up before sufficient evidence is accumulated [18], so the larger effects of old studies influence the conclusions of updated meta-analyses [19]. Publication bias apparently inflates the results of both pharmacotherapies and psychotherapies [20,21]. This results in a negative picture created by early studies on the use of lithium during pregnancy that is hard to overcome. Therefore, if we need to provide physicians treating pregnant women on lithium with adequate recommendations, we need to rely on a meta-analysis that addresses the question of whether lithium during pregnancy is linked to increased risk of congenital malformations in the baby.

In this review, we attempted to assess the risk of taking lithium in pregnancy for the foetus and newborn by pooling all the available data and meta-analysing data of specific foetal/newborn outcomes.

## 2. Materials and Methods

This systematic review and meta-analysis was conducted following the Preferred Reporting Items for Systematic Reviews and Meta-Analyses (PRISMA) 2020 Statement [22].

### 2.1. Eligibility Criteria

We included observational studies testing the association between lithium exposure in pregnant women and clinical outcomes of the foetus or newborn. We excluded studies (i) not providing information on lithium exposure, (ii) providing insufficient data on correlates to be included in the meta-analysis, and (iii) studies of data from the same sample to avoid duplicates. We also excluded studies with incomplete data or those not subjected to peer-review, such as conference abstracts and grey literature. We included studies without a control group for the qualitative synthesis of the evidence, whereas the quantitative extraction was performed only on those studies that used a control group, allowing an effect size computation.

### 2.2. Search Strategy and Selection of Studies

We searched the PubMed, Cochrane, CINAHL, and PsycINFO/PsycARTICLES databases for articles indexed up to 8 April 2024, without language restrictions, using the following search strategy: (Obstetric outcomes in women with Bipolar Disorder treated with lithium) OR ((outcome* AND (birth OR delivery OR pregnan* OR obstetric* OR fetal OR foetal)) AND “bipolar disorder” AND lithium) OR (pregnancy outcome AND lithium). We also searched the ClinicalTrials.gov register using the search: Condition/disease: pregnancy; Other terms: _; Intervention/treatment: Lithium. A supplemental, post hoc, non-systematic search on Google Scholar was conducted to determine if additional studies were retrievable. All the authors independently completed the screening based on all the retrieved articles. The authors met in Delphi rounds to establish eligibility according to the inclusion criteria. Disagreements concerning suitability for inclusion were resolved by discussion and consensus involving all the authors. No more than two rounds were required to establish consensus. The studies thus retrieved were tabulated and are summarised in Table 1. They were further processed for eligibility for the meta-analysis, as described above. Papers were included if they had data on lithium administration during pregnancy and neonatal outcomes. We excluded studies when they were reviews or meta-analyses; however, the reference lists of these studies were searched for possible eligible studies that eluded our search. Excluded were also studies not reporting data on pregnancy; editorials, hypotheses, viewpoints, letters to the editor, or comments of other articles that contained no original data, collectively labelled as opinions; case reports or case series, labelled as case; studies not reporting separate data on lithium (labelled as no lithium or lumping when they reported data on lithium and other treatments without distinction); studies not reporting birth outcomes (labelled as no outcome) when their target was unfocused as regards our aims (labelled as unfocused); when they were unrelated to the subject matter and resulted in the search without any reason (labelled as unrelated); when they were protocols without even preliminary data (labelled as protocol); and when they were not conducted on humans (labelled as animal) or they regarded only tissues or cellular systems (labelled in vitro).

We assessed the risk of bias (RoB) of the included studies with the appropriate Cochrane RoB 2.0 tool (IBM SPSS Statistics for Windows, New York, USA.), Risk Of Bias In Non-randomized Studies—of Exposure (ROBINS-E) [23]. We performed an evaluation of the RoB for each included study. The results are shown in the Online Supplement, Supplementary Figure S8.

### 2.3. Data Extraction

We used an Excel (Microsoft Office 2024, New York, USA) data extraction template including key items for all the eligible studies: year of publication, country, setting, inclusion criteria, sample size, mean age, sociodemographic and clinical characteristic of the sample, and main findings. Four authors (TC, GM, SB, FB) independently extracted data for a blind check of accuracy. Authors of studies with unclear or partial data were contacted by email for additional information to reduce the risk of selective reporting bias.

### 2.4. Data Analysis

Different meta-analyses were carried out on each correlate for which data were available from at least three different studies. When two or more studies had partial or entire sample overlap for a specific correlate, the study with the largest amount of information was included in the analysis (sample size and data quality were considered). Meta-analyses of the association between lithium exposure and relevant correlates among individuals with affective disorders were based on an odds ratio (OR) with a 95% confidence interval (CI). Pooled estimates were obtained by weighting each study according to a random effects model for meta-analysis. Heterogeneity between the studies was evaluated using standard cut-offs for the I^2^ statistic, with values of 25%, 50%, or 75% defining different levels of inconsistency (low, moderate, or high). Analyses were performed in the jamovi project (2022) using jamovi (Version 2.3, https://www.jamovi.org) computer software, retrieved from https://www.jamovi.org/features.html (accessed on 8 April 2024).

## 3. Results

**Figure 1:**
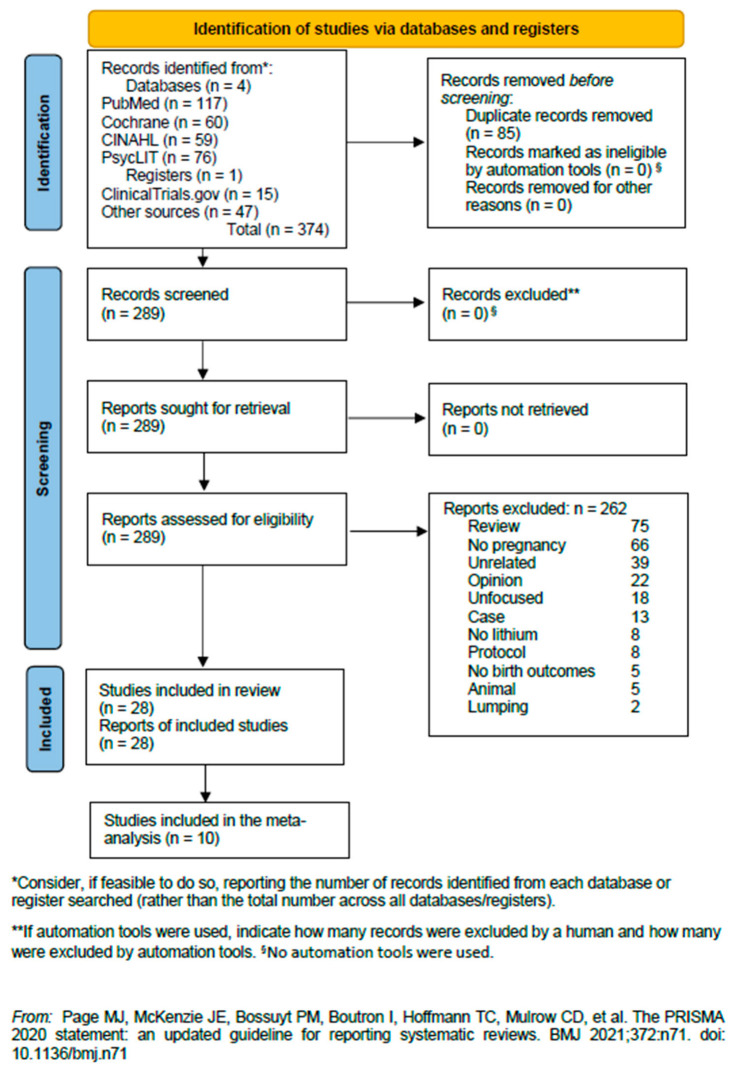
PRISMA 2020 flow diagram of our selection process, with reasons for exclusion [22]. For more information, visit: http://www.prisma-statement.org/.

**Figure 2:**
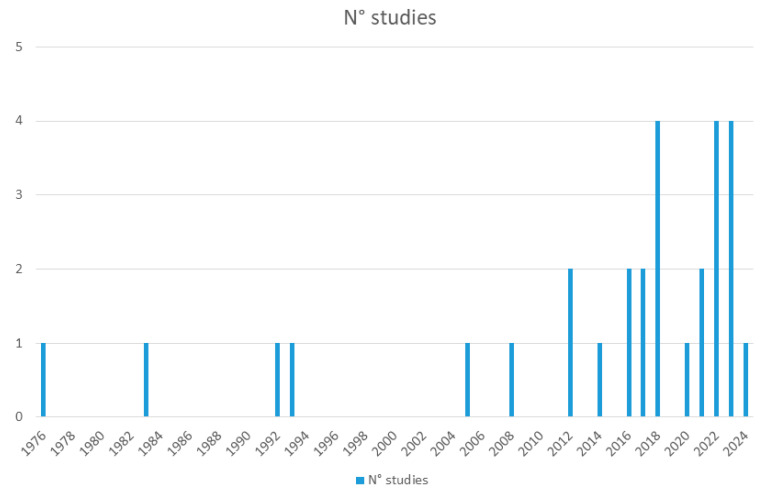
Distribution of eligible studies over time.

Our search was conducted in all the databases on 28 March, 2024. In PubMed, it obtained 117 results; in Cochrane Central Register of Controlled Trials/Cochrane Database of Systematic Reviews/Cochrane Methodology Register/Cochrane Clinical Answers, 60 records; in Cinahl, 59 results; in PsycINFO/PsycARTICLES/Psychology and Behavioral Sciences Collection, 76 records; and in ClinicalTrials.gov, using an alternative overinclusive strategy, 15 records. From other sources, on the same day, we obtained another 45 records. The articles resulting from the searches amounted to a grand total of 372. There were 85 duplicates among these databases; therefore, there remained 287 articles. Of these, we included in our systematic review 28 studies and excluded 75 that were reviews or meta-analyses, 66 that did not report data on pregnancy, 39 that were unrelated to the subject matter, 22 that were opinion papers, like editorials and letters to the editor with no data, 18 that were unfocused as concerns our target, 13 that were case reports or case series, 8 papers that did not report data on lithium, 8 that were protocols with no data, 5 that did not report birth outcomes, 5 that were animal studies, and 2 that were lumped data of lithium with those of other treatments. The included articles and their summaries are shown in Table 1. The inclusion process is shown in Figure 1 and Supplementary Table S1. Of the included studies, 10 were used in our meta-analysis [24,25,26,27,28,29,30,31,32,33]. The results of the search spanned from 1971 to 26 March 2024, with the eligible studies ranging from September 1976 to 18 January 2024. The per-year distribution is quite heterogeneous (Figure 2).

### 3.1. Methodologies of Included Studies

The included studies used different methodologies in their patient inclusion. Some were based on national registries, others used hospital or clinics, while others used both, and some were the product of international collaboration. The first studies, published during the late 1970s and early 1980s, were from Scandinavian countries and used a Scandinavian register [8] and several Swedish registries [24]. A further Swedish study by one of the latter authors [34] used the Swedish Medical Birth Register. In the 1990s, international collaborations were born, using teratogen information services like Motherisk (Toronto, ON, Canada), California Teratogen Information Service (CTIS) (San Diego, CA, USA), Philadelphia Pregnancy Healthline (Philadelphia, PA, USA), and Foetal Risk Assessment from Maternal Exposure (FRAME) (London, ON, Canada) [35] or the International Register of Lithium Babies [25]. The 21st century saw collaborations not limited to North America but extending to Europe and the Middle East, combining, for example, the pioneering Motherisk Program (Toronto, ON, Canada) with the Israeli Teratogen Information Service (Jerusalem, Israel) and the Drug Safety Research Unit (Southampton, England, UK) [36]. However, individual centres and registers continued to publish on this subject in the Netherlands (Perinatal Center of the Leiden University Medical Center) [37], Sweden (Swedish prescribed drug register, Swedish medical birth register, and Swedish national patient register) [38], Israel (Israeli Teratology Information Service, Jerusalem) [26], Italy (Psychiatric Unit of the Department of Neurosciences, University of Turin [39] and Teratology Information Service at Agostino Gemelli Hospital, Rome, Italy) [40], and the UK (Health Improvement Network and Clinical Practice Research Datalink) [41]. Networks developed in the second decade of the 21st century, like the European Surveillance of Congenital Anomalies (EUROCAT) network of registries of congenital anomalies, including 21 European countries; of these, 15 centres of 12 countries agreed to participate in a lithium study [42]. Other studies involved the Medicaid US database [27]; the Affective Disorder Outpatient Clinic, Psychiatry Clinic Southwest, Stockholm, Sweden [28]; and the Childbirth and Mental Illness service, King Edward Memorial Hospital, Perth, Western Australia [43], while a patient-level meta-analysis used population-based cohorts in Denmark, Sweden, and Ontario, Canada, as well as clinical psychiatric cohorts in the Netherlands, UK, and US [29]. The most recent studies witnessed an increasing presence of the Netherlands (Dutch Bipolar Cohort, collaborative study between UCLA, Los Angeles, California, US and eight Dutch centres [12] of the Erasmus Medical Center Rotterdam, Leiden University Medical Center [44], with the addition of Onze Lieve Vrouwe Gasthuis Amsterdam [31,33,45,46]). The most recent Swedish studies used either a large number of national registries (Swedish Medical Birth Register, Swedish Patient Register, Swedish Prescribed Drug Register, Swedish Education Register, Swedish Total Population Register, [30] or two hospitals in Stockholm (Karolinska University Hospital and Sachs’ Children’s and Adolescents’ Hospital [47])). Two Spanish studies used hospital records (Perinatal Mental Health Unit, Hospital Clínic, Barcelona, Catalunya) [32] and a local cohort in Ourense, Galicia [48]. Finally, a Belgian study used neonatal intensive care units and medium-care units where lithium babies were hospitalised (University Hospitals, Leuven, Belgium) [49]. The summary of the included studies is shown in Table 1.

All the included studies amounted to a figure of 1402 babies exposed to lithium during their mothers’ pregnancies and 2595 pregnant women who were exposed to lithium at any time. Of these, 35 regarded mother–baby dyads.

**Table 1:** Summary of studies investigating pregnancy and birth outcomes in women who received lithium during pregnancy in chronological order (older to newer).

Study	Population	Design/Study Type	Li^+^-Related Birth/Pregnancy Outcomes	Conclusions
Schou, 1976 [8]	67 (26 ♀, 34 ♂) babies treated with Li^+^; 57 HCs (siblings 25 ♀, 32 ♂)	Questionnaire with the mother’s name, birth data, and address, and the Li^+^ child’s birth data were filled in. The informant was asked to submit information about name, birth data, sex, physical development, and mental development of all the woman’s children.	Development anomalous 10 Li^+^ children, 6 HCs. Among the Li^+^ children with developmental anomalies, three had been exposed to Li^+^ during the first trimester only, and seven had been exposed during the entire foetal period. The difference between these frequencies is not statistically significant.	The data obtained do not reveal any ↑ frequency of physical or mental anomalies among the Li^+^ children.
Källén & Tandberg 1983 [24]	350 infants whose mothers were treated as inpatients for manic-depressive disease	Cohort study. Several registers were used: DRPW, MBR, RCM	The mothers were divided into five groups: (1) no information on psychiatric illness before pregnancy indicated in chart, (2) diagnosis of psychiatric illness given in the chart but no mention of drug use, (3) psychotropic drug given (disease, of course, present) but no Li^+^ given, (4) only Li^+^ given, and (5) Li^+^ and other psychotropic drug given.	There is no statistically significant difference between delivery outcome in women on Li^+^ and in ♀ on other psychotropic drugs.
Jacobson et al., 1992 [35]	148 ♀ mean age 30 yrs, using Li^+^ in the first trimester of pregnancy; 148 HC matched for age	Multicentre study	The rates of major congenital malformations did not differ between the Li^+^ (2.8%) and control (2.4%) groups.	The pregnancy outcome did not differ between patients and controls with respect to the total number of livebirths, frequency of major anomalies, spontaneous or therapeutic abortions, ectopic pregnancy, and prematurity.
Troyer et al., 1993 [25]	60 ♀ treated with lithium; 290 ♀ not exposed to lithium	Retrospective cohort study	The RR of premature delivery for ♀ taking lithium during pregnancy was × 2.54 that of ♀ with or without manic-depressive illness who were not receiving Li^+^ during pregnancy.	Lithium exposure during pregnancy was linked to an increased risk for pre-term birth.
McKenna et al., 2005 [36]	105 ♀ exposed to atypical APs and 105 ♀ to non-teratogenic agents in the 1st trimester matched for gestational age. Ages not provided	Motherisk Program in Toronto Ontario, Canada; Israeli Teratogen Information Service in Jerusalem, Israel; and Drug Safety Research Unit in Southampton, England, UK (3 sites). Observational study of a cohort	Li^+^ was taken by six ♀ during pregnancy at some point; 5/6 discontinued it. No major malformation reported.	Although the study focused on APs, scanty data on Li^+^ were reported that showed no malformations with Li^+^.
Reis & Källén, 2008 [34]	2908 ♀ treated with APs (79 with Li^+^)	This cohort study is based on data from the Swedish Medical Birth Register. Maternal drug use in early pregnancy is recorded from interviews performed by the midwife at the first antenatal care visit, usually before the end of the first trimester.	A total of 79 ♀ were treated with Li^+^. Among them, eight had a diagnosis of a congenital malformation. One infant had Down syndrome, one had an unspecified skin malformation, and two had an unstable hip. Four had cardiac defects: one had a combined atrium septum defect and tricuspid and mitral malformations, one had mitral insufficiency and hypospadias, one had a ventricular septum defect, and one had patent ductus arteriosus in a term baby (born after 41 completed weeks). Even though the rate of cardiac defects was high (5.1%), it had a wide CI (1.4–12.5%), and the defects were relatively mild.	↑ risk for congenital malformations (mainly cardiovascular defects), nearly double the risk of for gestational diabetes, and a 40% ↑ risk for caesarean delivery was noted. No certain drug specificity was found. Because there seems to be little drug specificity, it is possible that underlying pathology or unidentified confounding comorbidities explain the excess risk.
van der Lugt et al., 2012 [37]	15 children who were exposed to Li^+^ in utero were investigated at 3–15 years of age	Observational retrospective cohort study. Neurological development was tested using the Hempel or Touwen examination. Cognitive development was assessed with the Bayley Scales of Infant Development III, Wechsler Preschool and Primary Scale of Intelligence, or the Wechsler Intelligence Scale for Children. Parents completed the Child Behavior Checklist to assess behavioural development and a standard questionnaire about general development of the child since birth.	One child had signs of a minor neurological dysfunction but without further clinical implications. The results of the cognitive tests were within normal limits, although most children had lower scores on the performance IQ subtest. Growth, behaviour, and general development were within the normal ranges.	Continuing Li^+^ therapy during pregnancy did not cause adverse effects on growth, neurological, cognitive, and behavioural development of exposed children.
Bodén et al., 2012 [38]	332,137 ♀ (of whom 320 were in treatment for BD, 554 with BD untreated); both groups were compared with all other ♀ giving birth (331,263). 76 ♀ exposed to Li^+^	Cohort study using data from national health registers	The risks of preterm birth in both treated and untreated were ↑ by 50%. Of the untreated ♀, 3.9% (*n* = 542) had a microcephalic infant compared with 2.3% (324 844) of the ♀ without BD (1.68, 1.07 to 2.62). The corresponding values for the treated ♀ were 3.3% (*n* = 311) (1.26, 0.67 to 2.37). Similar trends were observed for risks of infants being small for gestational age according to infants’ weight and length. Among the infants of untreated ♀, 4.3% (*n* = 24) had neonatal hypoglycaemia compared with 2.5% (*n* = 8302) among the infants of ♀ without BD (1.51, 1.04 to 2.43), and 3.4% (*n* = 11) of the treated ♀ (1.18, 0.64 to 2.16). The analyses of variation in the outcomes did not support any significant differences between treated and untreated ♀.	BD in ♀ during pregnancy, whether treated or not, was associated with ↑ risks of adverse pregnancy outcomes.
Diav-Citrin et al., 2014 [26]	183 ♀ Li^+^-exposed pregnancies compared with 72 disease-matched and 748 non-Li^+^-exposed pregnancies	Prospective, comparative observational study	There were significantly more miscarriages (adjusted odds ratio = 1.94, 95%CI = 1.08–3.48) and elective terminations of pregnancy (17/183 [9.3%] compared with 15/748 [2.0%]) in the Li^+^-exposed group compared with the non-teratogenic exposure group. Major congenital anomalies not significantly different. Cardiovascular anomalies occurred more frequently in the Li^+^ group exposed during the first trimester when compared with the non-teratogenic exposure group. Non-cardiovascular anomalies were not significantly different between the groups. Preterm deliveries ↑ in the Li^+^ group compared with non-teratogenic exposure.	Li^+^ treatment in pregnancy is associated with a higher rate of cardiovascular anomalies. ♀ who are treated with Li^+^ during organogenesis should undergo foetal echocardiography and level-2 ultrasound.
Rosso et al., 2016 [39]	17 Li^+^-treated ♀ with severe BD-I who wished to become pregnant	Lo. Treatment with flexible doses of Li + combined with supportive psychotherapy throughout the pregnancy and the postpartum period	Li^+^ was generally well tolerated: three ♀ became overweight and two ♀ reported mild polyuria and polydipsia (already reported with Li^+^ treatment before pregnancy). No congenital abnormalities occurred. Three babies with mild hypotonia spontaneously recovered. Recurrences of any polarity during pregnancy occurred in 11.1% of ♀. Post-delivery rate of psychiatric disorders 29.4%; in other studies of unmedicated BP ♀, postpartum recurrence rate was generally much ↑.	The results support the prophylaxis efficacy of Li^+^ throughout pregnancy in Li^+^-responder BD-I ♀ who have ↑ risk of severe peripartum recurrences.
Petersen et al., 2016 [41]	28 ♀ exposed to Li^+^ during pregnancy; 57 ♀ who discontinued Li^+^ during pregnancy; 212,531 ♀ not prescribed APs	Retrospective cohort study. Based on UK electronic primary care health records from the Clinical Practice Research Datalink database and the Health Improvement Network primary care database. Among the many objectives, one was to identify risk factors predictive of discontinuation of and restarting Li^+^ (from multiple manufacturers), anticonvulsant mood stabilizers, and AP medication	No birth/pregnancy outcomes associated with Li^+^	It was impossible to investigate Li^+^ or anticonvulsant use-associated risk specifically for psychoses owing to the small numbers of ♀ in these groups.
Boyle et al., 2017 [42]	173 Ebstein’s anomaly babies/foetuses; 51,024 non-cardiac controls; 26,170 cardiac controls	Descriptive epidemiological analysis using population-based data. The aim was to describe the epidemiology of Ebstein’s anomaly in Europe and investigate its associations with maternal health and medication exposure during pregnancy. Data were taken from 15 European Surveillance of Congenital Anomalies (EUROCAT) Congenital Anomaly Registries in 12 European countries, with a population of 5.6 million births during 1982–2011.	No case of Ebstein’s anomaly was associated with Li^+^ assumption during pregnancy.	Li^+^ not associated with Ebstein’s cardiac anomaly in those 13 patients who took Li^+^. Figures provided are unlikely.
Patorno et al., 2017 [27]	1,325,563 ♀, 663 infants exposed to Li^+^ first trimester, 1945 exposed to lamotrigine first trimester, 1,322,955 unexposed infants	Cohort study. This study examined the risk of cardiac malformations among infants exposed to Li^+^ during the first trimester as compared with unexposed infants and, in secondary analyses, with infants exposed to another commonly used mood stabilizer, lamotrigine.	Cardiac malformations were present in 16 of the 663 infants exposed to Li^+^ (2.41%) and 15,251 of the 1,322,955 unexposed infants (1.15%). The adjusted risk ratio for cardiac malformations among infants exposed to Li^+^ as compared with unexposed infants was 1.65 (95% CI: 1.02 to 2.68). The risk ratio was 1.11 (95%CI: 0.46 to 2.64) for a daily dose of 600 mg or less, 1.60 (95%CI: 0.67 to 3.80) for 601 to 900 mg, and 3.22 (95%CI: 1.47 to 7.02) for more than 900 mg. The prevalence of right ventricular outflow tract obstruction defects was 0.60% among Li^+^-exposed infants vs. 0.18% among unexposed infants (adjusted risk ratio, 2.66; 95%CI, 1.00 to 7.06).	Maternal use of Li^+^ during the first trimester was associated with ↑ risk of cardiac malformations, including Ebstein’s anomaly; the magnitude of this effect was smaller than had been previously postulated.
Forsberg et al., 2018 [28]	20 ♀ with MD exposed to Li^+^ treatment during pregnancy; eight ♀ with MD (excluding Li^+^ treatment exposure); 11 ♀ controls (no MD or Li^+^ treatment exposure)	Retrospective cohort study (♀ gave birth between 2006 and 2010)	The children’s full-scale IQ, performance IQ, and verbal IQ results did not differ significantly between the groups.	No significant association between mothers’ prenatal exposure to Li^+^ or mood disorders and their offspring’s IQ was found.
Frayne et al., 2018 [43]	19 ♀ exposed to Li^+^ at any time during pregnancy; 14 ♀ exposed to Li^+^ at conception but ceased Li^+^ at confirmation of pregnancy	Retrospective cohort study (♀ gave birth between December 2007 and January 2015 at a specialist antenatal clinic in Western Australia)	Of the babies exposed to Li^+^ during pregnancy and at delivery (*n* = 19), eight (42%) were admitted to the SCN post-delivery, of which six (31.6%) were admitted to a level-3 or neonatal intensive care unit.	Exposure to Li^+^ during pregnancy was associated with ↑ rates of foetal ultrasound abnormalities, such as abdominal circumference >90th %.
Munk-Olsen et al., 2018 [29]	22,124 eligible pregnancies, of which 727 pregnancies were eligible for inclusion in the Li^+^-exposed group	In this meta-analysis, primary data from pregnant ♀ and their children from six international community-based (Denmark, Sweden, and Ontario, Canada) and clinic-based (the Netherlands, UK, and USA) cohorts were analysed. This study aimed to investigate the association between in-utero Li^+^ exposure and the risk of pregnancy complications, delivery outcomes, neonatal morbidity, and congenital malformations. aORs and 95% CIs were calculated through logistic regression models, and site-specific prevalence rates and ORs were pooled using random-effects meta-analytical models.	Li^+^ exposure was not associated with any of the predefined pregnancy complications or delivery outcomes. ↑ risk for neonatal readmission within 28 days of birth was seen in the Li^+^-exposed group compared with the reference group (pooled prevalence 27·5% [95% CI: 15·8 to 39·1] vs 14·3% [10·4 to 18·2]; pooled aOR 1·62, 95% CI 1·12 to 2·33). Li^+^ exposure during the first trimester was associated with ↑ risk of major malformations (pooled prevalence 7.4% [95% CI: 4.0 to 10.7] vs. 4.3% [3.7 to 4.8]; pooled aOR 1.71, 95%CI: 1.07 to 2.72) but for major cardiac malformations, the difference was n.s. (2.1% [0.5 to 3.7] vs. 1.6% [1.0 to 2.1]; pooled aOR 1.54, 95% CI 0.64 to 3.70).	Treatment decisions for pregnant ♀ with mood disorders must weigh the potential for ↑ risks of Li^+^ during pregnancy, particularly those associated with use of Li^+^ during the first trimester, against its effectiveness at ↓ relapse.
Neri et al., 2018 [40]	140 ♀ exposed to Li^+^ treatment during pregnancy (range 20–46 yrs old)	Retrospective survey and a selective review of the literature regarding the management of fertile patients under Li^+^ treatment for BD.The survey was conducted by the Teratology Information Service (TIS) at A. Gemelli University Hospital in Rome from May 2002 to December 2015.	Among all the pregnancies, the data collected by the TIS showed one premature birth, six early spontaneous abortions, one case of major cardiac malformation (hypoplastic left heart syndrome), four transient respiratory distress at birth, and two language delays.	No significant results emerged. The conclusion of the study is that peri-conception counselling is crucial for the outcome of pregnancy and for maternal health status during preconception, gestation, and breastfeeding.
Poels et al., 2020 [12]	77 ♀ with BD type 1 exposed to Li^+^ during pregnancy; 366 ♀ with BD type 1 not exposed to Li^+^	Retrospective observational cohort study. The aim was to evaluate the association between Li^+^ use during pregnancy and the occurrence of miscarriage.	Miscarriages occurred in 20.8% of pregnancies exposed to Li^+^ (*n* = 16 out of 77) compared to 10.9% of pregnancies not exposed to Li^+^ (*n* = 40 out of 366).	Li^+^ use during pregnancy may ↑ the risk of miscarriage among ♀ with BD-I disorder (adjusted OR = 2.94; 95% CI = 1.39–6.22).
Molenaar et al., 2021 [44]	78 ♀ with 100 pregnancies under Li^+^ treatment exposure (233 Li^+^ blood level measurements); 29 neonates with Li^+^ measurement in the 24 h postpartum	Retrospective observational cohort study. The first outcome was to evaluate ♀ Li^+^ blood level changes following delivery. The second outcome was to evaluate neonatal Li^+^ blood level and complications.	No association was found between time (wk before or after delivery) and the ratio of Li^+^ blood level/dose. No associations were found between neonatal Li^+^ blood levels at delivery and neonatal outcomes.	↓ the dosage or discontinuing Li^+^ prior delivery is not recommended. Stable dosing can prevent subtherapeutic Li^+^ serum levels, which is especially important in the postpartum period, when relapse risks are highest.
Hastie et al., 2021 [30]	434 ♀ exposed to Li^+^ treatment during pregnancy; 853,583 ♀ not exposed to Li^+^ treatment during pregnancy	Retrospective observational population-based cohort study. The aim was to examine the associations between Li^+^ use during pregnancy and the risk of adverse pregnancy and neonatal outcomes.	Maternal Li^+^ use during pregnancy was associated with ↑ risks of spontaneous preterm birth, birth of a large infant for gestational age, cardiac malformations, and neonatal hypoglycaemia. These associations remained significant in subgroup analyses among pregnant ♀ with diagnosed psychiatric illnesses and in analyses comparing ♀ who continued Li^+^ treatment during pregnancy versus those who discontinued treatment prior to pregnancy.	Li^+^ use during pregnancy is associated with ↑ risk of spontaneous preterm birth, infants large for gestational age, cardiac malformations, and potentially other adverse neonatal outcomes.
Sagué-Vilavella et al., 2022 [32]	53 ♀ with BD exposed to Li^+^ treatment during pregnancy; 47 ♀ with BD not exposed to Li^+^ during pregnancy	Retrospective observational cohort study. The aim was to compare obstetric outcomes in ♀ with BD with and without Li^+^ treatment during pregnancy.	No significant differences in obstetric complications, neonatal complications, or congenital anomalies were observed between the groups. Newborns of Li^+^-treated ♀ had ↓ Apgar scores at 1 min and 5 min compared to newborns of ♀ who did not receive Li^+^ during pregnancy.	Li^+^ treatment during pregnancy did not lead to worse obstetric outcomes in ♀ with BD, except for the impact on newborn Apgar scores.
Poels et al., 2022 [31]	99 children of ♀ with a diagnosis of BD, aged 6–14: 56 were exposed to Li^+^ in utero and 43 were not exposed	CS. Neuropsychological tests were administered, including the SON test and the NEPSY-II-NL assessment. Linear and negative binomial regression models were used to investigate the association between prenatal Li^+^ exposure and neuropsychological functioning. In secondary analyses, the association between Li^+^ blood level during pregnancy and neuropsychological functioning was assessed.	Li^+^ use during pregnancy was associated with the total number of mistakes made on the Auditory Attention task but was statistically n.s. after full adjustment for potential confounding factors. No association between prenatal Li^+^ exposure and IQ was found. Additionally, no relationship between Li^+^ blood level during pregnancy and neuropsychological functioning was found after adjustment for potential confounders. Task outcomes in both groups were ≈ to the general population.	There was no evidence for significantly altered neuropsychological functioning of Li^+^ -exposed children when compared to non-exposed children.
Torfs et al., 2022 [49]	10 mother–neonate pairs with intrauterine exposure to Li^+^	Lo. The primary aim of this retrospective study was to assess the early postnatal characteristics and short-term outcomes of neonates with in utero exposure to Li^+^.	The x^−^ GA was 37 (IQR, 36–39) wks. The neonatal plasma Li^+^ concentration at birth was 0.65 (IQR 0.56–0.83) mmol/L, with a median neonate/mother ratio of 1.02 (IQR 0.87–1.08). The median length of neonatal stay was 8.5 (IQR 8–12) days. Three cases in this study population displayed hypotonia, three had respiratory symptoms, and one had hypoglycaemia. Four neonates displayed a (mild) abnormal clinical neurological evaluation in the early postnatal phase. One neonate was diagnosed with NDI and the same neonate was diagnosed with a CDH.	Although causality could not be assessed due to the small sample size, these findings illustrate that indeed mild to moderate neonatal symptoms after in utero exposure to Li^+^ have to be anticipated. A postnatal care protocol was proposed to enhance the quality of care for future neonates and to guide parental counselling. Future prospective protocol evaluation is needed.
Poels et al., 2023 [45]	63 children of ♀ with a diagnosis of BP, aged 8–14: 30 with and 33 without intrauterine exposure to Li^+^	CS. Global brain volume outcomes and white matter integrity were assessed using structural MRI and DTI, respectively. To assess how the data compared to the general population, global brain volumes were compared to data from the Generation R study (*N* = 3243).	Linear regression analyses showed a n.s. negative association of intrauterine exposure to Li^+^ with subcortical grey matter volume (unstandardized β = −1.6, 95%CI: −3.1 to −0.1, *p*-value = 0.04, *p*-value after FDR correctio*n* = 0.48). The subcortical grey matter volume and cortical white matter volume were significantly reduced in Li^+^-exposed children compared to children from the Generation R cohort (unstandardized β = −1.4, 95%CI: −2.4 to −0.5, *p*-value = 0.004, *p*-value after FDR correctio*n* = 0.008 and unstandardized β = −14.3, 95%CI: −22.3 to −6.2, *p*-value <0.001, *p*-value after FDR correctio*n* =0.006, respectively).	Brain structure in Li^+^-exposed ≈ non-Li^+^-exposed children following correction for multiple testing
Álvarez-Silvares et al., 2023 [48]	79 low obstetric risk pregnant ♀	Lo. Prospective cohort study. The objective of this study was to correlate the placenta levels of 14 essential and non-essential elements with neonatal weight by conducting multivariable linear regressions using GLM and GAM.	Placental Co (*p* = 0.03) and Sr (*p* = 0.048) concentrations were associated with ↑ neonatal weight. Concentrations of Li^+^ (*p*= 0.027), Mo (*p* = 0.049), and Se (*p* = 0.02) in the placenta were associated with lower newborn weight.	The concentration of some metals in the placenta may affect foetal growth.
Schonewille et al., 2023 [33]	93 ♀ with BD who gave birth to 117 live-born neonates: 42 (36%) exposed (at any point throughout the pregnancy) and 75 (64%) non-exposed to Li^+^	Lo. Retrospective observational cohort study. Outcomes were obtained by medical chart review of ♀ and neonates and compared between neonates with and without Li^+^ exposure. The primary outcome was admission to a neonatal ward with monitoring, preterm birth, SGA, 5 min Apgar scores, neonatal asphyxia, and readmission ≤ 28 days.	There were n.s. differences in neonatal admission with monitoring (16.7 vs. 20.0%, *p* = 0.844). Additionally, preterm birth (7.1 vs. 5.3%), SGA (0.0 vs. 8.0%), 5 min Apgar scores (x^−^: 9.50 vs. 9.51), neonatal asphyxia (4.8 vs. 2.7%), and readmission (4.8 vs. 5.3%) were ≈. Overall, 18.8% of BD offspring was admitted. ♀ with BD had high rates of caesarean section (29.1%), gestational diabetes (12.8%), and hypertensive disorders of pregnancy (8.5%).	Exposure to Li^+^ was not associated with greater risk of neonatal admission to a ward with monitoring compared to non-exposure to Li^+.^ However, offspring of ♀ with BD were admitted regularly, and ♀ with BD have high obstetric risk that requires clinical and scientific attention.
Whaites Heinonen et al., 2023 [41]	25 infant–mother dyads: 7 infants with serum Li^+^ concentration at birth ≥0.6 meq/L (HEG); 18 with serum Li^+^ concentration at birth <0.6 meq/L (LEG)	Lo. Retrospective observational cohort study. This included breastfed infants to ♀ treated with Li^+^ during and after pregnancy. The first serum Li^+^ concentration counted for classifying infants as HEG or LEG. The first follow-up visits were at 2–4 wks of age.	x^−^ Li^+^ serum concentration at birth was 0.90 meq/L in HEG and 0.40 meq/L in LEG (*p* < 0.05). The difference was still significant at follow-up (0.20 meq/L vs. 0.06 meq/L, *p* < 0.05), despite reduction in maternal dose. Neonatal symptoms occurred in 85.7% HEG and 41.2% in LEG (*p* = 0.08; n.s.) at birth and 28.6% vs. 11.8% at follow-up (*p* = 0.55; n.s.). Furthermore, 28.6% of HEG infants were admitted to neonatal care vs. 5.9% LEG (*p* = 0.19). All the infants with symptoms at follow-up were either in HEG or exposed to additional psychotropic medication.	This study suggests that late intrauterine exposure to Li^+^ might add to the adverse effects of Li^+^-exposed breastfed infants.
Schrijver et al., 2024 [46]	81 ♀ and 101 pregnancies: 61 ♀ with one pregnancy, 20 ♀ with two pregnancies	Lo. Retrospective observational cohort study. This included ♀ with a history of BD or postpartum psychosis, used Li^+^ during pregnancy, and at least one Li^+^ serum level during pregnancy was available. Linear and logistic regression models used to investigate the association between weighted average Li^+^ level and pregnancy duration, birth weight percentiles, preterm birth, and LGA. Subsequent exploratory analyses investigated the role of TSH and T4 as mediators of associations.	Positive association between average Li^+^ level and risk of preterm birth (OR = 1.66 [95% CI: 1.05 to 2.61], *p* = 0.03 simple model, OR = 1.69 [95% CI: 1.06 to 2.68] *p* = 0.03 adjusted model [for mood episode during pregnancy and use of other psychotropics]). There were no significant associations were between the average Li^+^ level and birth weight percentiles and the risk of LGA birth. In exploratory analyses, TSH and T4 did not mediate the association between the average Li^+^ serum level and pregnancy duration.	Dose response relationship between maternal Li^+^ serum levels and pregnancy duration

Abbreviations: 95%CI, 95% confidence interval; Ag, silver; Al, aluminium; AP (s), antipsychotic (s); Bayley-III, Bayley Scales of Infant and Toddler Development, Third Edition; BD, bipolar disorder, -I, type I, -II, type II; Co, cobalt; CDH, congenital diaphragmatic hernia; cPIP, conditional posterior inclusion probability; CS, cross-sectional; DRPW, Discharge Registry for Inpatient Psychiatric Wards; DTI, diffusion tensor imaging; ENET, elastic net; ERS, element risk score; Fe, iron; FDR, false discovery rate; GA, gestational age; GAM, generalized additive models; GLM, generalized linear models; HC, healthy controls; HEG, high-exposure group; IQ, intelligence quotient; IQR, interquartile range; LEG, low-exposure group; LGA, large for gestational age; Li^+^, lithium ion; MBR, Medical Birth Registry; MD, mood disorders; mmol, millimole; Mo, molybdenum; MRI, magnetic resonance imaging; MS, mass spectrometry; meq, milliequivalents; n.s., not significant; NDI, nephrogenic diabetes insipidus; OR, odds ratio; RCM, Registry of Congenital Malformations; Sb, antimony; SD, ±, standard deviation; Se, selenium; SGA, small for gestational age; SON tests, Snijders–Oomen Nonverbal Intelligence Test; Sr, strontium; wk (s), week (s); x^−^, mean; yr (s), year (s); Zn, zinc; ×, for, per; ≈, about equal, not different; ♀, females; ♂, males; ↓, decreased, reduced, lower; ↑, increased, higher; →, induced, followed.

### 3.2. Studies Included in the Meta-Analysis

The outcome of the studies included in our meta-analysis were, after lithium exposure at any time, foetal heart anomalies in six studies [24,26,27,29,30,32], any foetal congenital anomaly in seven [24,26,28,29,30,32,33], preterm birth in eight [25,26,28,29,30,31,32,33], child small for gestational age in five [28,29,30,32,33], and child large for gestational age in three [30,32,33]; and in lithium in first trimester, foetal heart anomalies in five studies [24,26,27,29,32] and any foetal congenital anomaly in four studies [24,26,29,32]. Of these outcomes, only child small for gestational age for anytime lithium exposure is neutral to lithium; all the other outcomes consider lithium as a risk factor (Supplementary Figures S1–S7; Supplementary Table S2). However, the heterogeneity of the studies of three out of seven outcomes (lithium exposure at any time during pregnancy and any congenital anomaly and preterm birth, as well as lithium exposure during the first trimester of pregnancy and any congenital anomaly) was considerable, approaching a heterogeneity index I2 of about 50% for each of the outcomes above (Supplementary Table S2).

The small for gestational age outcome for exposure at any time to lithium is the only one that did not result to be a risk factor, at odds with large for gestational age for anytime exposure, which proved to be the one with the greatest deviation from the midline compared to the other outcomes. However, this outcome is based on only three studies, of which the one with the largest sample is the only one that does not cross the midline [30].

## 4. Discussion

The results emerging from this review and meta-analysis indicate that lithium treatment during pregnancy may worsen some outcomes at birth, but do not show that these outcomes are affected worse than if the underlying disorder is left untreated. Of the 28 studies eligible for our review, 13 reported no increased risk for birth outcomes, and 8 reported higher risk for some outcomes, mainly preterm birth or cardiovascular, while 6 were inconclusive/unsure, and 1 reported inadequacy for expressing a conclusion. While no firm conclusions can be drawn from the literature review, the meta-analysis allowed us to conclude that any time exposure to lithium during pregnancy is associated with increased risk of cardiac anomalies, any congenital anomaly, preterm birth, and large for gestational age, while exposure during the first trimester leads to cardiac and any congenital anomaly. However, these results cannot be plotted against the odds of having increased risk for the same outcomes for carrying out a pregnancy with no drug intervention while having BD or other psychiatric disorders needing lithium treatment. In fact, most comparative studies used samples on lithium compared with the general population or with other drugs, but not populations needing treatment with a given psychopathological condition who were or were not taking lithium treatment. The only study that compared pregnancy outcomes in patients with BD between those patients taking lithium and those without treatment found increased risk for adverse outcomes in patients with BD, independent of whether they were on lithium treatment or not [38].

Many women on lithium who learn they are pregnant discontinue lithium, despite a dearth of evidence as to the benefit of the discontinuation [50]. Although most experts agree to the need for continuous administration of lithium during pregnancy lest perilous recurrences occur [51,52,53], some patients find it difficult to obtain a prescription even after their second trimester [50]; doctors tend to prescribe it more intensely with the progression of pregnancy [54], probably because they know that pregnancy in itself reduces lithium levels [55,56]. Therefore, it appears that both patients and doctors share some responsibility for the reduction in prescribing lithium during pregnancy, although many physicians tend to increase doses with pregnancy [54]. Summarising, although lithium may be less teratogenic than what was held in the past [26,57], there is sufficient epidemiological evidence to suggest that its use during pregnancy is associated with adverse birth outcomes [58]. Among the most worrisome malformations that have been reported are those regarding the cardiovascular system [7,59]. We also found increased odds for lithium-associated cardiac malformations among mothers who were exposed to lithium (Supplementary Table S2).

The question of whether lithium is dangerous for the foetus or the newborn, which is the basic research question here, has strong clinical implications related to prescribing lithium to a pregnant woman, continuing with lithium prescription as the pregnancy continues, reinstating lithium during lactation, and strict monitoring of lithium blood levels. If lithium is free from possible teratogenicity, it should be prescribed as it is in non-pregnant women; conversely, if it is potentially harmful, the harm must be weighed against the risk for the foetus and the newborn of discontinuing, having an untreated psychiatric disorder, and using an alternative (such as other mood stabilisers or antipsychotic agents). Furthermore, many patients take lithium not only as a drug; hence foetal/newborn adverse outcomes cannot be attributed solely to the use of lithium. They also tend to adopt an unhealthy lifestyle, including alcohol and illicit drug use, which are per se teratogenic, rendering difficult the attempt to disentangle the effects of lithium intake [60]. All these considerations do not allow us to suggest clear-cut recommendations. Another question to address is whether adverse outcomes resolve spontaneously with lithium vs. being untreated with bipolar disorder vs. being treated for an illness with drugs that are known not to be teratogenic. Apparently in a large Israeli study [26], the outcomes did not differ, but when the authors included cases from Canadian and Australian databases, unfavourable statistical differences emerged for lithium [26,58], but again, the results might have been biased by the way the Australian site collected the data [58].

Uncertainty also reigns for concerns about whether lithium is per se teratogenic or not in human or animal studies. Lithium proved to be teratogenic in some but not in other species [61] and in some cell lines [62]; generally, the first reports were much more worrisome [63,64,65] than the most recent ones [66,67]. In one study conducted on rats, lithium hydroxybutyrate was found to be more generally toxic but less embryotoxic than lithium carbonate [68]. No formulation-dependent effects were found in humans [47]. While lithium is toxic to various organs in rats, no developmental or reproductive outcomes were found to be affected [67]. A similar trend towards a reduction in the role of lithium in teratogenicity has been shown for human studies [69,70].

The mechanisms by which lithium brings about adverse birth outcomes has not been explored specifically in humans, but some hypotheses were advanced, all based on the multiple mechanisms of lithium action in animals or in cell systems. It is generally recognised that lithium is more teratogenic in the first trimester rather than later in pregnancy [71]. This is thought to arise from the dysmorphogenetic properties of lithium during the earliest phases of development, i.e., in the first cell cycles, and this is shown to depend on the ability of lithium to impact the phosphoinositide cycle [72], which is a well-known pathway through which lithium acts, inhibiting key enzymes like inositol mono-phosphatase and inositol polyphosphate 1 phosphatase [73]. However, there are species differences, inasmuch lithium-induced maturational arrest in sea urchins is reversed by myo-inositol, which restores function in the phosphoinositide signalling pathway [72], while in rat embryos, myo-inositol was shown to be ineffective in countering the embryotoxic effect of lithium chloride [74]. Another relatively recently discovered mechanism of action of lithium is through interfering with the wingless Wnt-beta-catenin pathway, involving glycogen synthase-kinase 3-beta inhibition [75,76]. This interference was found to underlie lithium-induced alterations in morphogenesis in both eukaryotic cell systems [77], sea squirt eggs [78], zebrafish [79], and pig [80]. Early lithium exposure biases morphogenesis [81,82], thus increasing its teratogenic potential through shifting the site of the neural plate [83]. Early ectopic development of the neural plate, should it be confirmed in humans, may ensue in the syndrome identified as “floppy baby” [84]. Despite the fact that the placenta protects the foetus from lithium entry, most of the lithium that manages to cross the placenta may be found in the rat brain and affect its development [85]. Although teratogenicity is species-bound, the increased lithium teratogenicity reported in lower organisms compared to humans was probably due to different lithium dosages [79]. Teratogenicity is not set-off after the first trimester, but the additional risk for some adverse outcomes may be lower, as shown by our meta-analysis, where the significance for a risk of cardiac anomalies and any congenital anomaly increases only a little from lithium exposure during the first trimester to the cumulative risk of exposure at any time during pregnancy (Supplementary Table S2).

While the conclusions emerging from considering the entire range of eligible studies were a substantial draw between no increased risk and increased/unknown risk, with about half of the studies endorsing no increased risk for lithium use during pregnancy, our meta-analysis, which was based on 10 of the 28 eligible studies, was much more resolute in stating that the risk exists and regards several outcomes (Supplemental Materials, Figures S1–S7, Supplementary Table S2). Usually, meta-analyses follow systematic reviews on a given issue, yielding no discrepancy between conclusions, as one summarises evidence and the other synthesises it quantitatively. We hypothesised that the differences in result interpretation could be due to the risk of bias of the included studies. Among the 10 studies included in the meta-analysis, 2 studies had high risk of bias, 2 had some concerns, and 6 were at low risk; among the remaining 18 studies that were included in the systematic review, only 2 were at high risk, 10 had some concerns, and 6 were at low risk (Supplement, Figure S8). The difference was not statistically significant (*χ*^2^ = 3.3185; *p* = 0.1903). The Cochrane RoB tool we used [23] does not address publication bias; however, this bias does not appear to have influenced our results, since our search strategy did not exclude studies with negative results, nor those published in languages other than English, and we directly contacted authors of studies with unclear or partial data to obtain additional information so to reduce the risk of selective reporting bias. Whatever conclusion we might draw, the recommendations that we can make to clinicians consist in the same ones repeated time and again in most studies conducted on this issue, i.e., plan carefully with women with BD any pregnancy, do not discontinue lithium after knowing about the pregnancy, carefully monitor lithium blood levels, adjust the lithium dose accordingly, monitor how the foetus is developing through ultrasound examinations, reinstate lithium as soon as the patient delivers, perform follow-up visits including mother and baby, and weigh the pros and cons of lithium therapy vs. the risk of a BD relapse, which is equally, if not more, risky for the baby.

We should make clear here that we dealt with therapeutic lithium use without the presence of the cation per se. Lithium is one of the trace elements present in the human serum at very low concentrations (1.09 ± 0.63 ng∙mL^−1^) [86], i.e., about one millionth of its ideal therapeutic serum concentration. Non-therapeutic, physiological lithium intake in humans is mainly through drinking water and some vegetables [87]. High lithium levels in drinking water are associated with reduced suicide rates in the area where such lithium-enriched water is supplied [88] and increased longevity in humans [87] but also to reduced foetal size when the exposure occurs during pregnancy [89], whereas higher lithium levels in the mother’s umbilical cord serum were associated with poorer cognitive outcomes in 20–40-month-old children [90]. Therefore, it appears that lithium may both benefit and worsen human outcomes, but during pregnancy, it is likely to cause damage when exceeding certain levels that have yet to be defined. We did not find a relationship between lithium treatment during pregnancy at any time and small for gestational age foetal size, but on the contrary, with large for gestational age (Supplementary Table S2), at odds with the finding of Harari et al. [89].

Summarising the evidence, the recommendation that we feel we have to make to clinicians is not to stop lithium after the first trimester, as the potential damage is already done, and we cannot go back. Leaving the pregnant patient to the possible exposure to a new BD episode is worse than accepting the risks associated with lithium. Turning to another effective drug and switching lithium is similarly dangerous, as alternatives like valproate and carbamazepine carry more teratogenic potential than lithium itself. Dose adjustments may be useful, but not in the reduction direction, as pregnancy tends to lower lithium levels [55,56]; increasing the dose would be appropriate but should follow regular examinations of lithium levels.

### Limitations

The small number of studies that we could include in the meta-analysis limits the generalisability of our results. The risk of bias of most studies included in the systematic review was “some concerns”, while only twelve studies were at low risk of bias, i.e., less than half of the eligible studies. Besides this, our meta-analysis did not cover all the possible outcomes, as the studies focused only on some specific outcomes. The investigated outcomes were heterogeneous; hence, this limited their meta-analysability. Data on quantitative correlates were available only from a limited number of studies, hampering the precision of estimates. Similarly, some characteristics could not be explored due to the lack of sufficient data from eligible studies, thus preventing a more comprehensive assessment. Furthermore, quantitative data on important characteristics, including serum lithium concentration and the duration of lithium exposure, were not available from most of the studies. In addition, we were unable to conduct a sensitivity analysis, as the number of studies included for each of the six outcomes in our meta-analysis did not reach the minimum required for a sensitivity analysis to be performed, hence we were unable to control for some potential confounders. Although we took care to avoid possible selection and publication biases, we may not have avoided all of them, as publication bias lies behind every research step [91]. This review and meta-analysis aimed to establish whether taking lithium during pregnancy could represent a risk for congenital malformations in the foetus or newborn; the systematic review response is maybe, and the meta-analytic response is yes. However, to produce useful recommendations for clinicians who are puzzled as to whether prescribe lithium or not to a pregnant woman, we need to balance the evidence of the risk of adverse pregnancy outcomes with the risk of developing a new episode and its consequent risks on the same outcomes. This evidence is hard to obtain from currently published studies, as only one compared lithium-treated with untreated women with BD [38], while no study investigated the risk of switching to a BD episode and its effect on the same outcomes compared with lithium-related risks. We did not register our review. We did not base it on a protocol.

## 5. Conclusions

Women with BD being treated with lithium who are planning a pregnancy should discuss their plans with their doctors. If women become pregnant accidentally while on lithium, they should not stop taking it. Taking lithium during pregnancy, especially during the first trimester, is related to adverse birth outcomes for the foetus or the newborn, i.e., cardiac or any congenital malformation, preterm birth, and large for gestational age. These outcomes are similar to those of women affected with BD who are not taking lithium.

## Data Availability

Not applicable.

## Supplementary Materials

The following supporting information can be downloaded at: https://www.mdpi.com/article/10.3390/jcm13164872/s1, Figure S1. Association between lithium exposure (at any time) and cardiac anomalies in the foetuses of women with a psychiatric disorder: forest plot. Figure S2. Association between lithium exposure (at any time) and any congenital anomaly in the foetuses of women with a psychiatric disorder: forest plot. Figure S3. Association between lithium exposure (at any time) and preterm birth in women with a psychiatric disorder: forest plot. Figure S4. Association between lithium exposure (at any time) and child small for gestational age (SGA) in women with a psychiatric disorder: forest plot. Figure S5. Association between lithium exposure (at any time) and child large for gestational age (LGA) in women with a psychiatric disorder: forest plot. Figure S6. Association between lithium exposure (during the first trimester) and cardiac anomaly in the foetus of women with a psychiatric disorder: forest plot. Figure S7. Association between lithium exposure (during the first trimester) and any congenital anomaly in the foetus of women with a psychiatric disorder: forest plot. Figure S8. Risk of bias according to the Cochrane ROBINS-E tool. Supplementary Table S1: (Obstetric outcomes in women with Bipolar Disorder treated with lithium) OR ((outcome* AND (birth OR delivery OR pregnan* OR obstetric* OR fetal OR foetal)) AND “bipolar disorder” AND lithium) OR (pregnancy outcome AND lithium) PubMed 8 April 2024→ 117 results Cochrane Central Register of Controlled Trials; Cochrane Database of Systematic Reviews; Cochrane Methodology Register; Cochrane Clinical Answers 8 April 2024→ 60 records; Cinahl 8 April 2024→ 59 results; PsycINFO/PsycARTICLES/Psychology and Behavioral Sciences Collection 8 April 2024→ 76 records; ClinicalTrials.gov 8 April 2024 Condition/disease: pregnancy; Other terms: _; Intervention/treatment: Lithium → 15 records; From other sources 8 April 2024→ 45 records. Supplementary Table S2. Summary of findings. References for the meta-analysis. Risk of bias of the included studies notes. PRISMA CheckList.
