# Supplementary material for: Obstetric Outcomes in Women on Lithium: A Systematic Review and Meta-Analysis
Source: J Clin Med. 2024 Aug 18;13(16):4872. doi: 10.3390/jcm13164872 (PMC11355283; doi:10.3390/jcm13164872)
Supplement: Supplementary file 1 [file jcm-13-04872-s001.zip › jcm-3148582-supplementary.pdf]

Supplementary Table S1. (Obstetric outcomes in women with Bipolar Disorder treated with lithium) OR ((outcome* AND (birth OR delivery OR pregnan* OR obstetric* OR fetal OR foetal)) AND "bipolar disorder" AND lithium) OR (pregnancy outcome AND lithium) PubMed 8.4.2024 → 117 results Cochrane Central Register of Controlled Trials; Cochrane Database of Systematic Reviews; Cochrane Methodology Register; Cochrane Clinical Answers 8.4.2024 → 60 records; Cinahl 8.4.2024 → 59 results; PsycINFO/PsycARTICLES/Psychology and Behavioral Sciences Collection 8.4.2024 → 76 records; ClinicalTrials.gov 8.4.2024 Condition/disease: pregnancy; Other terms: _; Intervention/treatment: Lithium → 15 records; From other sources 8.4.2024 → 45 records

1	Stainbrook E. Clinical psychiatry. Prog Neurol Psychiatry. 1971;26:419-34. PMID: 5170807.	Opinion
2	Tuchmann-Duplessis H, Mercier-Parot L. Influence du lithium sur la gestation et le développement prénatal du rat et de la souris [Influence of lithium on gestation and prenatal development of the rat and mouse]. C R Seances Soc Biol Fil. 1973;167(2):183-6. French. PMID: 4214079.	Animal
3	Perera HV. A review of psychiatric aspects for termination of pregnancy. Ceylon Med J. 1983;28(1):42-7. PMID: 6667525.	Review
4	Källén B, Tandberg A. Lithium and pregnancy. A cohort study on manic- depressive women. Acta Psychiatr Scand. 1983;68(2):134-9. doi: 10.1111/j.1600-0447.1983.tb06991.x. PMID: 6624510.	**Included**
5	Niebyl JR. Drug therapy during pregnancy. Curr Opin Obstet Gynecol. 1992;4(1):43-7. PMID: 1543829.	Review
6	Jacobson SJ, Jones K, Johnson K, Ceolin L, Kaur P, Sahn D, Donnenfeld AE, Rieder M, Santelli R, Smythe J, et al. Prospective multicentre study of pregnancy outcome after lithium exposure during first trimester. Lancet. 1992;339(8792):530-3. doi: 10.1016/0140-6736(92)90346-5. PMID: 1346886.	**Included**
7	Austin MP. Puerperal affective psychosis: is there a case for lithium prophylaxis? Br J Psychiatry. 1992;161:692-4. doi: 10.1192/bjp.161.5.692. PMID: 1422621.	Unfocused
8	Glöckner R, Schwarz S, Jähne F. Enhanced effect of chronic stress on pregnancy outcome in Uje:WIST rats by prenatal treatment with lithium. Exp Toxicol Pathol. 1993;45(1):35-7. doi: 10.1016/S0940-2993(11)80449-5. PMID: 8467198.	Animal
9	Koren G, Graham K, Feigenbaum A, Einarson T. Evaluation and counseling of teratogenic risk: the motherisk approach. J Clin Pharmacol. 1993;33(5):405-11. doi: 10.1002/j.1552-4604.1993.tb04679.x. PMID: 8331196.	Unfocused
10	Cohen LS, Friedman JM, Jefferson JW, Johnson EM, Weiner ML. A reevaluation of risk of *in utero* exposure to lithium. JAMA. 1994;271(2):146-50. Erratum in: JAMA 1994;271(19):1485. PMID: 8031346.	Review
11	Keck P. Lithium: the present and the future. J Clin Psychiatry. 1995;56(1):41-8. PMID: 7836344.	Review
12	Altshuler LL, Cohen L, Szuba MP, Burt VK, Gitlin M, Mintz J. Pharmacologic management of psychiatric illness during pregnancy: dilemmas and guidelines. Am J Psychiatry. 1996;153(5):592-606. doi: 10.1176/ajp.153.5.592. PMID: 8615404.	Review
13	Loebstein R, Koren G. Pregnancy outcome and neurodevelopment of children exposed in utero to psychoactive drugs: the Motherisk experience. J Psychiatry Neurosci. 1997;22(3):192-6. PMID: 9183118; PMCID: PMC1188852. Unique identifier: 1997-04644-005.	Review
14	Austin MP, Mitchell PB. Psychotropic medications in pregnant women: treatment dilemmas. Med J Aust. 1998;169(8):428-31. doi: 10.5694/j.1326-5377.1998.tb126837.x. PMID: 9830392.	Review
15	Nicoll AE, Norman J, Macpherson A, Acharya U. Association of reduced selenium status in the aetiology of recurrent miscarriage. Br J Obstet Gynaecol. 1999;106(11):1188-91. doi: 10.1111/j.1471-0528.1999.tb08146.x. PMID: 10549965.	No lithium
16	Walker A, Rosenberg M, Balaban-Gil K. Neurodevelopmental and neurobehavioral sequelae of selected substances of abuse and psychiatric medications in utero. Child Adolesc Psychiatr Clin N Am. 1999;8(4):845-67. PMID: 10553207.	Review
17	Tekin M, Ellison J. Oromandibular-limb hypogenesis spectrum and maternal lithium use. Clin Dysmorphol. 2000;9(2):139-41. doi: 10.1097/00019605-200009020-00014. PMID: 10826629.	Case
18	Schou M. Cinquante ans de traitement par le lithium [50 years lithium treatment]. Encéphale. 2000;26(2):1-6. French. PMID: 10858908.	Review
19	Griswold KS, Pessar LF. Management of bipolar disorder. Am Fam Physician. 2000;62(6):1343-53, 1357-8. PMID: 11011863.	Review
20	Schou M. Perspectives on lithium treatment of bipolar disorder: action, efficacy, effect on suicidal behavior. Bipolar Disord. 1999;1(1):5-10. doi: 10.1034/j.1399-5618.1999.10103.x. PMID: 11256657.	Review
21	Baldessarini RJ, Tondo L, Hennen J, Viguera AC. Is lithium still worth using? An update of selected recent research. Harv Rev Psychiatry. 2002;10(2):59-75. PMID: 11897747.	Review
22	Ernst CL, Goldberg JF. The reproductive safety profile of mood stabilizers, atypical antipsychotics, and broad-spectrum psychotropics. J Clin Psychiatry. 2002;63(Suppl 4):42-55. PMID: 11913676.	Review
23	Shepard TH, Brent RL, Friedman JM, Jones KL, Miller RK, Moore CA, Polifka JE. Update on new developments in the study of human teratogens. Teratology. 2002;65(4):153-61. doi: 10.1002/tera.10032. PMID: 11948561.	Review
24	Bown CD, Wang JF, Young LT. Attenuation of N-methyl-D-aspartate-mediated cytoplasmic vacuolization in primary rat hippocampal neurons by mood stabilizers. Neuroscience. 2003;117(4):949-55. doi: 10.1016/s0306-4522(02)00743-1. PMID: 12654346.	Animal
25	Tschudin S, Lapaire O. Antidepressiva in der Schwangerschaft [Antidepressants and pregnancy]. Ther Umsch. 2005;62(1):17-22. German. doi: 10.1024/0040-5930.62.1.17. PMID: 15702702.	Review
26	Grunze H. Reevaluating therapies for bipolar depression. J Clin Psychiatry. 2005;66(Suppl 5):17-25. PMID: 16038598.	Review
27	Eberhard-Gran M, Eskild A, Opjordsmoen S. Treating mood disorders during pregnancy: safety considerations. Drug Saf. 2005;28(8):695-706. doi: 10.2165/00002018-200528080-00004. PMID: 16048355.	Review
28	Newport DJ, Viguera AC, Beach AJ, Ritchie JC, Cohen LS, Stowe ZN. Lithium placental passage and obstetrical outcome: implications for clinical management during late pregnancy. Am J Psychiatry. 2005;162(11):2162-70. doi: 10.1176/appi.ajp.162.11.2162. PMID: 16263858.	Unfocused
29	Chisholm D, van Ommeren M, Ayuso-Mateos JL, Saxena S. Cost-effectiveness of clinical interventions for reducing the global burden of bipolar disorder. Br J Psychiatry. 2005;187:559-67. doi: 10.1192/bjp.187.6.559. PMID: 16319409.	No pregnancy
30	Giles JJ, Bannigan JG. Teratogenic and developmental effects of lithium. Curr Pharm Des. 2006;12(12):1531-41. doi: 10.2174/138161206776389804. PMID: 16611133.	Review
31	Gentile S. Prophylactic treatment of bipolar disorder in pregnancy and breastfeeding: focus on emerging mood stabilizers. Bipolar Disord. 2006;8(3):207-20. doi: 10.1111/j.1399-5618.2006.00295.x. PMID: 16696822.	Review
32	Bauer MS, McBride L, Williford WO, Glick H, Kinosian B, Altshuler L, Beresford T, Kilbourne AM, Sajatovic M; Cooperative Studies Program 430 Study Team. Collaborative care for bipolar disorder: part I. Intervention and implementation in a randomized effectiveness trial. Psychiatr Serv. 2006;57(7):927-36. doi: 10.1176/ps.2006.57.7.927. PMID: 16816276.	No pregnancy
33	Even C, Dorocant ES, Thuile J, Kalck-Stern M, Guelfi JD. Grossesse, allaitement et thymorégulateurs: eléments de décisions et règles pour la pratique [Pregnancy, breast feeding and mood stabilisers: review and recommendations for practice]. Encéphale. 2006;32(2 Pt 1):224-30. French. doi: 10.1016/s0013-7006(06)76148-6. PMID: 16910623.	Review
34	Dhikav V, Anand KS. Is hippocampal atrophy a future drug target? Med Hypotheses. 2007;68(6):1300-6. doi: 10.1016/j.mehy.2006.09.040. Epub 2006 Nov 13. PMID: 17098374.	Opinion
35	Ward S, Wisner KL. Collaborative management of women with bipolar disorder during pregnancy and postpartum: pharmacologic considerations. J Midwifery Womens Health. 2007;52(1):3-13. doi: 10.1016/j.jmwh.2006.09.002. PMID: 17207745.	Review
36	De las Cuevas C, de la Rosa M A, Troyano JM, Sanz EJ. Are psychotropics drugs used in pregnancy? Pharmacoepidemiol Drug Saf. 2007;16(9):1018-23. doi: 10.1002/pds.1401. PMID: 17455181.	No birth outcomes
37	Simhandl C, Mersch J. Lithium und bipolare Erkrankung—eine Renaissance? [Lithium and bipolar disorder--a renaissance?]. Neuropsychiatr. 2007;21(2):121-30. German. PMID: 17640498.	Review
38	De Santis M, Cesari E, Ligato MS, Nobili E, Straface G, Cavaliere A, Caruso A. Prenatal drug exposure and teratological risk: one-year experience of an Italian Teratology Information Service. Med Sci Monit. 2008;14(2):PH1-8. PMID: 18227771.	Opinion
39	Blake LD, Lucas DN, Aziz K, Castello-Cortes A, Robinson PN. Lithium toxicity and the parturient: case report and literature review. Int J Obstet Anesth. 2008;17(2):164-9. doi: 10.1016/j.ijoa.2007.09.014. Epub 2008 Mar 4. PMID: 18308554.	Case
40	Reis M, Källén B. Maternal use of antipsychotics in early pregnancy and delivery outcome. J Clin Psychopharmacol. 2008;28(3):279-88. doi: 10.1097/JCP.0b013e318172b8d5. PMID: 18480684.	**Included**
41	Dodd S, Berk M. The safety of medications for the treatment of bipolar disorder during pregnancy and the puerperium. Curr Drug Saf. 2006;1(1):25-33. doi: 10.2174/157488606775252692. PMID: 18690912.	Review
42	Jürgens TP, Schaefer C, May A. Treatment of cluster headache in pregnancy and lactation. Cephalalgia. 2009;29(4):391-400. doi: 10.1111/j.1468-2982.2008.01764.x. Epub 2009 Jan 19. PMID: 19170693.	Opinion
43	Grandjean EM, Aubry JM. Lithium: updated human knowledge using an evidence- based approach: part III: clinical safety. CNS Drugs. 2009;23(5):397-418. doi: 10.2165/00023210-200923050-00004. PMID: 19453201.	Review

44	Howland RH. Prescribing psychotropic medications during pregnancy and lactation: principles and guidelines. J Psychosoc Nurs Ment Health Serv. 2009;47(5):19-23. doi: 10.3928/02793695-20090331-05. PMID: 19489510.	Opinion
45	Gentile S. Neurodevelopmental effects of prenatal exposure to psychotropic medications. Depress Anxiety. 2010;27(7):675-86. doi: 10.1002/da.20706. PMID: 20583298.	Review
46	Burt VK, Bernstein C, Rosenstein WS, Altshuler LL. Bipolar disorder and pregnancy: maintaining psychiatric stability in the real world of obstetric and psychiatric complications. Am J Psychiatry. 2010;167(8):892-7. doi: 10.1176/appi.ajp.2009.09081248. PMID: 20693466.	Case
47	Wolff EF, Hughes M, Merino MJ, Reynolds JC, Davis JL, Cochran CS, Celi FS. Expression of benign and malignant thyroid tissue in ovarian teratomas and the importance of multimodal management as illustrated by a BRAF-positive follicular variant of papillary thyroid cancer. Thyroid. 2010;20(9):981-7. doi: 10.1089/thy.2009.0458. PMID: 20718682; PMCID: PMC2964358.	Review
48	Galbally M, Roberts M, Buist A; Perinatal Psychotropic Review Group. Mood stabilizers in pregnancy: a systematic review. Aust N Z J Psychiatry. 2010;44(11):967-77. doi: 10.3109/00048674.2010.506637. PMID: 21034180.	Review
49	Bergink V, Kushner SA, Pop V, Kuijpens H, Lambregtse-van den Berg MP, Drexhage RC, Wiersinga W, Nolen WA, Drexhage HA. Prevalence of autoimmune thyroid dysfunction in postpartum psychosis. Br J Psychiatry. 2011;198(4):264-8. doi: 10.1192/bjp.bp.110.082990. Epub 2011 Feb 22. PMID: 21343331.	Unfocused
50	Apostoli P, Catalani S. Metal ions affecting reproduction and development. Met Ions Life Sci. 2011;8:263-303. PMID: 21473384.	Review
51	Vieta E, Colom F. Therapeutic options in treatment-resistant depression. Ann Med. 2011;43(7):512-30. doi: 10.3109/07853890.2011.583675. Epub 2011 May 31. PMID: 21623700.	Review
52	Bergink V, Lambregtse-van den Berg MP, Koorengevel KM, Kupka R, Kushner SA. First-onset psychosis occurring in the postpartum period: a prospective cohort study. J Clin Psychiatry. 2011;72(11):1531-7. doi: 10.4088/JCP.10m06648. Epub 2011 Aug 23. PMID: 21903022.	No birth outcomes
53	van der Lugt NM, van de Maat JS, van Kamp IL, Knoppert-van der Klein EA, Hovens JG, Walther FJ. Fetal, neonatal and developmental outcomes of lithium-exposed pregnancies. Early Hum Dev. 2012;88(6):375-8. doi: 10.1016/j.earlhumdev.2011.09.013. Epub 2011 Oct 14. PMID: 22000820.	**Included**
54	McKnight RF, Adida M, Budge K, Stockton S, Goodwin GM, Geddes JR. Lithium toxicity profile: a systematic review and meta-analysis. Lancet. 2012;379(9817):721-8. doi: 10.1016/S0140-6736(11)61516-X. Epub 2012 Jan 20. PMID: 22265699.	Review
55	Gentile S. Lithium in pregnancy: the need to treat, the duty to ensure safety. Expert Opin Drug Saf. 2012;11(3):425-37. doi: 10.1517/14740338.2012.670419. Epub 2012 Mar 9. PMID: 22400907.	Review
56	Kim HG. A piece of my mind. Drowning in plain sight. JAMA. 2012;307(18):1923-4. doi: 10.1001/jama.2012.3445. PMID: 22570461.	Opinion
57	Frassanito L, Vagnoni S, Zanfini BA, Catarci S, Maggiore S, Draisci G. General anesthesia for caesarean delivery in a pregnant woman affected by acute myocardial infarction. Eur Rev Med Pharmacol Sci. 2012;16(8):1123-6. PMID: 22913165.	Case
58	Bodén R, Lundgren M, Brandt L, Reutfors J, Andersen M, Kieler H. Risks of adverse pregnancy and birth outcomes in women treated or not treated with mood stabilisers for bipolar disorder: population based cohort study. BMJ. 2012;345:e7085. doi: 10.1136/bmj.e7085. PMID: 23137820; PMCID: PMC3493986.	**Included**
59	Frayne J, Nguyen T, Kohan R, De Felice N, Rampono J. The comprehensive management of pregnant women with major mood disorders: a case study involving phenelzine, lithium, and quetiapine. Arch Womens Ment Health. 2014;17(1):73-5. doi: 10.1007/s00737-013-0386-3. Epub 2013 Nov 8. PMID: 24196828.	Case
60	Deiana V, Chillotti C, Manchia M, Carta P, Bocchetta A, Ardau R, Del Zompo M. Continuation versus discontinuation of lithium during pregnancy: a retrospective case series. J Clin Psychopharmacol. 2014;34(3):407-10. doi: 10.1097/JCP.0000000000000059. PMID: 24525653.	Case
61	Diav-Citrin O, Shechtman S, Tahover E, Finkel-Pekarsky V, Arnon J, Kennedy D, Erebara A, Einarson A, Ornoy A. Pregnancy outcome following in utero exposure to lithium: a prospective, comparative, observational study. Am J Psychiatry. 2014;171(7):785-94. doi: 10.1176/appi.ajp.2014.12111402. PMID: 24781368.	**Included**
62	Bergink V, Kushner SA. Lithium during pregnancy. Am J Psychiatry. 2014;171(7):712-5. doi: 10.1176/appi.ajp.2014.14030409. PMID: 24980165.	Opinion
63	Marinò M, Latrofa F, Menconi F, Chiovato L, Vitti P. An update on the medical treatment of Graves' hyperthyroidism. J Endocrinol Invest. 2014;37(11):1041-8. doi: 10.1007/s40618-014-0136-z. Epub 2014 Sep 4. PMID: 25185644.	Review
64	Huhta JC, Linask K. When should we prescribe high-dose folic acid to prevent congenital heart defects? Curr Opin Cardiol. 2015;30(1):125-31. doi: 10.1097/HCO.0000000000000124. PMID: 25389654.	Review
65	Wesseloo R, Burgerhout KM, Koorengevel KM, Bergink V. Postpartumpsychose in de klinische praktijk: diagnostiek, behandeling en preventie [Postpartum psychosis in clinical practice: diagnostic considerations, treatment and prevention]. Tijdschr Psychiatr. 2015;57(1):25-33. Dutch. PMID: 25601625.	Review
66	Costoloni G, Pierantozzi E, Goracci A, Bolognesi S, Fagiolini A. Leki normotymiczne a ciąża - przegląd [Mood stabilisers and pregnancy outcomes – a review]. Psychiatr Pol. 2014;48(5):865-87. Polish. doi: 10.12740/pp/25834. PMID: 25639010.	Review
67	Rosso G, Albert U, Di Salvo G, Scatà M, Todros T, Maina G. Lithium prophylaxis during pregnancy and the postpartum period in women with lithium- responsive bipolar I disorder. Arch Womens Ment Health. 2016;19(2):429-32. doi: 10.1007/s00737-016-0601-0. Epub 2016 Jan 20. PMID: 26790685.	**Included**
68	Shansis FM, Reche M, Capp E. Evaluating response to mood stabilizers in patients with mixed depression: A study of agreement between three different mania rating scales and a depression rating scale. J Affect Disord. 2016;197:1-7. doi: 10.1016/j.jad.2016.02.064. Epub 2016 Mar 3. PMID: 26950019.	No birth outcomes
69	Petersen I, McCrea RL, Sammon CJ, Osborn DP, Evans SJ, Cowen PJ, Freemantle N, Nazareth I. Risks and benefits of psychotropic medication in pregnancy: cohort studies based on UK electronic primary care health records. Health Technol Assess. 2016;20(23):1-176. doi: 10.3310/hta20230. PMID: 27029490; PMCID: PMC4827034.	**Included**
70	Igra AM, Harari F, Lu Y, Casimiro E, Vahter M. Boron exposure through drinking water during pregnancy and birth size. Environ Int. 2016;95:54-60. doi: 10.1016/j.envint.2016.07.017. Epub 2016 Aug 6. PMID: 27502898.	Unfocused
71	Boyle B, Garne E, Loane M, Addor MC, Arriola L, Cavero-Carbonell C, Gatt M, Lelong N, Lynch C, Nelen V, Neville AJ, O'Mahony M, Pierini A, Rissmann A, Tucker D, Zymak-Zakutnia N, Dolk H. The changing epidemiology of Ebstein's anomaly and its relationship with maternal mental health conditions: a European registry-based study. Cardiol Young. 2017;27(4):677-685. doi: 10.1017/S1047951116001025. Epub 2016 Aug 30. PMID: 27572669.	**Included**
72	Leong C, Raymond C, Château D, Dahl M, Alessi-Severini S, Falk J, Bugden S, Katz A. Psychotropic Drug Use before, during, and after Pregnancy: A Population- Based Study in a Canadian Cohort (2001-2013). Can J Psychiatry. 2017;62(8):543-550. doi: 10.1177/0706743717711168. Epub 2017 May 25. PMID: 28545329; PMCID: PMC5546669.	Unfocused
73	Hatters Friedman S, Moller-Olsen C, Prakash C, North A. Atypical antipsychotic use and outcomes in an urban maternal mental health service. Int J Psychiatry Med. 2016;51(6):521-533. doi: 10.1177/0091217417696739. Epub 2017 Mar 6. PMID: 28629296.	Unfocused
74	Haskey C, Galbally M. Mood stabilizers in pregnancy and child developmental outcomes: A systematic review. Aust N Z J Psychiatry. 2017;51(11):1087-1097. doi: 10.1177/0004867417726175. Epub 2017 Aug 20. PMID: 28825316.	Review
75	Scrandis DA. Bipolar disorder in pregnancy: A review of pregnancy outcomes. J Midwifery Womens Health. 2017;62(6):673-683. doi: 10.1111/jmwh.12645. Epub 2017 Oct 30. PMID: 29083536.	Review
76	Forsberg L, Adler M, Römer Ek I, Ljungdahl M, Navér L, Gustafsson LL, Berglund G, Chotigasatien A, Hammar U, Böhm B, Wide K. Maternal mood disorders and lithium exposure in utero were not associated with poor cognitive development during childhood. Acta Paediatr. 2018;107(8):1379-1388. doi: 10.1111/apa.14152. Epub 2017 Dec 5. PMID: 29150869.	**Included**
77	Thomson M, Sharma V. Weighing the risks: the management of bipolar disorder during pregnancy. Curr Psychiatry Rep. 2018;20(3):20. doi: 10.1007/s11920-018-0882-2. PMID: 29549608.	Review
78	Neri C, De Luca C, D'oria L, Licameli A, Nucci M, Pellegrino M, Caruso A, De Santis M. Managing fertile women under lithium treatment: the challenge of a Teratology Information Service. Minerva Ginecol. 2018;70(3):261-267. doi: 10.23736/S0026-4784.18.04190-4. PMID: 29845825.	**Included**
79	Munk-Olsen T, Liu X, Viktorin A, Brown HK, Di Florio A, D'Onofrio BM, Gomes T, Howard LM, Khalifeh H, Krohn H, Larsson H, Lichtenstein P, Taylor CL, Van Kamp I, Wesseloo R, Meltzer-Brody S, Vigod SN, Bergink V. Maternal and infant outcomes associated with lithium use in pregnancy: an international collaborative meta-analysis of six cohort studies. Lancet Psychiatry. 2018;5(8):644-652. doi: 10.1016/S2215-0366(18)30180-9. Epub 2018 Jun 18. PMID: 29929874; PMCID: PMC6077091.	**Included**
80	Clark CT, Wisner KL. Treatment of peripartum bipolar disorder. Obstet Gynecol Clin North Am. 2018;45(3):403-417. doi: 10.1016/j.ogc.2018.05.002. PMID: 30092918; PMCID: PMC6548543.	Review
81	Fava M, Freeman MP, Flynn M, Judge H, Hoeppner BB, Cusin C, Ionescu DF, Mathew SJ, Chang LC, Iosifescu DV, Murrough J, Debattista C, Schatzberg AF, Trivedi MH, Jha MK, Sanacora G, Wilkinson ST, Papakostas GI. Double-blind, placebo-controlled, dose-ranging trial of intravenous ketamine as adjunctive therapy in treatment-resistant depression (TRD). Mol Psychiatry. 2020;25(7):1592-1603. doi: 10.1038/s41380-018-0256-5. Epub 2018 Oct 3. Erratum in: Mol Psychiatry. 2019 Jan 7; PMID: 30283029; PMCID: PMC6447473.	Unrelated
82	Galbally M, Frayne J, Watson SJ, Snellen M. Psychopharmacological prescribing practices in pregnancy for women with severe mental illness: A multicentre study. Eur Neuropsychopharmacol. 2019;29(1):57-65. doi: 10.1016/j.euroneuro.2018.11.1103. Epub 2018 Nov 26. PMID: 30497841.	Unfocused

83	Poels EMP, Bijma HH, Galbally M, Bergink V. Lithium during pregnancy and after delivery: a review. Int J Bipolar Disord. 2018;6(1):26. doi: 10.1186/s40345-018-0135-7. PMID: 30506447; PMCID: PMC6274637.	Review
84	McLean K, Murphy KE, Dalfen A, Shea AK. The effect of maternal antidepressants on third trimester uteroplacental hemodynamics and the neonatal abstinence syndrome: a retrospective cohort study. Arch Womens Ment Health. 2019;22(6):791-797. doi: 10.1007/s00737-019-00954-8. Epub 2019 Feb 23. PMID: 30798375.	Unfocused
85	Newmark RL, Bogen DL, Wisner KL, Isaac M, Ciolino JD, Clark CT. Risk-Benefit assessment of infant exposure to lithium through breast milk: a systematic review of the literature. Int Rev Psychiatry. 2019;31(3):295-304. doi: 10.1080/09540261.2019.1586657. Epub 2019 Jun 10. PMID: 31180257.	Review
86	Cohen JM, Huybrechts KF, Patorno E, Desai RJ, Mogun H, Bateman BT, Hernández-Díaz S. Anticonvulsant mood stabilizer and lithium use and risk of adverse pregnancy outcomes. J Clin Psychiatry. 2019;80(4):18m12572. doi: 10.4088/JCP.18m12572. PMID: 31237992.	Lumping
87	Anmella G, Pacchiarotti I, Cubała WJ, Dudek D, Maina G, Thomas P, Vieta E. Expert advice on the management of valproate in women with bipolar disorder at childbearing age. Eur Neuropsychopharmacol. 2019;29(11):1199-1212. doi: 10.1016/j.euroneuro.2019.09.007. Epub 2019 Oct 4. PMID: 31590972.	Review
88	Fornaro M, Maritan E, Ferranti R, Zaninotto L, Miola A, Anastasia A, Murru A, Solé E, Stubbs B, Carvalho AF, Serretti A, Vieta E, Fusar-Poli P, McGuire P, Young AH, Dazzan P, Vigod SN, Correll CU, Solmi M. Lithium exposure during pregnancy and the postpartum period: A systematic review and meta-analysis of safety and efficacy outcomes. Am J Psychiatry. 2020;177(1):76-92. doi: 10.1176/appi.ajp.2019.19030228. Epub 2019 Oct 18. PMID: 31623458.	Review
89	Hermann A, Gorun A, Benudis A. Lithium use and non-use for pregnant and postpartum women with bipolar disorder. Curr Psychiatry Rep. 2019;21(11):114. doi: 10.1007/s11920-019-1103-3. PMID: 31701245.	Review
90	Solé E, Roca A, Torres A, Hernández AS, Fernández N, Díaz CN, Vieta E, Garcia-Esteve L. Obstetric complications in bipolar disorder: Psychiatric factors and the risk of caesarean section. Eur Neuropsychopharmacol. 2020;32:47-55. doi: 10.1016/j.euroneuro.2019.12.115. Epub 2020 Jan 3. PMID: 31911063.	Unfocused
91	Albertini E, Ernst CL, Tamaroff RS. Psychopharmacological decision making in bipolar disorder during pregnancy and lactation: A case-by-case approach to using current evidence. Focus (Am Psychiatr Publ). 2019;17(3):249-258. doi: 10.1176/appi.focus.20190007. Epub 2019 Jul 16. PMID: 32047370; PMCID: PMC6999216.	Review
92	Lebedevs T, Gan M, Teoh SWK, Brown P. Analysis of perinatal women attending a mother and baby unit taking sodium valproate or lithium with a diagnosis of bipolar affective disorder. Psychiatr Q. 2020;91(3):695-701. doi: 10.1007/s11126-020-09729-2. PMID: 32157548.	Unfocused
93	Sharma V, Sharma P, Sharma S. Managing bipolar disorder during pregnancy and the postpartum period: a critical review of current practice. Expert Rev Neurother. 2020;20(4):373-383. doi: 10.1080/14737175.2020.1743684. Epub 2020 Mar 19. PMID: 32172610.	Review
94	Fuchs C, Bridler R. Lithium während der Schwanger-schaft und in der postpartalen-Phase bei Frauen mit einer bipolaren Störung – eine Metaanalyse zur Wirksamkeit und Sicherheit. Praxis (Bern 1994). 2020;109(7):551-552. German. doi: 10.1024/1661-8157/a003469. PMID: 32456585.	Review
95	Jabbi M, Arasappan D, Eickhoff SB, Strakowski SM, Nemeroff CB, Hofmann HA. Neuro-transcriptomic signatures for mood disorder morbidity and suicide mortality. J Psychiatr Res. 2020;127:62-74. doi: 10.1016/j.jpsychires.2020.05.013. Epub 2020 May 18. PMID: 32485434.	No pregnancy
96	Molenaar NM, Poels EMP, Robakis T, Wesseloo R, Bergink V. Management of lithium dosing around delivery: An observational study. Bipolar Disord. 2021;23(1):49-54. doi: 10.1111/bdi.12955. Epub 2020 Jun 30. PMID: 32526071; PMCID: PMC7891390.	**Included**
97	Poels EMP, Kamperman AM, Vreeker A, Gilden J, Boks MP, Kahn RS, Ophoff RA, Bergink V. Lithium use during pregnancy and the risk of miscarriage. J Clin Med. 2020;9(6):1819. doi: 10.3390/jcm9061819. PMID: 32545238; PMCID: PMC7356743.	**Included**
98	Lorettu L, Carpita B, Nivoli A, Milia P, De Iorio G, Cremone IM, Dell'Osso L. Lithium use during pregnancy in a patient with bipolar disorder and multiple sclerosis. Clin Neuropharmacol. 2020;43(5):158-161. doi: 10.1097/WNF.0000000000000407. PMID: 32947427.	Case
99	Kirsch DE, Tretyak V, Radpour S, Weber WA, Nemeroff CB, Fromme K, Strakowski SM, Lippard ETC. Childhood maltreatment, prefrontal-paralimbic gray matter volume, and substance use in young adults and interactions with risk for bipolar disorder. Sci Rep. 2021;11(1):123. doi: 10.1038/s41598-020-80407-w. PMID: 33420255; PMCID: PMC7794246.	Unrelated
100	Cuomo A, Amore M, Vampini C, Fagiolini A. Lamotrigina nel disturbo bipolare: prevenire la depressione per curare la malattia [Lamotrigine in bipolar disorder: preventing depression to treat the disease]. Riv Psichiatr. 2021;56(1):1-11. Italian. doi: 10.1708/3546.35215. PMID: 33560270.	Review
101	Mukunya D, Tumwine JK, Nankabirwa V, Odongkara B, Tongun JB, Arach AA, Tumuhamye J, Napyo A, Zalwango V, Achora V, Musaba MW, Ndeezi G, Tylleskär T. Neonatal hypothermia in Northern Uganda: a community-based cross-sectional study. BMJ Open. 2021;11(2):e041723. doi: 10.1136/bmjopen-2020-041723. PMID: 33574146; PMCID: PMC7880091.	No lithium
102	Viswanathan M, Middleton JC, Stuebe A, Berkman N, Goulding AN, McLaurin- Jiang S, Dotson AB, Coker-Schwimmer M, Baker C, Voisin C, Bann C, Gaynes BN. Maternal, Fetal, and Child Outcomes of Mental Health Treatments in Women: A Systematic Review of Perinatal Pharmacologic Interventions [Internet]. Rockville (MD): Agency for Healthcare Research and Quality (US); 2021 Apr. Report No.: 21-EHC001. PMID: 33950611.	Review
103	Imaz ML, Soy D, Torra M, García-Esteve L, Soler C, Martin-Santos R. Case report: Clinical and pharmacokinetic profile of lithium monotherapy in exclusive breastfeeding. A follow-up case series. Front Pharmacol. 2021;12:647414. doi: 10.3389/fphar.2021.647414. PMID: 34248617; PMCID: PMC8264295.	Case
104	Gilden J, Poels EMP, Lambrichts S, Vreeker A, Boks MPM, Ophoff RA, Kahn RS, Kamperman AM, Bergink V. Bipolar episodes after reproductive events in women with bipolar I disorder, a study of 919 pregnancies. J Affect Disord. 2021;295:72-79. doi: 10.1016/j.jad.2021.08.006. Epub 2021 Aug 8. PMID: 34416620.	Unfocused
105	Poels EMP, Schrijver L, White TJH, Roza SJ, Zarchev MG, Bijma H, Honig A, van Kamp IL, Hoogendijk WJG, Kamperman AM, Bergink V. The effect of prenatal lithium exposure on the neuropsychological development of the child. Bipolar Disord. 2022;24(3):310-319. doi: 10.1111/bdi.13133. Epub 2021 Oct 5. PMID: 34585812; PMCID: PMC9293321.	**Included**
106	Hastie R, Tong S, Hiscock R, Lindquist A, Lindström L, Wikström AK, Sundström-Poromaa I. Maternal lithium use and the risk of adverse pregnancy and neonatal outcomes: a Swedish population-based cohort study. BMC Med. 2021;19(1):291. doi: 10.1186/s12916-021-02170-7. PMID: 34856987; PMCID: PMC8641220.	**Included**
107	Mokhtare A, Davaji B, Xie P, Yaghoobi M, Rosenwaks Z, Lal A, Palermo G, Abbaspourrad A. Non-contact ultrasound oocyte denudation. Lab Chip. 2022;22(4):777-792. doi: 10.1039/d1lc00715g. PMID: 35075469.	Unrelated
108	Kan ACO, Chan JKN, Wong CSM, Chen EYH, Chang WC. Psychotropic drug utilization patterns in pregnant women with bipolar disorder: A 16-year population-based cohort study. Eur Neuropsychopharmacol. 2022;57:75-85. doi: 10.1016/j.euroneuro.2022.01.115. Epub 2022 Feb 14. PMID: 35151952.	No birth outcomes
109	Sagué-Vilavella M, Solé E, Pinzón-Espinosa J, Sandra-Hernández A, Roda E, Vieta E, Roca A. Obstetric outcomes regarding the use of lithium in pregnant women with bipolar disorders: a prospective cohort study. Arch Womens Ment Health. 2022;25(4):729-737. doi: 10.1007/s00737-022-01234-8. Epub 2022 May 6. PMID: 35522327.	**Included**
110	Wei L, Huang H, Chen X, Wang X, Zhang R, Su L, Duan W, Rahman M, Golam Mostofa M, Qamruzzaman Q, Shen H, Hu Z, Wei Y, Christiani DC, Chen F. Umbilical cord serum elementomics of 52 trace elements and early childhood neurodevelopment: Evidence from a prospective birth cohort in rural Bangladesh. Environ Int. 2022;166:107370. doi: 10.1016/j.envint.2022.107370. Epub 2022 Jun 24. PMID: 35772314; PMCID: PMC9926395.	No pregnancy
111	Torfs M, Hompes T, Ceulemans M, Van Calsteren K, Vanhole C, Smits A. Early postnatal outcome and care after in utero exposure to lithium: A single center analysis of a Belgian tertiary university hospital. Int J Environ Res Public Health. 2022;19(16):10111. doi: 10.3390/ijerph191610111. PMID: 36011745; PMCID: PMC9407712.	**Included**
112	Poels EMP, Kamperman AM, Bijma HH, Honig A, van Kamp IL, Kushner SA, Hoogendijk WJG, Bergink V, White T. Brain development after intrauterine exposure to lithium: A magnetic resonance imaging study in school-age children. Bipolar Disord. 2023;25(3):181-190. doi: 10.1111/bdi.13297. Epub 2023 Jan 20. PMID: 36633504.	**Included**
113	Álvarez-Silvares E, Fernández-Cruz T, Bermudez-González M, Rubio-Cid P, Almeida A, Pinto E, Seoane-Pillado T, Martínez-Carballo E. Placental levels of essential and non-essential trace element in relation to neonatal weight in Northwestern Spain: application of generalized additive models. Environ Sci Pollut Res Int. 2023;30(22):62566-62578. doi: 10.1007/s11356-023-26560-x. Epub 2023 Mar 21. PMID: 36943567.	**Included**
114	Schonewille NN, Terpstra PA, van den Heuvel MEN, Van Pampus MG, van den Heuvel OA, Broekman BFP. Neonatal admission after lithium use in pregnant women with bipolar disorders: a retrospective cohort study. Int J Bipolar Disord. 2023;11(1):24. doi: 10.1186/s40345-023-00306-7. PMID: 37450192; PMCID: PMC10348961.	**Included**
115	Whaites Heinonen E, Tötterman K, Bäck K, Sarman I, Forsberg L, Svedenkrans J. High lithium concentration at delivery is a potential risk factor for adverse outcomes in breastfed infants: a retrospective cohort study. Int J Bipolar Disord. 2023;11(1):36. doi: 10.1186/s40345-023-00317-4. PMID: 38032417; PMCID: PMC10689698.	**Included**
116	Schrijver L, Kamperman AM, Bijma H, van Kamp IL, Wesseloo R, Hoogendijk WJG, Bergink V, Poels EMP. Dose response relationship between lithium serum levels during pregnancy and birth outcomes. Acta Psychiatr Scand. 2024;149(4):323-331. doi: 10.1111/acps.13663. Epub 2024 Jan 18. PMID: 38238613.	**Included**
117	Stoppa A, Roda E, Garcia-Esteve L, Roca-Lecumberri A. A case of pregnancy with severe polyhydramnios related to long-term use of lithium. Bipolar Disord. 2024. doi: 10.1111/bdi.13427. Epub ahead of print 2024 Mar 26. PMID: 38529718.	Case

118	Bauer MS, McBride L, Williford WO, Glick H, Kinosian B, Altshuler L, Beresford T, Kilbourne AM, Sajatovic M; Cooperative Studies Program 430 Study Team. Collaborative care for bipolar disorder: part I. Intervention and implementation in a randomized effectiveness trial. Psychiatr Serv. 2006;57(7):927-36. doi: 10.1176/ps.2006.57.7.927. PMID: 16816276.	Duplicate= 32 PM
119	Pollack M, Mangano R, Entsuah R, Tzanis E, Simon NM, Zhang Y. A randomized controlled trial of venlafaxine ER and paroxetine in the treatment of outpatients with panic disorder. Psychopharmacology (Berl). 2007;194(2):233-42. doi: 10.1007/s00213-007-0821-0. Epub 2007 Jun 23. PMID: 17589833.	Unrelated
120	Walling AD. Does valproate prevent relapse in patients with bipolar I disorder? Am Fam Physician. 2010;81(7):827-8.	No pregnancy
121	NCT00140179. Valnoctamide in Mania. https://clinicaltrials.gov/show/NCT00140179, 2005 | added to CENTRAL: 31 May 2018 | 2018 Issue 5 CT.gov. Valnoctamide in Mania. ClinicalTrials.gov ID NCT00140179. Sponsor Beersheva Mental Health Center. Information provided by Beersheva Mental Health Center. Last Update Posted 2009-11-25.	Unrelated
122	NCT00475137. Lamotrigine Alone Compared to Lamotrigine Plus Antidepressant for the Treatment of Bipolar II Depression. https://clinicaltrials.gov/show/NCT00475137, 2007 | added to CENTRAL: 31 May 2018 | 2018 Issue 5 CT.gov. Lamotrigine Alone Compared to Lamotrigine Plus Antidepressant for the Treatment of Bipolar II Depression. ClinicalTrials.gov ID NCT00475137. Sponsor University of British Columbia. Information provided by University of British Columbia (Responsible Party). Last Update Posted 2015-06-04	No pregnancy
123	NCT02398487. Personal Vaporizer vs Cigalike. https://clinicaltrials.gov/show/NCT02398487, 2014 | added to CENTRAL: 31 May 2018 | 2018 Issue 5 CT.gov Personal Vaporizer vs Cigalike (VAPECIG 2) ClinicalTrials.gov ID NCT02398487 Sponsor Universita degli Studi di Catania. Information provided by Riccardo Polosa, Universita degli Studi di Catania (Responsible Party). Last Update Posted 2015-12-02	No pregnancy
124	NCT01855217. Pilot Study on the Pharmacodynamics of Sugammadex in Morbidly Obese Patients: reversal of Deep Neuromuscular Blockade. https://clinicaltrials.gov/show/NCT01855217, 2013 | added to CENTRAL: 31 May 2018 | 2018 Issue 5 CT.gov Pilot Study on the Pharmacodynamics of Sugammadex in Morbidly Obese Patients: Reversal of Deep Neuromuscular Blockade (MOS). ClinicalTrials.gov ID NCT01855217. Sponsor St. Antonius Hospital. Information provided by A. Houwink, St. Antonius Hospital (Responsible Party). Last Update Posted 2013-05-17	No pregnancy
125	NCT01926041 .The Effectiveness of Smoking Cessation in Prediabetic Smokers. https://clinicaltrials.gov/show/NCT01926041, 2013 | added to CENTRAL: 31 May 2018 | 2018 Issue 5 CT.gov.	No pregnancy
126	Cochrane Central Register of Controlled Trials. The Effect of Sertraline In Treatment Of Schizophrenia. IRCT2014041317246N1. https://trialsearch.who.int/Trial2.aspx?TrialID=IRCT2014041317246N1, 2014 | added to CENTRAL: 31 March 2019 | 2019 Issue 3	No pregnancy
127	IRCT2012111211438N1. Effect of fluvoxamine in pruritus in hemodialysis patients. https://trialsearch.who.int/Trial2.aspx?TrialID=IRCT2012111211438N1, 2012 | added to CENTRAL: 31 March 2019 | 2019 Issue 3 ICTRP	No pregnancy
128	IRCT201202067290N1. Evaluation of atorvastatine effect in reduction of negative symptoms in inpatients with schizophrenia disorder. https://trialsearch.who.int/Trial2.aspx?TrialID=IRCT201202067290N1, 2012 | added to CENTRAL: 31 March 2019 | 2019 Issue 3 ICTRP	No pregnancy
129	IRCT2015092924262N1. Turmeric effect on treatment non-alcoholic fatty liver disease. https://trialsearch.who.int/Trial2.aspx?TrialID=IRCT2015092924262N1, 2015 | added to CENTRAL: 31 March 2019 | 2019 Issue 3 ICTRP.	No pregnancy
130	IRCT20100524004010N24. The effect of curcumin supplementation on Nonalcoholic Fatty Liver Disease. https://trialsearch.who.int/Trial2.aspx?TrialID=IRCT20100524004010N24, 2018 | added to CENTRAL: 31 March 2019 | 2019 Issue 3 ICTRP.	Unrelated
131	Cochrane Central Register of Controlled Trials. Assessing the effectiveness of treatment by aripiprazole and placebo for control of obsesion in patients with Bipolar mood disorder. IRCT2014092719295N1. https://trialsearch.who.int/Trial2.aspx?TrialID=IRCT2014092719295N1, 2017 | added to CENTRAL: 31 March 2019 | 2019 Issue 3	Protocol
132	Cochrane Central Register of Controlled Trials. A Multicenter, Open-Label, Flexible-Dose Extension Study of Lurasidone Adjunctive to Lithium or Divalproex in Subjects with Bipolar I Disorder. EUCTR2011-004789-14-BG. https://trialsearch.who.int/Trial2.aspx?TrialID=EUCTR2011-004789-14-BG, 2012 | added to CENTRAL: 31 March 2019 | 2019 Issue 3	No pregnancy
133	IRCT201407111772N15. Post laparascopy shoulder pain. https://trialsearch.who.int/Trial2.aspx?TrialID=IRCT201407111772N15, 2014 | added to CENTRAL: 31 March 2019 | 2019 Issue 3 ICTRP.	Unrelated
134	IRCT201010124917N1. Therapeutic effect of herbal formulation Hypericum perforatum with antidepressant drug. https://trialsearch.who.int/Trial2.aspx?TrialID=IRCT201010124917N1, 2011 | added to CENTRAL: 31 March 2019 | 2019 Issue 3 ICTRP	No pregnancy
135	IRCT20100524004010N23. Investigate the effects of synbiotic supplement containing Bacillus coagulans and inulin in the treatment of non-alcoholic fatty liver. https://trialsearch.who.int/Trial2.aspx?TrialID=IRCT20100524004010N23, 2018 | added to CENTRAL: 31 March 2019 | 2019 Issue 3 ICTRP.	Unrelated
136	IRCT201211239014N13. Effect of selenium therapy on primary autoimmune hypothyroidism: a randomized clinical trial. https://trialsearch.who.int/Trial2.aspx?TrialID=IRCT201211239014N13, 2012 | added to CENTRAL: 31 March 2019 | 2019 Issue 3 ICTRP	No pregnancy
137	Cochrane Central Register of Controlled Trials. Efficacy of melatonin as an adjunct in the treatment of acute mania. IRCT201705181556N98. https://trialsearch.who.int/Trial2.aspx?TrialID=IRCT201705181556N98, 2017 | added to CENTRAL: 31 March 2019 | 2019 Issue 3	No pregnancy
138	EUCTR2012-001209-26-DE. Efficacy of Tranylcypromine (TCP) in daily doses up to 60mg and the combination of lithium and antidepressants (Li.-Aug.) in the acute treatmet of therapy-resistant Depression. In this study the assignment to the treatment groups (TCP or Li.-Aug.) is made by chance. Physicians and participants know which treatment is applicated. If a planned interim-analysis reveals no superiority of TCP the study will be terminated previously. https://trialsearch.who.int/Trial2.aspx?TrialID=EUCTR2012-001209-26-DE, 2013 | added to CENTRAL: 31 March 2019 | 2019 Issue 3 ICTRP	Protocol
139	Cochrane Central Register of Controlled Trials. Study of omega-3 supplements effects on treatment of patients with bipolar disorder [Study of use and non- use effects omega-3 supplements on treatment of patients with bipolar disorder] By: IRCT2013112414333N12. Source: IRCT2013112414333N12; https://trialsearch.who.int/Trial2.aspx?TrialID=IRCT2013112414333N12, 2013 | added to CENTRAL: 31 March 2019 | 2019 Issue 3	No pregnancy
140	IRCT2016101710324N34. The effect of metoclopramide administration to mother on newborn's bilirubin and mother's prolactine. https://trialsearch.who.int/Trial2.aspx?TrialID=IRCT2016101710324N34, 2017 | added to CENTRAL: 31 March 2019 | 2019 Issue 3 ICTRP.	No lithium
141	IRCT201311231197N17. Assessing effect of L-carnitine supplementation on nutritional status, serum inflammatory and oxidative stress factors, matrix metalloproteinase enzymes and clinical symptoms of females with knee osteoarthritis. https://trialsearch.who.int/Trial2.aspx?TrialID=IRCT201311231197N17, 2013 | added to CENTRAL: 31 March 2019 | 2019 Issue 3 ICTRP	Unrelated
142	Cochrane Central Register of Controlled Trials. Combined Remifentanil and Propofol in electroconvulsive therapy of acute mania. IRCT138810082935N1. https://trialsearch.who.int/Trial2.aspx?TrialID=IRCT138810082935N1, 2010 | added to CENTRAL: 31 March 2019 | 2019 Issue 3	No pregnancy
143	Cochrane Central Register of Controlled Trials. Pioglitazone for bipolar mixed or depressive episode. IRCT201211211556N46. https://trialsearch.who.int/Trial2.aspx?TrialID=IRCT201211211556N46, 2012 | added to CENTRAL: 31 March 2019 | 2019 Issue 3	No pregnancy
144	IRCT2014031211071N2. Evaluation of ondansetrone effect in reduction of negative symptoms in inpatients with schizophrenia disorder. https://trialsearch.who.int/Trial2.aspx?TrialID=IRCT2014031211071N2, 2014 | added to CENTRAL: 31 March 2019 | 2019 Issue 3 ICTRP	Unrelated
145	Cochrane Central Register of Controlled Trials. Effect of Adding Aripiprazole to Li Therapy in Children & Adolescents with BPD. IRCT201108027202N1. https://trialsearch.who.int/Trial2.aspx?TrialID=IRCT201108027202N1, 2011 | added to CENTRAL: 31 March 2019 | 2019 Issue 3.	Protocol
146	IRCT201709279014N190. Comparison of the effect of intravenous ibuprofen, intravenous acetaminophen, and intravenous morphine sulfate on treatment of abdominal pain in patients with appendicitis. https://trialsearch.who.int/Trial2.aspx?TrialID=IRCT201709279014N190, 2017 | added to CENTRAL: 31 March 2019 | 2019 Issue 3 ICTRP	Unrelated
147	Cochrane Central Register of Controlled Trials. Negative symptom treatment in schizophrenia disorder. IRCT138902113843N1. https://trialsearch.who.int/Trial2.aspx?TrialID=IRCT138902113843N1, 2010 | added to CENTRAL: 31 March 2019 | 2019 Issue 3	No pregnancy
148	IRCT2015110924968N1. Effect of Isothermic lavage liquid on incidence of shivering. https://trialsearch.who.int/Trial2.aspx?TrialID=IRCT2015110924968N1, 2015 | added to CENTRAL: 31 March 2019 | 2019 Issue 3 ICTRP	No pregnancy
149	ACTRN12617000354381. A micronutrient intervention for pregnant women experiencing symptoms of depression and anxiety. https://trialsearch.who.int/Trial2.aspx?TrialID=ACTRN12617000354381, 2017 | added to CENTRAL: 31 March 2019 | 2019 Issue 3 ICTRP.	Protocol
150	PACTR201310000635418. Li in HAND RCT. https://trialsearch.who.int/Trial2.aspx?TrialID=PACTR201310000635418, 2013 | added to CENTRAL: 31 March 2019 | 2019 Issue 3 ICTRP.	No pregnancy
151	IRCT201410073930N36. A Double-blind randomized placebo-controlled of memantine added to bipolar mood disorder treatment in obsession symptoms in patients with OCD. https://trialsearch.who.int/Trial2.aspx?TrialID=IRCT201410073930N36, 2014 | added to CENTRAL: 31 March 2019 | 2019 Issue 3 ICTRP.	No pregnancy
152	IRCT201105156493N1. The effect of doxycycline and zinc sulfate in acne. https://trialsearch.who.int/Trial2.aspx?TrialID=IRCT201105156493N1, 2011 | added to CENTRAL: 31 March 2019 | 2019 Issue 3 ICTRP	Unrelated
153	IRCT2015070523079N1. Evaluating the effect of Withania Somnifera on obsession. https://trialsearch.who.int/Trial2.aspx?TrialID=IRCT2015070523079N1, 2015 | added to CENTRAL: 31 March 2019 | 2019 Issue 3 ICTRP.	Unrelated

154	Cochrane Central Register of Controlled Trials. Effect of rivastigmine versus placebo on manic episode in patients with bipolar disorder. IRCT201704289014N161; https://trialsearch.who.int/Trial2.aspx?TrialID=IRCT201704289014N161, 2017 | added to CENTRAL: 31 March 2019 | 2019 Issue 3	No pregnancy
155	EUCTR2008-007190-20-DE. An International, Multicenter, Double-blind, Randomized, Placebo-controlled, Phase IV Study of the Safety and Efficacy of Lithium versus Placebo as an add on to SEROQUEL XR™ (Quetiapine Fumarate) in Adult Patients with Acute Mania. https://trialsearch.who.int/Trial2.aspx?TrialID=EUCTR2008-007190-20-DE, 2009 | added to CENTRAL: 31 March 2019 | 2019 Issue 3 ICTRP.	No pregnancy
156	EUCTR2007-002379-16-IT. MULTICENTER TRIAL (SINGLE BLIND)VS ON THE EFFICACY OF LITHIUM SALTS +RILUZOLE VS PLACEBO+RILUZOLE IN PATIENTS AFFECTED BY AMYOTROPHIC LATERAL SCLEROSIS - EFFICACY OF LITHIUM IN ALS. https://trialsearch.who.int/Trial2.aspx?TrialID=EUCTR2007-002379-16-IT, 2007 | added to CENTRAL: 31 March 2019 | 2019 Issue 3 ICTRP.	No pregnancy
157	Editorial. Drug treatments for bipolar disorder: 1—Acute manic or depressive episodes. Drug Ther Bull. 2005;43(4):28-32. doi: 10.1136/dtb.2005.43428. PMID: 15849974.	Opinion
158	NCT01003639. Idiopathic Intracranial Hypertension Treatment Trial. https://clinicaltrials.gov/show/NCT01003639, 2009 | added to CENTRAL: 31 January 2020 | 2020 Issue 01. CT.gov.	No lithium
159	NCT02347501. Dipeptidyl Peptidase-4 Inhibition and Narrow-band Ultraviolet-B Light in Psoriasis (DINUP). https://clinicaltrials.gov/show/NCT02347501, 2015 | added to CENTRAL: 31 January 2020 | 2020 Issue 01 CT.gov.	Unrelated
160	NCT03516734. Iron-fortified Lentils to Improve Iron (Fe) Status in Bangladesh. https://clinicaltrials.gov/show/NCT03516734, 2018 | added to CENTRAL: 31 January 2020 | 2020 Issue 01 CT.gov.	Unrelated
161	IRCT20160917029843N13. The Effect of dietary education training using adult education theory on the sexual health. https://trialsearch.who.int/Trial2.aspx?TrialID=IRCT20160917029843N13, 2019 | added to CENTRAL: 29 February 2020 | 2020 Issue 02 ICTRP	Unrelated
162	IRCT20191211045692N1. Efficacy of flax seed oil in treating mild to moderate psoriasis vulgaris comparing with topical Bethamethasonel. https://trialsearch.who.int/Trial2.aspx?TrialID=IRCT20191211045692N1, 2020 | added to CENTRAL: 31 October 2020 | 2020 Issue 10 ICTRP	Unrelated
163	IRCT20100524004010N30. Evaluation of the effects of a low free sugar diet in patients with Nonalcoholic fatty liver disease. https://trialsearch.who.int/Trial2.aspx?TrialID=IRCT20100524004010N30, 2020 | added to CENTRAL: 31 October 2020 | 2020 Issue 10 ICTRP.	Unrelated
164	IRCT20100524004010N32. The effect of Wheat Germ consumption on anthropometric indices, lipid profiles, glycemic status, hepatic enzymes, hepatocyte apoptosis, inflammatory factors, total antioxidant capacity and liver fibrosis in non-alcoholic fatty liver patients. https://trialsearch.who.int/Trial2.aspx?TrialID=IRCT20100524004010N32, 2020 | added to CENTRAL: 30 November 2020 | 2020 Issue 11 ICTRP.	Unrelated
165	IRCT20100524004010N31. Evaluation of the effects of Fasting Mimicking Diet (FMD) on lipid profile, glycemic, inflammatory and histologic indices in Non-Alcoholic Fatty Liver (NAFLD). https://trialsearch.who.int/Trial2.aspx?TrialID=IRCT20100524004010N31, 2020 | added to CENTRAL: 30 November 2020 | 2020 Issue 11 ICTRP.	Unrelated
166	Mukunya D, Tumwine JK, Nankabirwa V, Odongkara B, Tongun JB, Arach AA, Tumuhamye J, Napyo A, Zalwango V, Achora V, Musaba MW, Ndeezi G, Tylleskär T. Neonatal hypothermia in Northern Uganda: a community-based cross-sectional study. BMJ Open. 2021;11(2):e041723. doi: 10.1136/bmjopen-2020-041723. PMID: 33574146; PMCID: PMC7880091. Trial registration number: ClinicalTrial.gov as NCT02605369.	Duplicate= 101PM
167	EUCTR2020-000579-19-NL. An international multi-center clinical trial to investigate the efficacy of multiple drug compounds in patients with Amyotrophic Lateral Sclrerosis (ALS). https://trialsearch.who.int/Trial2.aspx?TrialID=EUCTR2020-000579-19-NL, 2021 | added to CENTRAL: 31 July 2021 | 2021 Issue 07 ICTRP. Goeman Borgesiuslaan 77 3515 ET Utrecht Netherlands, Stichting TRICALS Foundation	No pregnancy
168	NCT05434624. QT Distance and P Dispersion in ECG in Patients Having Bronchoscopy in the ICU. https://clinicaltrials.gov/show/NCT05434624, 2022 | added to CENTRAL: 31 July 2022 | 2022 Issue 07 CT.gov. Abant Izzet Baysal University: Emine Ozsari.	Unrelated
169	Arnold LE, Jensen PS. Micronutrients as treatment and prevention: New findings from 2 RCTs (maddy and nutrimum) for ADHD, emotional dysregulation, and antenatal depression. Journal of the American Academy of Child and Adolescent Psychiatry, 2022;61(10):S282.	No pregnancy
170	ISRCTN15671139. An international multi-center clinical trial to investigate the efficacy of multiple drug compounds in patients with amyotrophic lateral sclerosis. https://trialsearch.who.int/Trial2.aspx?TrialID=ISRCTN15671139, 2022 | added to CENTRAL: 31 October 2022 | 2022 Issue 10 ICTRP	No pregnancy
171	Fesslova VM, Gaeta G, Cavoretto PI, Boveri S, Piazza L, Andronache AA, Saracino A, Carminati M, Giamberti A, Frigiola A. Long term follow-up of fetal cases with tricuspid valve anomalies. Cardiology in the Young, 2022, 32, S78-S79 | added to CENTRAL: 30 November 2022 | 2022 Issue 11 Embase	Unrelated
172	ISRCTN13060336. ADA-HF: a pilot study of the safety and efficacy of acetazolamide in patients admitted to hospital with heart failure. https://trialsearch.who.int/Trial2.aspx?TrialID=ISRCTN13060336, 2023 | added to CENTRAL: 28 February 2023 | 2023 Issue 2 ICTRP	No pregnancy
173	IRCT20100524004010N37. The effect of replacing lunch grains with quinoa in the diet in patients with NAFLD. https://trialsearch.who.int/Trial2.aspx?TrialID=IRCT20100524004010N37, 2023 | added to CENTRAL: 30 April 2023 | 2023 Issue 4 ICTRP	Unrelated
174	NCT05923476. Electroconvulsive Therapy and Concomitant Lithium in Depressive Disorder: a Pilot Study. https://clinicaltrials.gov/show/NCT05923476, 2023 | added to CENTRAL: 31 July 2023 | 2023 Issue 7 CT.gov	No pregnancy
175	McKnight RF, Budge K, Adida M, Stockton S, Goodwin GM, Geddes JR. Lithium toxicity profile: a systematic review and meta-analysis. Bipolar Disord 2011;13(s1):72 (Poster P136). Abstracts of the Ninth International Conference on Bipolar Disorder, 9-11 June, 2011, Pittsburgh, PA, USA.	Review
176	Cochrane Central Register of Controlled Trials. Comparison of topiramate and placebo in controlling the OCD symptoms of bipolar patients [Comparison of treatment of bipolar disorder (lithium + olanzapine + clonazepam) and placebo with a treatment regimen containing topiramate in controlling the OCD symptoms of bipolar patients] By: IRCT2012061310013N1. Source: Http://www.who.int/trialsearch/trial2.aspx? Trialid=irct2012061310013n1 2012.	Protocol
177	Ichim L, Berk M, Brook S. LAMOTROGINE COMPARED TO LITHIUM IN MANIA: INTERIM RESULTS OF A RANDOMISED DOUBLE BLIND TRIAL. Review Group(s): Handsearch register from the US Cochrane Center. Source: XXI Collegium Internationale Neuro-psychopharmacologicum, Glasgow, Scotland. 12th-16th July, 1998. 1998. Cochrane Study Design: RCT.	No pregnancy
178	Cohen LS, Friedman JM, Jefferson JW, Johnson EM, Weiner ML. A reevaluation of risk of in utero exposure to lithium. JAMA. 1994;271(2):146-50. Erratum in: JAMA 1994;271(19):1485. PMID: 8031346.	Duplicate= 10 PM
179	Schneider RK. Divalproex did not differ from placebo or lithium in preventing recurrent of new mood episodes in bipolar I disorder, remission phase. Evid Based Ment Health. 2001;4:9. ISSN: 1362-0347. Opinion; Refers to: Bowden CL, Calabrese JR, McElroy SL, Gyulai L, Wassef A, Petty F, Pope HG Jr, Chou JC, Keck PE Jr, Rhodes LJ, Swann AC, Hirschfeld RM, Wozniak PJ. A randomized, placebo-controlled 12-month trial of divalproex and lithium in treatment of outpatients with bipolar I disorder. Divalproex Maintenance Study Group. Arch Gen Psychiatry. 2000;57(5):481-9. doi: 10.1001/archpsyc.57.5.481. PMID: 10807488.	No pregnancy
180	Hamilton JD. Lithium reduced aggression and was safe in aggressive children and adolescents with conduct disorder admitted to hospital. Evid Based Ment Health. 2001;4:17. ISSN: 1362-0347. Opinion; Refers to: Malone RP, Delaney MA, Luebbert JF, Cater J, Campbell M. A double-blind placebo-controlled study of lithium in hospitalized aggressive children and adolescents with conduct disorder. Arch Gen Psychiatry. 2000;57(7):649-54. doi: 10.1001/archpsyc.57.7.649. PMID: 10891035.	No pregnancy
181	Bullock R. A depression relapse prevention programme improved adherence to medication and depressive symptoms but did not decrease relapses. Evid Based Ment Health. 2001;4(4):113. doi: 10.1136/ebmh.4.4.113. ISSN: 1362-0347. Opinion; Refers to: Katon W, Rutter C, Ludman EJ, Von Korff M, Lin E, Simon G, Bush T, Walker E, Unützer J. A randomized trial of relapse prevention of depression in primary care. Arch Gen Psychiatry. 2001;58(3):241-7. doi: 10.1001/archpsyc.58.3.241. PMID: 11231831.	No pregnancy
182	Beemster P, Groenen P, Steegers-Theunissen R. Involvement of inositol in reproduction. Nutr Rev. 2002;60(3):80-7. doi: 10.1301/00296640260042748. PMID: 11908744.	Review
183	Royal Australian and New Zealand College of Psychiatrists Clinical Practice Guidelines Team for Bipolar Disorder. Australian and New Zealand clinical practice guidelines for the treatment of bipolar disorder. Aust N Z J Psychiatry. 2004;38(5):280-305. doi: 10.1080/j.1440-1614.2004.01356.x. PMID: 15144505.	Review
184	Gentile S. More than half the women with a history of psychosis have a psychiatric episode in the first year after childbirth. Evid Based Ment Health. 2005;8(2):33. doi: 10.1136/ebmh.8.2.33. ISSN: 1362-0347 PMID: NLM15851796. Opinion. Refers to: Howard LM, Goss C, Leese M, Appleby L, Thornicroft G. The psychosocial outcome of pregnancy in women with psychotic disorders. Schizophr Res. 2004;71(1):49-60. doi: 10.1016/j.schres.2004.01.003. PMID: 15374572.	Lumping
185	Newport DJ, Viguera AC, Beach AJ, Ritchie JC, Cohen LS, Stowe ZN. Lithium placental passage and obstetrical outcome: implications for clinical management during late pregnancy. Am J Psychiatry. 2005;162(11):2162-70. doi: 10.1176/appi.ajp.162.11.2162. PMID: 16263858.	Duplicate= 28PM
186	Howard LM. Atypical antipsychotic use during the first trimester of pregnancy may not increase major malformations. Evid Based Ment Health. 2005;8(4):115. doi: 10.1136/ebmh.8.4.115. PMID: 16246891. Opinion. Refers to: McKenna K, Koren G, Tetelbaum M, Wilton L, Shakir S, Diav-Citrin O, Levinson A, Zipursky RB, Einarson A. Pregnancy outcome of women using atypical antipsychotic drugs: a prospective comparative study. J Clin Psychiatry. 2005;66(4):444-9; quiz 546. doi: 10.4088/jcp.v66n0406. PMID: 15816786.	**Included**
187	Muzina DJ. Divalproex and lithium are similarly cost effective for adults with bipolar disorder. Evid Based Ment Health. 2006;9(1):15. doi: 10.1136/ebmh.9.1.15. PMID: 16436555. Opinion. Refers to: Revicki DA, Hirschfeld RM, Ahearn EP, Weisler RH, Palmer C, Keck PE Jr. Effectiveness and medical costs of divalproex	No pregnancy

	versus lithium in the treatment of bipolar disorder: results of a naturalistic clinical trial. J Affect Disord. 2005;86(2-3):183-93. doi: 10.1016/j.jad.2005.01.002. PMID: 15935238.	
188	Hilty DM, Leamon MH, Lim RF, Kelly RH, Hales RE. Diagnosis and treatment of bipolar disorder in the primary care setting: a concise review. Primary Psychiatry. 2006;13(7):77-85.	Review
189	Ryan M, Slevin JT. Restless legs syndrome. Am J Health Syst Pharm. 2006;63(17):1599-612. doi: 10.2146/ajhp060031. PMID: 16914630.	Review
190	Ward S, Wisner KL. Collaborative management of women with bipolar disorder during pregnancy and postpartum: pharmacologic considerations. J Midwifery Womens Health. 2007;52(1):3-13. doi: 10.1016/j.jmwh.2006.09.002. PMID: 17207745.	Duplicate= 35PM
191	Chou JC-Y. Continuing aripiprazole after stabilisation of a manic or mixed episode of bipolar I disorder delays relapse. Evid Based Ment Health. 2007;10(1):13. doi: 10.1136/ebmh.10.1.13. PMID: 17255382.	Opinion
192	Çabuk D, Sayin A, Derinöz O, Biri A. Quetiapine use for the treatment of manic episode during pregnancy. Arch Womens Ment Health. 2007;10(5):235-6. doi: 10.1007/s00737-007-0196-6. Epub 2007 Aug 6.	Case
193	Kim HG, Kolpe M. Pharmacologic treatment of psychiatric conditions during and after pregnancy. Primary Psychiatry, 2007;14(10):46-53.	Review
194	Blake LD, Lucas DN, Aziz K, Castello-Cortes A, Robinson PN. Lithium toxicity and the parturient: case report and literature review. Int J Obstet Anesth. 2008;17(2):164-9. doi: 10.1016/j.ijoa.2007.09.014. Epub 2008 Mar 4. PMID: 18308554.	Duplicate= 39PM
195	Pearlstein T. Perinatal depression: treatment options and dilemmas. J Psychiatry Neurosci. 2008;33(4):302-18. PMID: 18592032; PMCID: PMC2440793.	Review
196	Einarson A, Einarson TR. Maternal use of antipsychotics in early pregnancy: little evidence of increased risk of congenital malformations. Evid Based Ment Health. 2009;12(1):29. doi: 10.1136/ebmh.12.1.29.	Opinion
197	Grandjean EM, Aubry JM. Lithium: updated human knowledge using an evidence-based approach: part III: clinical safety. CNS Drugs. 2009;23(5):397-418. doi: 10.2165/00023210-200923050-00004. PMID: 19453201.	Review
198	Howland RH. Prescribing psychotropic medications during pregnancy and lactation: principles and guidelines. J Psychosoc Nurs Ment Health Serv. 2009;47(5):19-23. doi: 10.3928/02793695-20090331-05. PMID: 19489510.	Duplicate= 44PM
199	Diav-Citrin O, Einarson A. Lithium exposure during pregnancy linked with cardiovascular defects. Brown University Psychopharmacology Update. 2010;21(5):1-6. ISSN: 1068-5308. Wrong reference: Editorial. Lithium exposure during pregnancy linked with cardiovascular defects. Brown University Psychopharmacology Update. 2014;25(8):1,6.	Opinion
200	Burt VK, Bernstein C, Rosenstein WS, Altshuler LL. Bipolar disorder and pregnancy: maintaining psychiatric stability in the real world of obstetric and psychiatric complications. Am J Psychiatry. 2010;167(8):892-7. doi: 10.1176/appi.ajp.2009.09081248. PMID: 20693466.	Duplicate= 46PM
201	Galbally M, Roberts M, Buist A; Perinatal Psychotropic Review Group. Mood stabilizers in pregnancy: a systematic review. Aust N Z J Psychiatry. 2010;44(11):967-77. doi: 10.3109/00048674.2010.506637. PMID: 21034180.	Duplicate= 48PM
202	Anderson G. The role of melatonin in post-partum psychosis and depression associated with bipolar disorder. J Perinat Med. 2010;38(6):585-7. doi: 10.1515/jpm.2010.085. Epub 2010 Aug 13. PMID: 20707614.	Review
203	Elhassan HA, Thomson A, John G. Minerva. Case report. BMJ. 2011;342(7812):d3850.	Unrelated
204	McKnight RF, Adida M, Budge K, Stockton S, Goodwin GM, Geddes JR. Lithium toxicity profile: a systematic review and meta-analysis. Lancet. 2012;379(9817):721-8. doi: 10.1016/S0140-6736(11)61516-X. Epub 2012 Jan 20. PMID: 22265699.	Duplicate= 54PM
205	Kim HG. A piece of my mind. Drowning in plain sight. JAMA. 2012;307(18):1923-4. doi: 10.1001/jama.2012.3445. PMID: 22570461.	Duplicate= 56PM
206	van der Lugt NM, van de Maat JS, van Kamp IL, Knoppert-van der Klein EA, Hovens JG, Walther FJ. Fetal, neonatal and developmental outcomes of lithium-exposed pregnancies. Early Hum Dev. 2012;88(6):375-8. doi: 10.1016/j.earlhumdev.2011.09.013. Epub 2011 Oct 14. PMID: 22000820.	Duplicate= 53PM
207	Bergink V, Kushner SA. Lithium during pregnancy. Am J Psychiatry. 2014;171(7):712-5. doi: 10.1176/appi.ajp.2014.14030409. PMID: 24980165.	Duplicate= 62PM
208	Diav-Citrin O, Einarson A. Lithium exposure during pregnancy linked with cardiovascular defects. Brown University Psychopharmacology Update. 2010;21(5):1-6. ISSN: 1068-5308. Wrong reference: Editorial. Lithium exposure during pregnancy linked with cardiovascular defects. Brown University Psychopharmacology Update. 2014;25(8):1,6.	Duplicate= 22C
209	Huhta JC, Linask K. When should we prescribe high-dose folic acid to prevent congenital heart defects? Curr Opin Cardiol. 2015;30(1):125-31. doi: 10.1097/HCO.0000000000000124. PMID: 25389654.	Duplicate= 64PM
210	Hatters Friedman S, Moller-Olsen C, Prakash C, North A. Atypical antipsychotic use and outcomes in an urban maternal mental health service. Int J Psychiatry Med. 2016;51(6):521-533. doi: 10.1177/0091217417696739. Epub 2017 Mar 6. PMID: 28629296.	Duplicate= 73PM
211	Leong C, Raymond C, Château D, Dahl M, Alessi-Severini S, Falk J, Bugden S, Katz A. Psychotropic Drug Use before, during, and after Pregnancy: A Population- Based Study in a Canadian Cohort (2001-2013). Can J Psychiatry. 2017;62(8):543-550. doi: 10.1177/0706743717711168. Epub 2017 May 25. PMID: 28545329; PMCID: PMC5546669.	Duplicate= 72PM
212	Haskey C, Galbally M. Mood stabilizers in pregnancy and child developmental outcomes: A systematic review. Aust N Z J Psychiatry. 2017;51(11):1087-1097. doi: 10.1177/0004867417726175. Epub 2017 Aug 20. PMID: 28825316.	Duplicate= 74PM
213	Scrandis DA. Bipolar disorder in pregnancy: A review of pregnancy outcomes. J Midwifery Womens Health. 2017;62(6):673-683. doi: 10.1111/jmwh.12645. Epub 2017 Oct 30. PMID: 29083536.	Duplicate= 75PM
214	Thomson M, Sharma V. Weighing the risks: the management of bipolar disorder during pregnancy. Curr Psychiatry Rep. 2018;20(3):20. doi: 10.1007/s11920-018-0882-2. PMID: 29549608.	Duplicate= 77PM
215	Forsberg L, Adler M, Römer Ek I, Ljungdahl M, Navér L, Gustafsson LL, Berglund G, Chotigasatien A, Hammar U, Böhm B, Wide K. Maternal mood disorders and lithium exposure in utero were not associated with poor cognitive development during childhood. Acta Paediatr. 2018;107(8):1379-1388. doi: 10.1111/apa.14152. Epub 2017 Dec 5. PMID: 29150869.	Duplicate= 76PM
216	Frayne J, Nguyen T, Mok T, Hauck Y, Liira H. Lithium exposure during pregnancy: outcomes for women who attended a specialist antenatal clinic. J Psychosom Obstet Gynaecol. 2018;39(3):211-219. doi: 10.1080/0167482X.2017.1337743. Epub 2017 Jun 15. PMID: 28617151.	**Included**
217	Editorial. Lithium use in first trimester carries risk, but analysis finds overall magnitude small. Brown University Psychopharmacology Update, 2018;29(10):1-6. ISSN: 1068-5308.	Opinion
218	Hardy LT, Reichenbacker OL. A practical guide to the use of psychotropic medications during pregnancy and lactation. Arch Psychiatr Nurs. 2019;33(3):254-266. doi: 10.1016/j.apnu.2019.04.001. Epub 2019 Apr 10. PMID: 31227078.	Review
219	Editorial. Mood stabilizer use in pregnancy not linked with selected adverse birth outcomes. Brown University Psychopharmacology Update, 2019;30(10):3-4. ISSN: 1068-5308.	Opinion
220	Hermann A, Gorun A, Benudis A. Lithium use and non-use for pregnant and postpartum women with bipolar disorder. Curr Psychiatry Rep. 2019;21(11):114. doi: 10.1007/s11920-019-1103-3. PMID: 31701245.	Duplicate= 89PM
221	Fornaro M, Maritan E, Ferranti R, Zaninotto L, Miola A, Anastasia A, Murru A, Solé E, Stubbs B, Carvalho AF, Serretti A, Vieta E, Fusar-Poli P, McGuire P, Young AH, Dazzan P, Vigod SN, Correll CU, Solmi M. Lithium exposure during pregnancy and the postpartum period: A systematic review and meta-analysis of safety and efficacy outcomes. Am J Psychiatry. 2020;177(1):76-92. doi: 10.1176/appi.ajp.2019.19030228. Epub 2019 Oct 18. PMID: 31623458.	Duplicate= 88PM
222	Editorial. Lithium exposure in pregnancy found generally safe; risk is higher early. Brown University Psychopharmacology Update, 2020;31(2):1-5. ISSN: 1068-5308.	Opinion
223	Denton LK, Creeley CE, Stavola B, Hall K, Foltz BD. An analysis of online pregnancy message boards: Mother-to-mother advice on medication use. Women Birth. 2020;33(1):e48-e58. doi: 10.1016/j.wombi.2018.12.003. Epub 2018 Dec 10. PMID: 30545755.	Unfocused
224	Betcher HK, Wisner KL. Psychotropic treatment during pregnancy: Research synthesis and clinical care principles. J Womens Health (Larchmt). 2020;29(3):310-318. doi: 10.1089/jwh.2019.7781. Epub 2019 Dec 3. PMID: 31800350; PMCID: PMC7207058.	Review
225	Payne JL. Common errors psychiatrists make when managing mood disorders in pregnant patients. Psychiatric Times. 2020;37(3):16-17. 2p. (Journal Article - case study, pictorial) ISSN: 0893-2905	Opinion
226	Editorial. Lithium in pregnancy. Drug Ther Bull. 2021;59(1):5. doi: 10.1136/dtb.2020.000055. Epub 2020 Aug 31. PMID: 32868385.	Opinion
227	Johnson KD, Miller LN, Pouliot JD, Martin PR. Retrospective analysis of oxcarbazepine in pregnant women with substance use disorders: Focus on safety. J Pharm Pract. 2021;34(1):28-34. doi: 10.1177/0897190019850700. Epub 2019 Jun 23. PMID: 31232158.	Unfocused
228	Johnson KD, Smith ST, Pouliot JD, Miller LN. Comparison of pharmacist- and provider-managed lithium in an inpatient medical center: A 6-month review. J Am Pharm Assoc (2003). 2021;61(5):e103-e107. doi: 10.1016/j.japh.2021.06.014. Epub 2021 Jun 17. PMID: 34187759.	No birth outcomes

229	Wang Z, Chan AYL, Coghill D, Ip P, Lau WCY, Simonoff E, Brauer R, Wei L, Wong ICK, Man KKC. Association between prenatal exposure to antipsychotics and attention-deficit/hyperactivity disorder, autism spectrum disorder, preterm birth, and small for gestational age. JAMA Intern Med. 2021;181(10):1332-1340. doi: 10.1001/jamainternmed.2021.4571. PMID: 34398171; PMCID: PMC8369381.	No lithium
230	Hastie R, Tong S, Hiscock R, Lindquist A, Lindström L, Wikström AK, Sundström-Poromaa I. Maternal lithium use and the risk of adverse pregnancy and neonatal outcomes: a Swedish population-based cohort study. BMC Med. 2021;19(1):291. doi: 10.1186/s12916-021-02170-7. PMID: 34856987; PMCID: PMC8641220.	Duplicate= 106PM
231	Hastie R, Tong S, Hiscock R, Lindquist A, Lindström L, Wikström AK, Sundström-Poromaa I. Maternal lithium use and the risk of adverse pregnancy and neonatal outcomes: a Swedish population-based cohort study. BMC Med. 2021;19(1):291. doi: 10.1186/s12916-021-02170-7. PMID: 34856987; PMCID: PMC8641220.	Duplicate= 106PM
232	Sagué-Vilavella M, Solé E, Pinzón-Espinosa J, Sandra-Hernández A, Roda E, Vieta E, Roca A. Obstetric outcomes regarding the use of lithium in pregnant women with bipolar disorders: a prospective cohort study. Arch Womens Ment Health. 2022;25(4):729-737. doi: 10.1007/s00737-022-01234-8. Epub 2022 May 6. PMID: 35522327.	Duplicate= 109PM
233	Cerimele JM, Johnson M, Blanchard BE, Russo J, Unützer J, Fortney JC. Bipolar disorder in primary care: Medication treatment by co-located psychiatrists versus primary care clinicians supported by psychiatrists. Gen Hosp Psychiatry. 2022;78:108-110. doi: 10.1016/j.genhosppsych.2022.08.001. Epub 2022 Aug 4. PMID: 35985201; PMCID: PMC10686540.	No pregnancy
234	Liew Z, Meng Q, Yan Q, Schullehner J, Hansen B, Kristiansen SM, Voutchkova DD, Olsen J, Ersbøll AK, Ketzel M, Raaschou-Nielsen O, Ritz BR. Association Between Estimated Geocoded Residential Maternal Exposure to Lithium in Drinking Water and Risk for Autism Spectrum Disorder in Offspring in Denmark. JAMA Pediatr. 2023;177(6):617-624. doi: 10.1001/jamapediatrics.2023.0346. PMID: 37010840; PMCID: PMC10071398.	Unfocused
235	Robertson LJ, Dawood H, Leong TD. Management of bipolar disorder in South Africa: National Department of Health essential medicine selection and treatment algorithms. South Afr J Pub Health. 2023;7(1):e530. https://doi.org/10.7196/SSH.2023.v7i1.530.	Unrelated
236	Cohen JM, Huybrechts KF, Patorno E, Desai RJ, Mogun H, Bateman BT, Hernández-Díaz S. Anticonvulsant mood stabilizer and lithium use and risk of adverse pregnancy outcomes. J Clin Psychiatry. 2019;80(4):18m12572. doi: 10.4088/JCP.18m12572. PMID: 31237992.	Duplicate= 86PM
237	Bellwald J. Handicap, marriage and pregnancy: Psychiatric and psychological perspectives. Vierteljahresschrift für Heilpädagogik und ihre Nachbargebiete. 1976;45(1):42-49. Publisher: Heilpädagogisches Institut der Universität Freiburg; [Journal Article], Database: APA PsycInfo	Review
238	Riley EP, Lochry EA, Shapiro NR. Lack of response inhibition in rats prenatally exposed to alcohol. Psychopharmacology (Berl). 1979 Mar 29;62(1):47-52. doi: 10.1007/BF00426034. PMID: 108747.	Animal
239	Peachey, John B.; Madden, J. Spencer; Wilkinson, D. Adrian; Sanchez-Craig, Martha; O'Brien, Charles P.; Childress, Anna Rose; Vaillant, George E.; Drummond, D. Colin; Falkowski, W.; et al; Therapeutic skills. In: The international handbook of addiction behaviour. Glass, Ilana Belle (Ed); London, UK: Tavistock/Routledge; 1991, pp. 207-273.	Review
240	Maxmen, Jerrold S.; Psychotropic drugs: Fast facts. New York: W W Norton & Co; 1991. xxii, 279 pp.	Review
241	Jacobson SJ, Jones K, Johnson K, Ceolin L, Kaur P, Sahn D, Donnenfeld AE, Rieder M, Santelli R, Smythe J, et al. Prospective multicentre study of pregnancy outcome after lithium exposure during first trimester. Lancet. 1992;339(8792):530-3. doi: 10.1016/0140-6736(92)90346-5. PMID: 1346886.	Duplicate= 6PM
242	Austin MP. Puerperal affective psychosis: is there a case for lithium prophylaxis? Br J Psychiatry. 1992;161:692-4. doi: 10.1192/bjp.161.5.692. PMID: 1422621.	Duplicate= 7PM
243	Gelenberg AJ, Hopkins HS. Report on efficacy of treatments for bipolar disorder. Psychopharmacol Bull. 1993;29(4):447-56. PMID: 8084977.	Review
244	Loebstein R, Koren G. Pregnancy outcome and neurodevelopment of children exposed in utero to psychoactive drugs: the Motherisk experience. J Psychiatry Neurosci. 1997;22(3):192-6. PMID: 9183118; PMCID: PMC1188852. Unique identifier: 1997-04644-005.	Duplicate= 13PM
245	Stoner SC, Sommi RW Jr, Marken PA, Anya I, Vaughn J. Clozapine use in two full-term pregnancies. J Clin Psychiatry. 1997;58(8):364-5. doi: 10.4088/jcp.v58n0806f. PMID: 9515978.	Case
246	Walker A, Rosenberg M, Balaban-Gil K. Neurodevelopmental and neurobehavioral sequelae of selected substances of abuse and psychiatric medications in utero. Child Adolesc Psychiatr Clin N Am. 1999;8(4):845-67. PMID: 10553207.	Duplicate= 16PM
247	Wisner KL. 'Safety of antidepressant medications during pregnancy': Reply to Sontheimer DL, Ables AZ. Safety of antidepressant medications during pregnancy. JAMA. 2000;283(9):1139. doi: 10.1001/jama.283.9.1139. PMID: 10703770.	Opinion
248	Ernst CL, Goldberg JF. The reproductive safety profile of mood stabilizers, atypical antipsychotics, and broad-spectrum psychotropics. J Clin Psychiatry. 2002;63 Suppl 4:42-55. PMID: 11913676.	Review
249	Baldessarini RJ, Tondo L, Hennen J, Viguera AC. Is lithium still worth using? An update of selected recent research. Harv Rev Psychiatry. 2002;10(2):59-75. PMID: 11897747.	Duplicate= 21PM
250	Berk M, McKenzie H, Dodd S. Trichotillomania: response to lithium in a person with comorbid bipolar disorder. Hum Psychopharmacol. 2003;18(7):576-7. doi: 10.1002/hup.525. PMID: 14533143.	Case
251	Revicki DA, Hirschfeld RM, Ahearn EP, Weisler RH, Palmer C, Keck PE Jr. Effectiveness and medical costs of divalproex versus lithium in the treatment of bipolar disorder: results of a naturalistic clinical trial. J Affect Disord. 2005;86(2-3):183-93. doi: 10.1016/j.jad.2005.01.002. PMID: 15935238.	Duplicate= 10C
252	Newport DJ, Viguera AC, Beach AJ, Ritchie JC, Cohen LS, Stowe ZN. Lithium placental passage and obstetrical outcome: implications for clinical management during late pregnancy. Am J Psychiatry. 2005;162(11):2162-70. doi: 10.1176/appi.ajp.162.11.2162. PMID: 16263858.	Duplicate= 28PM
253	Even C, Dorocant ES, Thuile J, Kalck-Stern M, Guelfi JD. Grossesse, allaitement et thymorégulateurs: eléments de décisions et règles pour la pratique [Pregnancy, breast feeding and mood stabilisers: review and recommendations for practice]. Encéphale. 2006;32(2 Pt 1):224-30. French. doi: 10.1016/s0013-7006(06)76148-6. PMID: 16910623.	Duplicate= 33PM
254	Gentile S. Prophylactic treatment of bipolar disorder in pregnancy and breastfeeding: focus on emerging mood stabilizers. Bipolar Disord. 2006;8(3):207-20. doi: 10.1111/j.1399-5618.2006.00295.x. PMID: 16696822.	Duplicate= 31PM
255	Bauer MS, McBride L, Williford WO, Glick H, Kinosian B, Altshuler L, Beresford T, Kilbourne AM, Sajatovic M; Cooperative Studies Program 430 Study Team. Collaborative care for bipolar disorder: part I. Intervention and implementation in a randomized effectiveness trial. Psychiatr Serv. 2006;57(7):927-36. doi: 10.1176/ps.2006.57.7.927. PMID: 16816276.	Duplicate= 32 PM
256	Ward S, Wisner KL. Collaborative management of women with bipolar disorder during pregnancy and postpartum: pharmacologic considerations. J Midwifery Womens Health. 2007;52(1):3-13. doi: 10.1016/j.jmwh.2006.09.002. PMID: 17207745.	Duplicate= 35 PM
257	Olfson M, Marcus S. Neonatal risks of maternal treatment with mood stabilizers. Arch Gen Psychiatry. 2007;64(7):866-7; author reply 867-8. doi: 10.1001/archpsyc.64.7.866-b. PMID: 17606821. Comment on: Oberlander TF, Warburton W, Misri S, Aghajanian J, Hertzman C. Neonatal outcomes after prenatal exposure to selective serotonin reuptake inhibitor antidepressants and maternal depression using population-based linked health data. Arch Gen Psychiatry. 2006;63(8):898-906. doi: 10.1001/archpsyc.63.8.898. PMID: 16894066. No lithium	Opinion
258	Reis M, Källén B. Maternal use of antipsychotics in early pregnancy and delivery outcome. J Clin Psychopharmacol. 2008;28(3):279-88. doi: 10.1097/JCP.0b013e318172b8d5. PMID: 18480684.	Duplicate= 40PM
259	Grandjean EM, Aubry JM. Lithium: updated human knowledge using an evidence-based approach: part III: clinical safety. CNS Drugs. 2009;23(5):397-418. doi: 10.2165/00023210-200923050-00004. PMID: 19453201.	Duplicate= 20C
260	Howland RH. Prescribing psychotropic medications during pregnancy and lactation: principles and guidelines. J Psychosoc Nurs Ment Health Serv. 2009;47(5):19-23. doi: 10.3928/02793695-20090331-05. PMID: 19489510.	Duplicate= 44PM
261	Gentile S. Neurodevelopmental effects of prenatal exposure to psychotropic medications. Depress Anxiety. 2010;27(7):675-86. doi: 10.1002/da.20706. PMID: 20583298.	Duplicate= 45PM
262	Burt VK, Bernstein C, Rosenstein WS, Altshuler LL. Bipolar disorder and pregnancy: maintaining psychiatric stability in the real world of obstetric and psychiatric complications. Am J Psychiatry. 2010;167(8):892-7. doi: 10.1176/appi.ajp.2009.09081248. PMID: 20693466.	Duplicate= 46PM
263	Galbally M, Roberts M, Buist A; Perinatal Psychotropic Review Group. Mood stabilizers in pregnancy: a systematic review. Aust N Z J Psychiatry. 2010;44(11):967-77. doi: 10.3109/00048674.2010.506637. PMID: 21034180.	Duplicate= 48PM
264	Bergink V, Kushner SA, Pop V, Kuijpens H, Lambregtse-van den Berg MP, Drexhage RC, Wiersinga W, Nolen WA, Drexhage HA. Prevalence of autoimmune thyroid dysfunction in postpartum psychosis. Br J Psychiatry. 2011;198(4):264-8. doi: 10.1192/bjp.bp.110.082990. Epub 2011 Feb 22. PMID: 21343331.	Duplicate= 49PM
265	Hajek T, Alda M, Grof P. Discontinuation of lithium because of side effects. J Psychiatry Neurosci. 2011;36(6):E39-40. doi: 10.1503/jpn.110117. PMID: 22011562; PMCID: PMC3201996.	Case
266	Bergink V, Lambregtse-van den Berg MP, Koorengevel KM, Kupka R, Kushner SA. First-onset psychosis occurring in the postpartum period: a prospective cohort study. J Clin Psychiatry. 2011;72(11):1531-7. doi: 10.4088/JCP.10m06648. Epub 2011 Aug 23. PMID: 21903022.	Duplicate= 52PM

267	van der Lugt NM, van de Maat JS, van Kamp IL, Knoppert-van der Klein EA, Hovens JG, Walther FJ. Fetal, neonatal and developmental outcomes of lithium-exposed pregnancies. Early Hum Dev. 2012;88(6):375-8. doi: 10.1016/j.earlhumdev.2011.09.013. Epub 2011 Oct 14. PMID: 22000820.	Duplicate= 53PM
268	Bodén R, Lundgren M, Brandt L, Reutfors J, Andersen M, Kieler H. Risks of adverse pregnancy and birth outcomes in women treated or not treated with mood stabilisers for bipolar disorder: population based cohort study. BMJ. 2012;345:e7085. doi: 10.1136/bmj.e7085. PMID: 23137820; PMCID: PMC3493986.	Duplicate= 58PM
269	Bergink V, Kushner SA. Postpartum psychosis. In: Galbally M, Snellen M, Lewis A (Eds.); Psychopharmacology and Pregnancy: Treatment Efficacy, Risks, and Guidelines. New York: Springer-Verlag Publishing/Springer Nature; 2014, pp. 139-149.	Review
270	Costoloni G, Pierantozzi E, Goracci A, Bolognesi S, Fagiolini A. Leki normotymiczne a ciąża - przegląd [Mood stabilisers and pregnancy outcomes – a review]. Psychiatr Pol. 2014;48(5):865-87. Polish. doi: 10.12740/pp/25834. PMID: 25639010.	Duplicate= 66PM
271	Frayne J, Nguyen T, Kohan R, De Felice N, Rampono J. The comprehensive management of pregnant women with major mood disorders: a case study involving phenelzine, lithium, and quetiapine. Arch Womens Ment Health. 2014;17(1):73-5. doi: 10.1007/s00737-013-0386-3. Epub 2013 Nov 8. PMID: 24196828.	Duplicate= 59PM
272	Deligiannidis KM, Byatt N, Freeman MP. Pharmacotherapy for mood disorders in pregnancy: a review of pharmacokinetic changes and clinical recommendations for therapeutic drug monitoring. J Clin Psychopharmacol. 2014;34(2):244-55. doi: 10.1097/JCP.0000000000000087. PMID: 24525634; PMCID: PMC4105343.	Review
273	Adida M, McKnight RF, Budge K, Stockton S, Goodwin GM, Geddes JR. Profil toxique du lithium: Revue exhaustive de la littérature avec méta-analyse des résultats [Lithium toxicity profile: A systematic review and meta-analysis]. Ann Méd Psychol. 2014;172(3):212-218. DOI: 10.1016/j.amp.2014.03.001.	Review
274	Sutter-Dallay A-L. Le lithium et la grossesse [Lithium and pregnancy]. Ann Méd Psychol. 2014;172(3):229-233. DOI: 10.1016/j.amp.2014.02.015.	Review
275	Deiana V, Chillotti C, Manchia M, Carta P, Bocchetta A, Ardau R, Del Zompo M. Continuation versus discontinuation of lithium during pregnancy: a retrospective case series. J Clin Psychopharmacol. 2014;34(3):407-10. doi: 10.1097/JCP.0000000000000059. PMID: 24525653.	Duplicate= 60PM
276	Bergink V, Kushner SA. Lithium during pregnancy. Am J Psychiatry. 2014;171(7):712-5. doi: 10.1176/appi.ajp.2014.14030409. PMID: 24980165.	Duplicate= 62PM
277	Diav-Citrin O, Shechtman S, Tahover E, Finkel-Pekarsky V, Arnon J, Kennedy D, Erebara A, Einarson A, Ornoy A. Pregnancy outcome following in utero exposure to lithium: a prospective, comparative, observational study. Am J Psychiatry. 2014;171(7):785-94. doi: 10.1176/appi.ajp.2014.12111402. PMID: 24781368.	Duplicate= 61PM
278	Wesseloo R, Burgerhout KM, Koorengevel KM, Bergink V. Postpartumpsychose in de klinische praktijk: diagnostiek, behandeling en preventie [Postpartum psychosis in clinical practice: diagnostic considerations, treatment and prevention]. Tijdschr Psychiatr. 2015;57(1):25-33. Dutch. PMID: 25601625.	Duplicate= 65PM
279	Madden JM, Adams AS, LeCates RF, Ross-Degnan D, Zhang F, Huskamp HA, Gilden DM, Soumerai SB. Changes in drug coverage generosity and untreated serious mental illness: transitioning from Medicaid to Medicare Part D. JAMA Psychiatry. 2015;72(2):179-88. doi: 10.1001/jamapsychiatry.2014.1259. PMID: 25588123; PMCID: PMC4505620.	No pregnancy
280	Akman O, Moshé SL, Galanopoulou AS. Early life status epilepticus and stress have distinct and sex-specific effects on learning, subsequent seizure outcomes, including anticonvulsant response to phenobarbital. CNS Neurosci Ther. 2015;21(2):181-92. doi: 10.1111/cns.12335. Epub 2014 Oct 14. PMID: 25311088; PMCID: PMC6495315.	Animal
281	Larsen ER, Damkier P, Pedersen LH, Fenger-Gron J, Mikkelsen RL, Nielsen RE, Linde VJ, Knudsen HE, Skaarup L, Videbech P; Danish Psychiatric Society; Danish Society of Obstetrics and Gynecology; Danish Paediatric Society; Danish Society of Clinical Pharmacology. Use of psychotropic drugs during pregnancy and breast-feeding. Acta Psychiatr Scand. 2015;132(Suppl. 445):1-28. doi: 10.1111/acps.12479. PMID: 26344706.	Review
282	Rosso G, Albert U, Di Salvo G, Scatà M, Todros T, Maina G. Lithium prophylaxis during pregnancy and the postpartum period in women with lithium- responsive bipolar I disorder. Arch Womens Ment Health. 2016;19(2):429-32. doi: 10.1007/s00737-016-0601-0. Epub 2016 Jan 20. PMID: 26790685.	Duplicate= 67PM
283	Hatters Friedman S, Moller-Olsen C, Prakash C, North A. Atypical antipsychotic use and outcomes in an urban maternal mental health service. Int J Psychiatry Med. 2016;51(6):521-533. doi: 10.1177/0091217417696739. Epub 2017 Mar 6. PMID: 28629296.	Duplicate= 73PM
284	Leong C, Raymond C, Château D, Dahl M, Alessi-Severini S, Falk J, Bugden S, Katz A. Psychotropic Drug Use before, during, and after Pregnancy: A Population- Based Study in a Canadian Cohort (2001-2013). Can J Psychiatry. 2017;62(8):543-550. doi: 10.1177/0706743717711168. Epub 2017 May 25. PMID: 28545329; PMCID: PMC5546669.	Duplicate= 72PM
285	Scrandis DA. Bipolar disorder in pregnancy: A review of pregnancy outcomes. J Midwifery Womens Health. 2017;62(6):673-683. doi: 10.1111/jmwh.12645. Epub 2017 Oct 30. PMID: 29083536.	Duplicate= 75PM
286	Haskey C, Galbally M. Mood stabilizers in pregnancy and child developmental outcomes: A systematic review. Aust N Z J Psychiatry. 2017;51(11):1087-1097. doi: 10.1177/0004867417726175. Epub 2017 Aug 20. PMID: 28825316.	Duplicate= 74PM
287	Galbally M, Crabb C, Snellen M. Designing research that can untangle the effects in pregnancy of pharmacological treatments for mental disorders. Lancet Psychiatry. 2018;5(8):608-610. doi: 10.1016/S2215-0366(18)30214-1. Epub 2018 Jun 18. PMID: 29929875.	Opinion
288	Munk-Olsen T, Liu X, Viktorin A, Brown HK, Di Florio A, D'Onofrio BM, Gomes T, Howard LM, Khalifeh H, Krohn H, Larsson H, Lichtenstein P, Taylor CL, Van Kamp I, Wesseloo R, Meltzer-Brody S, Vigod SN, Bergink V. Maternal and infant outcomes associated with lithium use in pregnancy: an international collaborative meta-analysis of six cohort studies. Lancet Psychiatry. 2018;5(8):644-652. doi: 10.1016/S2215-0366(18)30180-9. Epub 2018 Jun 18. PMID: 29929874; PMCID: PMC6077091.	Duplicate= 79PM
289	Frayne J, Nguyen T, Mok T, Hauck Y, Liira H. Lithium exposure during pregnancy: outcomes for women who attended a specialist antenatal clinic. J Psychosom Obstet Gynaecol. 2018;39(3):211-219. doi: 10.1080/0167482X.2017.1337743. Epub 2017 Jun 15. PMID: 28617151.	Duplicate= 39C
290	Spinelli MG. Accurate Assessment of Risk of Major Malformations in Infants With First-Trimester Exposure to Quetiapine. Am J Psychiatry. 2018 Dec 1;175(12):1161-1162. doi: 10.1176/appi.ajp.2018.18070877. PMID: 30501412.	Opinion
291	Poels EMP, Bijma HH, Galbally M, Bergink V. Lithium during pregnancy and after delivery: a review. Int J Bipolar Disord. 2018;6(1):26. doi: 10.1186/s40345-018-0135-7. PMID: 30506447; PMCID: PMC6274637.	Duplicate= 83PM
292	Galbally M, Frayne J, Watson SJ, Snellen M. Psychopharmacological prescribing practices in pregnancy for women with severe mental illness: A multicentre study. Eur Neuropsychopharmacol. 2019;29(1):57-65. doi: 10.1016/j.euroneuro.2018.11.1103. Epub 2018 Nov 26. PMID: 30497841.	Duplicate= 82PM
293	Newmark RL, Bogen DL, Wisner KL, Isaac M, Ciolino JD, Clark CT. Risk-Benefit assessment of infant exposure to lithium through breast milk: a systematic review of the literature. Int Rev Psychiatry. 2019;31(3):295-304. doi: 10.1080/09540261.2019.1586657. Epub 2019 Jun 10. PMID: 31180257.	Duplicate= 85PM
294	Anmella G, Pacchiarotti I, Cubała WJ, Dudek D, Maina G, Thomas P, Vieta E. Expert advice on the management of valproate in women with bipolar disorder at childbearing age. Eur Neuropsychopharmacol. 2019;29(11):1199-1212. doi: 10.1016/j.euroneuro.2019.09.007. Epub 2019 Oct 4. PMID: 31590972.	Duplicate= 87PM
295	McLean K, Murphy KE, Dalfen A, Shea AK. The effect of maternal antidepressants on third trimester uteroplacental hemodynamics and the neonatal abstinence syndrome: a retrospective cohort study. Arch Womens Ment Health. 2019;22(6):791-797. doi: 10.1007/s00737-019-00954-8. Epub 2019 Feb 23. PMID: 30798375.	Duplicate= 84PM
296	Fornaro M, Maritan E, Ferranti R, Zaninotto L, Miola A, Anastasia A, Murru A, Solé E, Stubbs B, Carvalho AF, Serretti A, Vieta E, Fusar-Poli P, McGuire P, Young AH, Dazzan P, Vigod SN, Correll CU, Solmi M. Lithium exposure during pregnancy and the postpartum period: A systematic review and meta-analysis of safety and efficacy outcomes. Am J Psychiatry. 2020;177(1):76-92. doi: 10.1176/appi.ajp.2019.19030228. Epub 2019 Oct 18. PMID: 31623458.	Duplicate= 88PM
297	Solé E, Roca A, Torres A, Hernández AS, Fernández N, Díaz CN, Vieta E, Garcia-Esteve L. Obstetric complications in bipolar disorder: Psychiatric factors and the risk of caesarean section. Eur Neuropsychopharmacol. 2020;32:47-55. doi: 10.1016/j.euroneuro.2019.12.115. Epub 2020 Jan 3. PMID: 31911063.	Duplicate= 90PM
298	Betcher HK, Wisner KL. Psychotropic treatment during pregnancy: Research synthesis and clinical care principles. J Womens Health (Larchmt). 2020;29(3):310-318. doi: 10.1089/jwh.2019.7781. Epub 2019 Dec 3. PMID: 31800350; PMCID: PMC7207058.	Duplicate= 47C
299	Sharma V, Sharma P, Sharma S. Managing bipolar disorder during pregnancy and the postpartum period: a critical review of current practice. Expert Rev Neurother. 2020;20(4):373-383. doi: 10.1080/14737175.2020.1743684. Epub 2020 Mar 19. PMID: 32172610.	Duplicate= 93PM
300	Lebedevs T, Gan M, Teoh SWK, Brown P. Analysis of perinatal women attending a mother and baby unit taking sodium valproate or lithium with a diagnosis of bipolar affective disorder. Psychiatr Q. 2020;91(3):695-701. doi: 10.1007/s11126-020-09729-2. PMID: 32157548.	Duplicate= 92PM
301	Cuomo A, Amore M, Vampini C, Fagiolini A. Lamotrigina nel disturbo bipolare: prevenire la depressione per curare la malattia [Lamotrigine in bipolar disorder: preventing depression to treat the disease]. Riv Psichiatr. 2021;56(1):1-11. Italian. doi: 10.1708/3546.35215. PMID: 33560270.	Duplicate= 100PM
302	Molenaar NM, Poels EMP, Robakis T, Wesseloo R, Bergink V. Management of lithium dosing around delivery: An observational study. Bipolar Disord. 2021;23(1):49-54. doi: 10.1111/bdi.12955. Epub 2020 Jun 30. PMID: 32526071; PMCID: PMC7891390.	Duplicate= 96PM
303	Gannon JM. Three recommendations for addressing the ongoing lithium underutilization crisis in bipolar disorder. Bipolar Disord. 2021;23(1):84-85. doi: 10.1111/bdi.12914. Epub 2020 Apr 28. PMID: 32304620.	Opinion
304	Poels EMP, Schrijver L, White TJH, Roza SJ, Zarchev MG, Bijma H, Honig A, van Kamp IL, Hoogendijk WJG, Kamperman AM, Bergink V. The effect of prenatal lithium exposure on the neuropsychological development of the child. Bipolar Disord. 2022;24(3):310-319. doi: 10.1111/bdi.13133. Epub 2021 Oct 5. PMID: 34585812; PMCID: PMC9293321.	Duplicate= 105PM

305	Gilden J, Poels EMP, Lambrichts S, Vreeker A, Boks MPM, Ophoff RA, Kahn RS, Kamperman AM, Bergink V. Bipolar episodes after reproductive events in women with bipolar I disorder, a study of 919 pregnancies. J Affect Disord. 2021;295:72-79. doi: 10.1016/j.jad.2021.08.006. Epub 2021 Aug 8. PMID: 34416620.	Duplicate= 104PM
306	Kan ACO, Chan JKN, Wong CSM, Chen EYH, Chang WC. Psychotropic drug utilization patterns in pregnant women with bipolar disorder: A 16-year population-based cohort study. Eur Neuropsychopharmacol. 2022;57:75-85. doi: 10.1016/j.euroneuro.2022.01.115. Epub 2022 Feb 14. PMID: 35151952.	Duplicate= 108PM
307	Sagué-Vilavella M, Solé E, Pinzón-Espinosa J, Sandra-Hernández A, Roda E, Vieta E, Roca A. Obstetric outcomes regarding the use of lithium in pregnant women with bipolar disorders: a prospective cohort study. Arch Womens Ment Health. 2022;25(4):729-737. doi: 10.1007/s00737-022-01234-8. Epub 2022 May 6. PMID: 35522327.	Duplicate= 109PM
308	Whaites Heinonen E. Psychotropic drug treatment during pregnancy and lactation: Effects on mother and child. Månen, 9Q, Alfred Nobels allé 8, Karolinska Institutet, Flemingsberg, Dissertation, 20-5-2022. Dissertation Abstracts International: Section B: The Sciences and Engineering, 2022;84(2-B)	Duplicate= 115PM
309	Poels EMP, Kamperman AM, Bijma HH, Honig A, van Kamp IL, Kushner SA, Hoogendijk WJG, Bergink V, White T. Brain development after intrauterine exposure to lithium: A magnetic resonance imaging study in school-age children. Bipolar Disord. 2023;25(3):181-190. doi: 10.1111/bdi.13297. Epub 2023 Jan 20. PMID: 36633504.	Duplicate= 112PM
310	Schonewille NN, Terpstra PA, van den Heuvel MEN, Van Pampus MG, van den Heuvel OA, Broekman BFP. Neonatal admission after lithium use in pregnant women with bipolar disorders: a retrospective cohort study. Int J Bipolar Disord. 2023;11(1):24. doi: 10.1186/s40345-023-00306-7. PMID: 37450192; PMCID: PMC10348961.	Duplicate= 114PM
311	Whaites Heinonen E, Tötterman K, Bäck K, Sarman I, Forsberg L, Svedenkrans J. High lithium concentration at delivery is a potential risk factor for adverse outcomes in breastfed infants: a retrospective cohort study. Int J Bipolar Disord. 2023;11(1):36. doi: 10.1186/s40345-023-00317-4. PMID: 38032417; PMCID: PMC10689698.	Duplicate= 115PM
312	Schrijver L, Kamperman AM, Bijma H, van Kamp IL, Wesseloo R, Hoogendijk WJG, Bergink V, Poels EMP. Dose response relationship between lithium serum levels during pregnancy and birth outcomes. Acta Psychiatr Scand. 2024;149(4):323-331. doi: 10.1111/acps.13663. Epub 2024 Jan 18. PMID: 38238613.	Duplicate= 116PM
313	Galbally M, Snellen M, Walker S, Permezel M. Management of antipsychotic and mood stabilizer medication in pregnancy: recommendations for antenatal care. Aust N Z J Psychiatry. 2010;44(2):99-108. doi: 10.3109/00048670903487217. PMID: 20113298.	Review
314	Llewellyn A, Stowe ZN, Strader JR Jr. The use of lithium and management of women with bipolar disorder during pregnancy and lactation. J Clin Psychiatry. 1998;59(Suppl 6):57-64; discussion 65. PMID: 9674938.	Review
315	Hilty DM, Leamon MH, Lim RF, Kelly RH, Hales RE. A review of bipolar disorder in adults. Psychiatry (Edgmont). 2006;3(9):43-55. PMID: 20975827; PMCID: PMC2963467.	Review
316	Schou M. What happened later to the lithium babies? A follow-up study of children born without malformations. Acta Psychiatr Scand. 1976;54(3):193-7. doi: 10.1111/j.1600-0447.1976.tb00112.x. PMID: 970196.	**Included**
317	Menon SJ. Psychotropic medication during pregnancy and lactation. Arch Gynecol Obstet. 2008;277(1):1-13. doi: 10.1007/s00404-007-0433-2. Epub 2007 Aug 21. PMID: 17710428.	Review
318	BALANCE investigators and collaborators; Geddes JR, Goodwin GM, Rendell J, Azorin JM, Cipriani A, Ostacher MJ, Morriss R, Alder N, Juszczak E. Lithium plus valproate combination therapy versus monotherapy for relapse prevention in bipolar I disorder (BALANCE): a randomised open-label trial. Lancet. 2010;375(9712):385-95. doi: 10.1016/S0140-6736(09)61828-6. Epub 2010 Jan 19. PMID: 20092882.	No pregnancy
319	Bersudsky Y, Applebaum J, Gaiduk Y, Sharony L, Mishory A, Podberezsky A, Agam G, Belmaker RH. Valnoctamide as a valproate substitute with low teratogenic potential in mania: a double-blind, controlled, add-on clinical trial. Bipolar Disord. 2010;12(4):376-82. doi: 10.1111/j.1399-5618.2010.00828.x. PMID: 20636634.	Unrelated
320	Chiang CH, Sheu YH, Guo FR, Lin WW, Chen GR, Huang KC. Incorporating post-cessation weight-control coaching into smoking cessation therapy to reduce type 2 diabetes risk. Nutrients. 2021;13(10):3360. doi: 10.3390/nu13103360. PMID: 34684360; PMCID: PMC8539112.	Unrelated
321	Raz S, Gaffari SM, Fakhri A, Haghdoost MR, Ashrafi S. بررسی اثر افزودن سرترالین به آنتی سایکوتیک های تیپیک در کاهش علایم منفی بیماران اسکیزوفرنی بستری در بیمارستان گلستان اهواز در سال 1391 [Evaluation of typical antipsychotic augmentation with sertraline in reduction of negative symptoms in patients with schizophrenia in Ahvaz Golestan Hospital]. Jundishapur Scientific Medical Journal 2014;13(3-90):335-345.	No pregnancy
322	Rahmani S, Asgary S, Askari G, Keshvari M, Hatamipour M, Feizi A, Sahebkar A. Treatment of non-alcoholic fatty liver disease with curcumin: A randomized placebo-controlled trial. Phytother Res. 2016;30(9):1540-8. doi: 10.1002/ptr.5659. Epub 2016 Jun 8. PMID: 27270872.	No pregnancy
323	Saadati S, Sadeghi A, Mansour A, Yari Z, Poustchi H, Hedayati M, Hatami B, Hekmatdoost A. Curcumin and inflammation in non-alcoholic fatty liver disease: a randomized, placebo controlled clinical trial. BMC Gastroenterol. 2019;19(1):133. doi: 10.1186/s12876-019-1055-4. PMID: 31345163; PMCID: PMC6659284.	No pregnancy
324	Loebel A, Cucchiaro J, Silva R, Kroger H, Sarma K, Xu J, Calabrese JR. Lurasidone as adjunctive therapy with lithium or valproate for the treatment of bipolar I depression: a randomized, double-blind, placebo-controlled study. Am J Psychiatry. 2014;171(2):169-77. doi: 10.1176/appi.ajp.2013.13070985.	No pregnancy
325	Suppes T, Kroger H, Pikalov A, Loebel A. Lurasidone adjunctive with lithium or valproate for bipolar depression: A placebo-controlled trial utilizing prospective and retrospective enrolment cohorts. J Psychiatr Res. 2016;78:86-93. doi: 10.1016/j.jpsychires.2016.03.012. Epub 2016 Mar 31. PMID: 27089521.	No pregnancy
326	Movassaghi R, Peirovifar A, Aghamohammadi D, Mohammadipour Anvari H, Golzari SE, Kourehpaz Z. Premedication With Single Dose of Acetazolamide for the Control of Referral Shoulder Pain After Laparoscopic Cholecystectomy. Anesth Pain Med. 2015;5(6):e29366. doi: 10.5812/aapm.29366. PMID: 26705522; PMCID: PMC4688810.	Unrelated
327	Abhari K, Saadati S, Yari Z, Hosseini H, Hedayati M, Abhari S, Alavian SM, Hekmatdoost A. The effects of Bacillus coagulans supplementation in patients with non-alcoholic fatty liver disease: A randomized, placebo-controlled, clinical trial. Clin Nutr ESPEN. 2020 Oct;39:53-60. doi: 10.1016/j.clnesp.2020.06.020. Epub 2020 Jul 24. PMID: 32859329.	Unrelated
328	Moghaddam HS, Bahmani S, Bayanati S, Mahdavinasa M, Rezaei F, Akhondzadeh S. Efficacy of melatonin as an adjunct in the treatment of acute mania: a double-blind and placebo-controlled trial. Int Clin Psychopharmacol. 2020;35(2):81-88. doi: 10.1097/YIC.0000000000000298. PMID: 31743233.	No pregnancy
329	Shakeri J, Khanegi M, Golshani S, Farnia V, Tatari F, Alikhani M, Nooripour R, Ghezelbash MS. Effects of omega-3 supplement in the treatment of patients with bipolar I disorder. Int J Prev Med. 2016;7:77. doi: 10.4103/2008-7802.182734. PMID: 27280013; PMCID: PMC4882968.	No pregnancy
330	Tabrizi SO, Mirghafourvand M, Dost AJ, Mohammad-Alizadeh-Charandabi S, Javadzadeh Y, Seyedi R. Effect of metoclopramide administration to mothers on neonatal bilirubin and maternal prolactin: a randomized, controlled, clinical trial. World J Pediatr. 2019;15(2):135-142. doi: 10.1007/s12519-018-0217-8. Epub 2018 Dec 5. PMID: 30519818.	No lithium
331	Rezaei F, Nasseri K, Esfandiari GR, Sadeghi SM, Fathie M, Gharibi F. Remifentanil added to propofol for induction of anesthesia can reduce reorientation time after electroconvulsive therapy in patients with severe mania. J ECT. 2012;28(2):124-7. doi: 10.1097/YCT.0b013e31824d1cea. PMID: 22531206.	No pregnancy
332	Zeinoddini A, Sorayani M, Hassanzadeh E, Arbabi M, Farokhnia M, Salimi S, Ghaleiha A, Akhondzadeh S. Pioglitazone adjunctive therapy for depressive episode of bipolar disorder: a randomized, double-blind, placebo-controlled trial. Depress Anxiety. 2015;32(3):167-73. doi: 10.1002/da.22340. Epub 2015 Jan 23. PMID: 25620378.	No pregnancy
333	Aftab A, Kemp DE, Ganocy SJ, Schinagle M, Conroy C, Brownrigg B, D'Arcangelo N, Goto T, Woods N, Serrano MB, Han H, Calabrese JR, Gao K. Double-blind, placebo-controlled trial of pioglitazone for bipolar depression. J Affect Disord. 2019;245:957-964. doi: 10.1016/j.jad.2018.11.090. Epub 2018 Nov 13. PMID: 30699881.	No pregnancy
334	Nasab MN, Khajeddin N, Shanesaz AA, Pezeshki A. بررسی اثر افزودن سیتالوپرام به آنتی‌سایکوتیک‌های تیپیک در کاهش علایم منفی بیماران اسکیزوفرنی بستری در بیمارستان گلستان اهواز در سال 1389 [Evaluation of typical antipsychotic augmentation with citalopram in reduction of negative symptoms in inpatient schizophrenia in Ahvaz Golestan Hospital]. Jundishapur Scientific Medical Journal 2017;11(4-79):439-448.	No pregnancy
335	Bradley HA, Campbell SA, Mulder RT, Henderson JMT, Dixon L, Boden JM, Rucklidge JJ. Can broad-spectrum multinutrients treat symptoms of antenatal depression and anxiety and improve infant development? Study protocol of a double blind, randomized, controlled trial (the 'NUTRIMUM' trial). BMC Pregnancy Childbirth. 2020;20(1):488. doi: 10.1186/s12884-020-03143-z. PMID: 32842983; PMCID: PMC7448485.	Protocol
336	Anderson SG, McCaul M, Khoo S, Wiesner L, Sacktor N, Joska JA, Decloedt EH. The neurologic phenotype of South African patients with HIV-associated neurocognitive impairment. Neurol Clin Pract. 2020;10(1):15-22. doi: 10.1212/CPJ.0000000000000687. PMID: 32190416; PMCID: PMC7057065.	Unrelated
337	Lesosky M, Joska J, Decloedt E. Simulating therapeutic drug monitoring results for dose individualisation to maintain investigator blinding in a randomised controlled trial. Trials. 2017;18(1):261. doi: 10.1186/s13063-017-1992-6. PMID: 28592271; PMCID: PMC5463313.	No pregnancy
338	Decloedt EH, Lesosky M, Maartens G, Joska JA. Renal safety of lithium in HIV-infected patients established on tenofovir disoproxil fumarate containing antiretroviral therapy: analysis from a randomized placebo-controlled trial. AIDS Res Ther. 2017;14(1):6. doi: 10.1186/s12981-017-0134-2. PMID: 28160772; PMCID: PMC5292145.	No pregnancy

339	Sahraian A, Jahromi LR, Ghanizadeh A, Mowla A. Memantine as an adjuvant treatment for obsessive compulsive symptoms in manic phase of bipolar disorder: A randomized, double-blind, placebo-controlled clinical trial. J Clin Psychopharmacol. 2017;37(2):246-249. doi: 10.1097/JCP.0000000000000651. PMID: 28099183.	No pregnancy
340	Jahanbakhsh SP, Manteghi AA, Emami SA, Mahyari S, Gholampour B, Mohammadpour AH, Sahebkar A. Evaluation of the efficacy of Withania somnifera (Ashwagandha) root extract in patients with obsessive-compulsive disorder: A randomized double-blind placebo-controlled trial. Complement Ther Med. 2016;27:25-9. doi: 10.1016/j.ctim.2016.03.018. Epub 2016 Apr 9. PMID: 27515872.	Unrelated
341	Keshavrzi A, Rezaei H, Haghighi M, Jahangard L. Effect of Rivastigmine (Acetyl Cholinesterase Inhibitor) versus Placebo on Manic Episodes in Patients with Bipolar Disorders: Results from a Double Blind, Randomized, Placebo-Controlled Clinical Trial. Neuropsychobiology. 2019;78(4):200-208. doi: 10.1159/000501210. Epub 2019 Jul 17. PMID: 31315114.	No pregnancy
342	Bourin MS, Severus E, Schronen JP, Gass P, Szamosi J, Eriksson H, Chandrashekar H. Lithium as add-on to quetiapine XR in adult patients with acute mania: a 6-week, multicenter, double-blind, randomized, placebo-controlled study. Int J Bipolar Disord. 2014;2:14. doi: 10.1186/s40345-014-0014-9. PMID: 25505693; PMCID: PMC4224669.	No pregnancy
343	Aggarwal SP, Zinman L, Simpson E, McKinley J, Jackson KE, Pinto H, Kaufman P, Conwit RA, Schoenfeld D, Shefner J, Cudkowicz M; Northeast and Canadian Amyotrophic Lateral Sclerosis consortia. Safety and efficacy of lithium in combination with riluzole for treatment of amyotrophic lateral sclerosis: a randomised, double-blind, placebo-controlled trial. Lancet Neurol. 2010;9(5):481-8. doi: 10.1016/S1474-4422(10)70068-5. Epub 2010 Apr 1. PMID: 20363190; PMCID: PMC3071495.	No pregnancy
344	NORDIC Idiopathic Intracranial Hypertension Study Group Writing Committee; Wall M, McDermott MP, Kieburtz KD, Corbett JJ, Feldon SE, Friedman DI, Katz DM, Keltner JL, Schron EB, Kupersmith MJ. Effect of acetazolamide on visual function in patients with idiopathic intracranial hypertension and mild visual loss: the idiopathic intracranial hypertension treatment trial. JAMA. 2014;311(16):1641-51. doi: 10.1001/jama.2014.3312. PMID: 24756514; PMCID: PMC4362615.	No lithium
345	Lynch M, Ahern TB, Timoney I, Sweeney C, Kelly G, Hughes R, Tobin AM, O'Shea D, Kirby B. Dipeptidyl peptidase-4 inhibition and narrow-band ultraviolet-B light in psoriasis (DINUP): study protocol for a randomised controlled trial. Trials. 2016;17:29. doi: 10.1186/s13063-016-1157-z. PMID: 26767505; PMCID: PMC4714444.	Protocol
346	Yunus FM, Jalal C, Das A, Afsana K, Podder R, Vandenberg A, DellaValle DM. Consumption of Iron-Fortified Lentils Is Protective against Declining Iron Status among Adolescent Girls in Bangladesh: Evidence from a Community-Based Double-Blind, Cluster-Randomized Controlled Trial. J Nutr. 2024:S0022-3166(24)00153-6. doi: 10.1016/j.tjnut.2024.03.005. Epub ahead of print 2024 Mar 6. PMID: 38458577.	Unrelated
347	Yunus FM, Jalal C, Afsana K, Podder R, Vandenberg A, DellaValle DM. Iron-fortified lentils to improve iron (Fe) status among adolescent girls in Bangladesh - study protocol for a double-blind community-based randomized controlled trial. Trials. 2019;20(1):251. doi: 10.1186/s13063-019-3309-4. PMID: 31046819; PMCID: PMC6498512.	Protocol
348	Khodami B, Hatami B, Yari Z, Alavian SM, Sadeghi A, Varkaneh HK, Santos HO, Hekmatdoost A. Effects of a low free sugar diet on the management of nonalcoholic fatty liver disease: a randomized clinical trial. Eur J Clin Nutr. 2022;76(7):987-994. doi: 10.1038/s41430-022-01081-x. Epub 2022 Jan 20. PMID: 35058605.	Unrelated
349	Salehi-Sahlabadi A, Kord-Varkaneh H, Kocaadam-Bozkurt B, Seraj SS, Alavian SM, Hekmatdoost A. Wheat germ improves hepatic steatosis, hepatic enzymes, and metabolic and inflammatory parameters in patients with nonalcoholic fatty liver disease: A randomized, placebo-controlled, double-blind clinical trial. Phytother Res. 2022 ;36(11):4201-4209. doi: 10.1002/ptr.7553. Epub 2022 Jul 17. PMID: 35843540.	Unrelated
350	Kord Varkaneh H, Salehi Sahlabadi A, Găman MA, Rajabnia M, Sedanur Macit-Çelebi M, Santos HO, Hekmatdoost A. Effects of the 5:2 intermittent fasting diet on non-alcoholic fatty liver disease: A randomized controlled trial. Front Nutr. 2022 9:948655. doi: 10.3389/fnut.2022.948655. PMID: 35958257; PMCID: PMC9360602.	Unrelated
351	McKnight RF, Adida M, Budge K, Stockton S, Goodwin GM, Geddes JR. Lithium toxicity profile: a systematic review and meta-analysis. Lancet. 2012;379(9817):721-8. doi: 10.1016/S0140-6736(11)61516-X. Epub 2012 Jan 20. PMID: 22265699.	Duplicate= 54PM
352	Cohen LS, Friedman JM, Jefferson JW et al. A reevaluation of risk of utero exposure to lithium. JAMA. 1994;271:146-50; 212.	Duplicate= 10PM
353	Jacobson SJ, Jones K, Johnson K et al. Prospective multicentre study of pregnancy outcome after lithium exposure during first trimester. Lancet. 1992;339:530-3; 213.	Duplicate= 6PM
354	Schou M, Amdisen A. Lithium and the placenta (letter). Am J Obstet Gynecol. 1995;122:541;214.	Opinion
355	Koren G, Pastuszak A, Jacobson S et al. The safety of antidepressants in pregnancy. In: Koren G, ed. Maternal and fetal toxicology a clinician guide. New York: Marcel Dekker, 1994: 59–76	Review
356	Keck PE Jr, Calabrese JR, McQuade RD, Carson WH, Carlson BX, Rollin LM, Marcus RN, Sanchez R; Aripiprazole Study Group. A randomized, double-blind, placebo-controlled 26-week trial of aripiprazole in recently manic patients with bipolar I disorder. J Clin Psychiatry. 2006;67(4):626-37. doi: 10.4088/jcp.v67n0414. PMID: 16669728.	No pregnancy
357	Cohen LS, Góez-Mogollón L, Sosinsky AZ, Savella GM, Viguera AC, Chitayat D, Hernández-Díaz S, Freeman MP. Risk of major malformations in infants following first-trimester exposure to quetiapine. Am J Psychiatry. 2018;175(12):1225-1231. doi: 10.1176/appi.ajp.2018.18010098. Epub 2018 Aug 16. PMID: 30111186.	Unfocused
358	Patorno E, Huybrechts KF, Bateman BT, Cohen JM, Desai RJ, Mogun H, Cohen LS, Hernandez-Diaz S. lithium use in pregnancy and the risk of cardiac malformations. N Engl J Med. 2017;376(23):2245-2254. doi: 10.1056/NEJMoa1612222.	**Included**
359	Troyer WA, Pereira GR, Lannon RA, Belik J, Yoder MC. Association of maternal lithium exposure and premature delivery. J Perinatol. 1993;13(2):123-7. PMID: 8515304.	**Included**
360	NCT02490241. Lithium Therapy: Understanding Mothers, Metabolism and Mood. Completed. Bipolar Disorder, Lithium. Northwestern University, Crystal T Clark. Observational. 2015-05-26–2022-10-05. Published as Clark CT, Newmark RL, Wisner KL, Stika C, Avram MJ. Lithium Pharmacokinetics in the Perinatal Patient With Bipolar Disorder. J Clin Pharmacol. 2022;62(11):1385-1392. doi: 10.1002/jcph.2089. Epub 2022 Jul 13. PMID: 35620848; PMCID: PMC9796861.	Unfocused
361	NCT05648435. Lithium Clearance in Patients With High Risk of Acute Kidney Injury. Completed. Acute Kidney Injury. Diagnostic Test: Lithium measures. University Hospital of North Norway, Tromsø, Observational. 2022-12-05–2023-04-12	No pregnancy
362	NCT05982340. Endogenous Lithium Clearance in Acute Kidney Injury. Recruiting. Acute Kidney Injury Due to Sepsis, Proximal Tubule Function. Lithium Clearance. University Hospital of North Norway, Tromsø, Lars Marius Ytrebø. Observational. 2023-07-31…	No pregnancy
363	NCT00408681. Lithium Carbonate in Treating Patients With Acute Intestinal Graft-Versus-Host-Disease (GVHD) After Donor Stem Cell Transplant. Completed WITH RESULTS. Accelerated Phase Chronic Myelogenous Leukemia; Adult Acute Lymphoblastic Leukemia in Remission; Adult Acute Myeloid Leukemia in Remission; lithium carbonate; laboratory biomarker analysis. Fred Hutchinson Cancer Center, Paul Martin. Interventional. 2006-12-06– 2017-03-07	No pregnancy
364	NCT03056248. Lithium in Acute Kidney Injury. Unknown status. Acute Kidney Injury; Placebo oral capsule; Lithium Carbonate, Lifespan, Sairah Sharif, Interventional. 2017-02-13–2017-02-17	No pregnancy
365	NCT06226311. Environmental Pollutants in Pregnancy – IoMumNEXT. Not yet recruiting. Early Childhood, Neurodevelopment. Early Childhood, Anthropometry. Data collection; Diagnostic Test: Urinary. Universidade do Porto, Elisa Keating, Janete Santos. Observational. 2022-12-06…	Unfocused
366	NCT05307042. Decline in Renal Concentration Ability in Lithium Treated Patients. Unknown status. Lithium Toxicities, Bipolar Disorder, Concentration Ability Impaired. Diagnostic Test: Deamino Arginine Vasopressin (dDAVP). Radboud University Medical Center, Nijmegen, Gelderland, M.J. van der Aa, T. Nijenhuis,Radboud. Observational. 2022-03-23–2022-04-01	No pregnancy
367	NCT02967653. Atorvastatin for the Treatment of Lithium-Induced Nephrogenic Diabetes Insipidus. Completed. Lithium Use, Nephrogenic Diabetes Insipidus, Atorvastatin, Lady Davis Institute, Karl Looper. Interventional. 2016-11-15–2020-09-16	No pregnancy
368	NCT05044611. AMIloride for the Treatment of Nephrogenic Diabetes Insipidus for Patients With Bipolar Disorder Treated With Lithium. Recruiting. Bipolar Disorder, Anhydrous Amiloride Hydrochloride, Placebo. Hôpitaux de Paris. VRTOVSNIK François, FLAMANT Martin. Interventional. 2021-08-27…	No pregnancy
369	NCT05190744. PB to Treat Hereditary Nephrogenic Diabetes Insipidus, ADPKD Treated With Tolvaptan, and Severely Polyuric Patients With Previous Lithium Administration. Recruiting. Autosomal Dominant Polycystic Kidney Disease; Nephrogenic Diabetes Insipidus; Acquired Nephrogenic Diabetes Insipidus, Drug: PB. Mayo Clinic, Fouad T. Chebib. Cameron King, Trinity Hooks. Interventional. 2021-12-29…	No pregnancy
370	NCT05428605. In Vitro Immunomodulation in Membranous Nephropathy Relapses. Recruiting. Extramembranous Glomerulopathy. Centre Hospitalier Universitaire de Nice, Barbara SEITZ-POLSKI, Céline FERNANDEZ. Interventional. 2022-06-10…	Unrelated
371	NCT05557201. Cohort of Patients Presenting Unexplained Recurrent Miscarriages and Identification of Early Miscarriage Recidivism Factors. Not yet recruiting. Miscarriage, Biological: Lymphocyte immunophenotyping. Hôpitaux de Paris, Arsene MEKINIAN, Observational. 2022-09-08…	No lithium

372	NCT04767451. Levels of Selected Microelements in Premature Ovarian Insufficiency. Unknown status. Premature Ovarian Failure. Cengiz Gokcek Women's and Children's Hospital, Gaziantep, Ali Ovayolu, Observational. 2021-02-19–2021-08-31	Unrelated
373	NCT04956354. The Use of Wireless Sensors in Neonatal Intensive Care. Recruiting. Preterm Birth; Apnea of Newborn. McGill University Health Centre/Research Institute of the McGill University Health Centre, Guilherme M Sant'Anna, Wissam Shalish. 2021-06-11…	Unrelated
374	NCT06094920. Treatment Optimization for Patients With Type 2 Diabetes Using Empagliflozin and Finerenone in a Remote Clinical Trial. Not yet recruiting. Diabetes Mellitus Type 2 With Proteinuria. University Medical Center Groningen, Jelle Beernink, Hiddo Lambers Heerspink. 2023-10-11 …	Unrelated

Included: 28
Excluded: 346

Review	75
No pregnancy	66
Unrelated	39
Opinion	22
Unfocused	18
Case	13
No lithium	8
Protocol	8
No birth outcomes	5
Animal	5
Lumping	2
Duplicates	85

Supplementary figures

Figure S1. Association between lithium exposure (at any time) and cardiac anomalies in the foetuses of women with a psychiatric disorder: Forest plot.

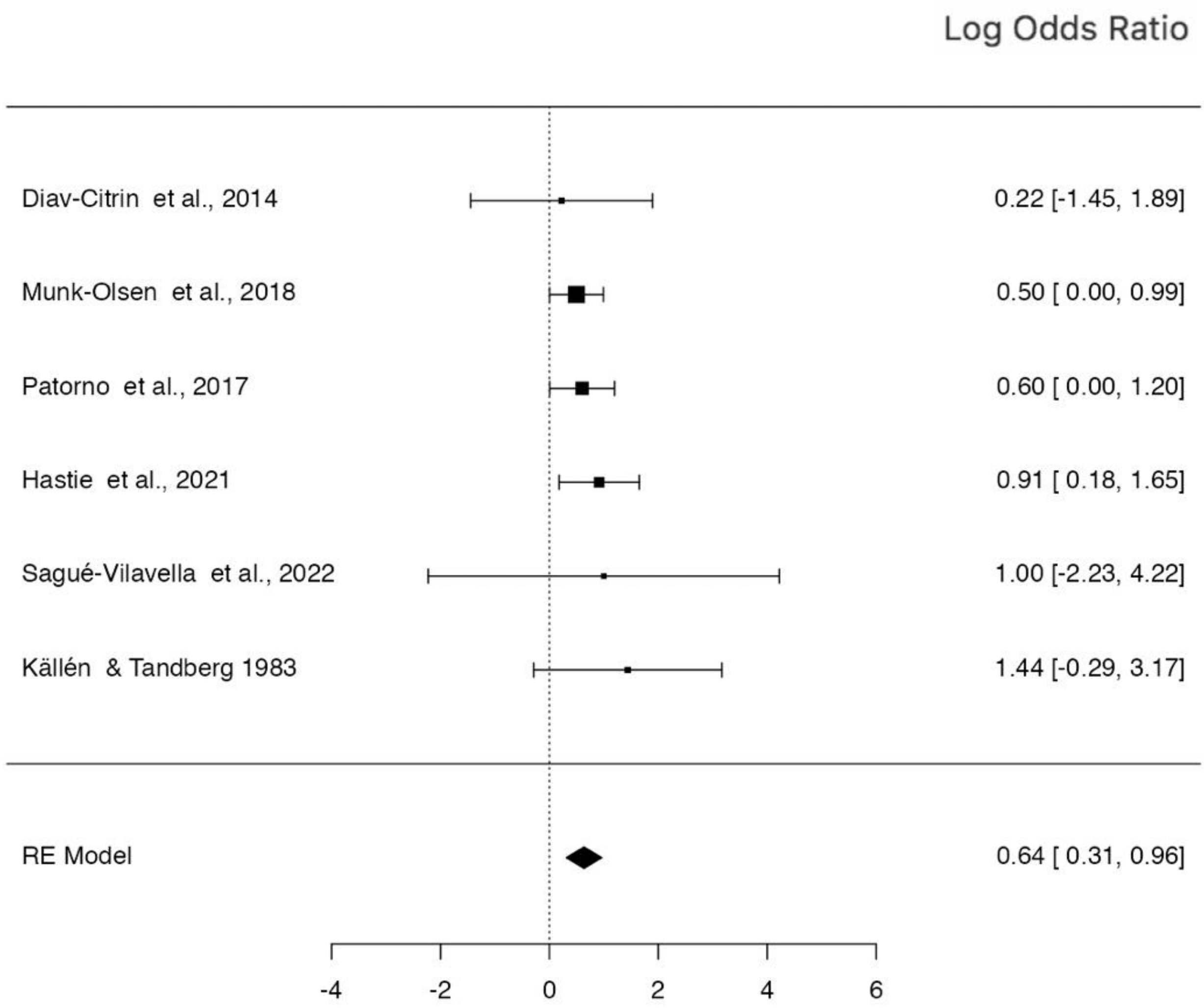

Figure S2. Association between lithium exposure (at any time) and any congenital anomaly in the foetuses of women with a psychiatric disorder: Forest plot.

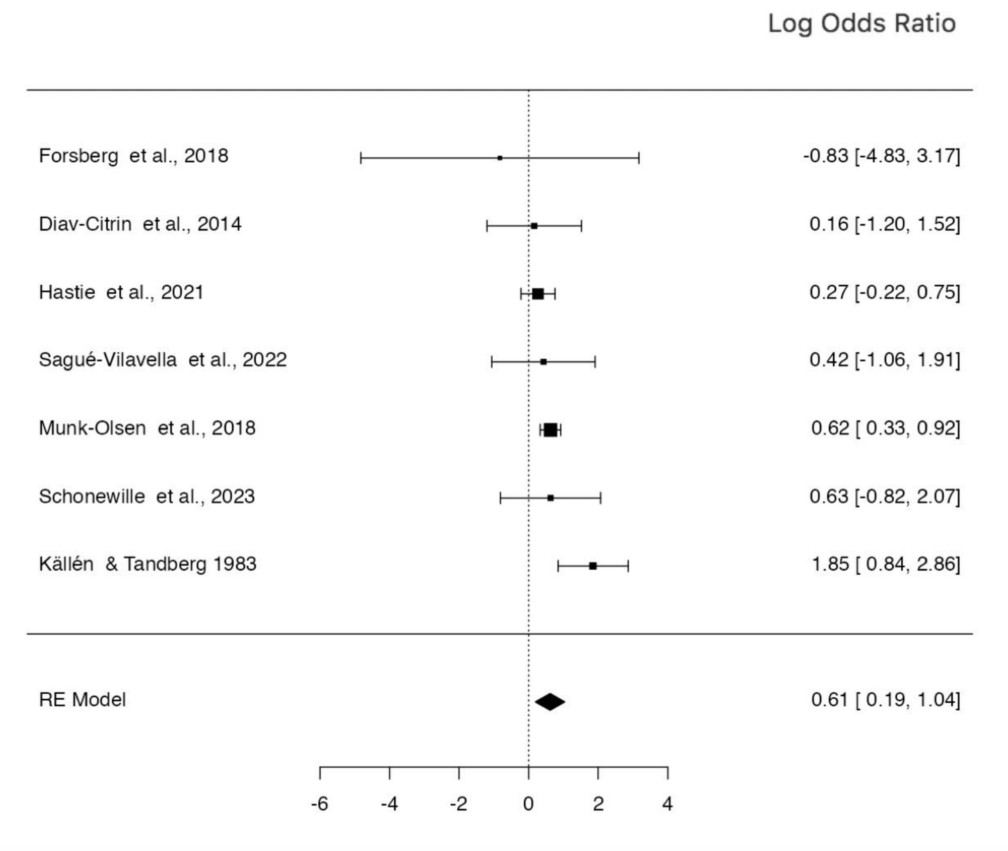

Figure S3. Association between lithium exposure (at any time) and preterm birth in women with a psychiatric disorder: Forest plot.

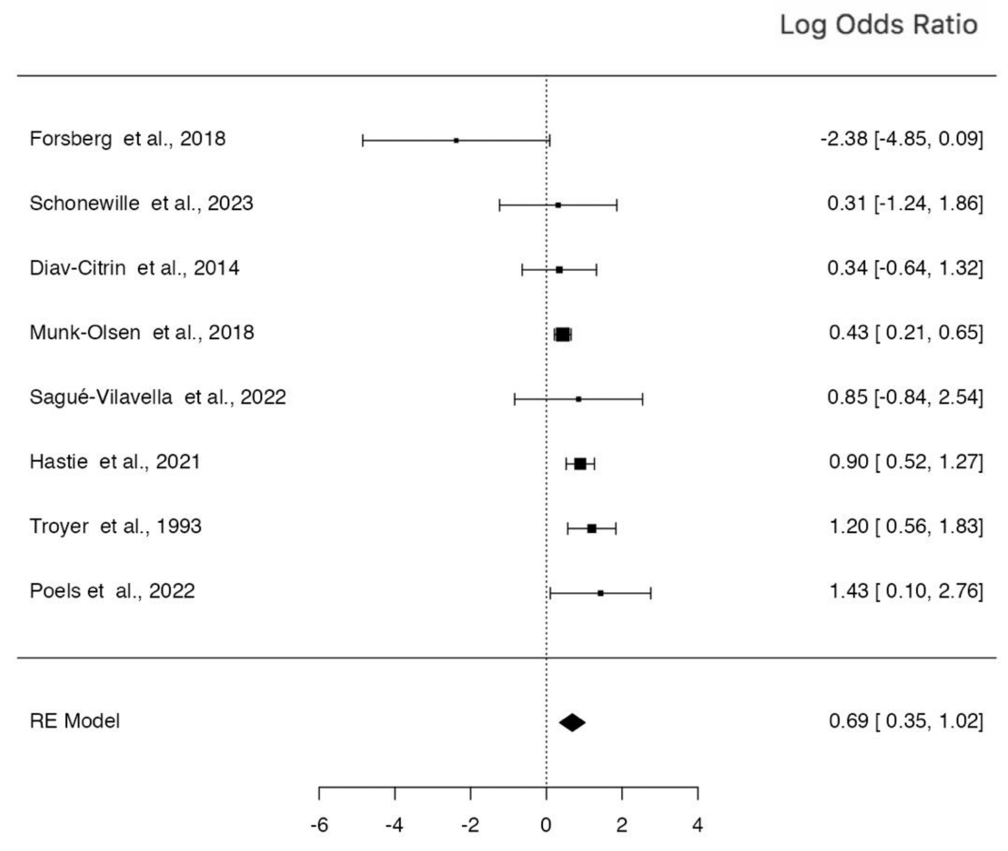

Figure S4. Association between lithium exposure (at any time) and child small for gestational age (SGA) in women with a psychiatric disorder: Forest plot.

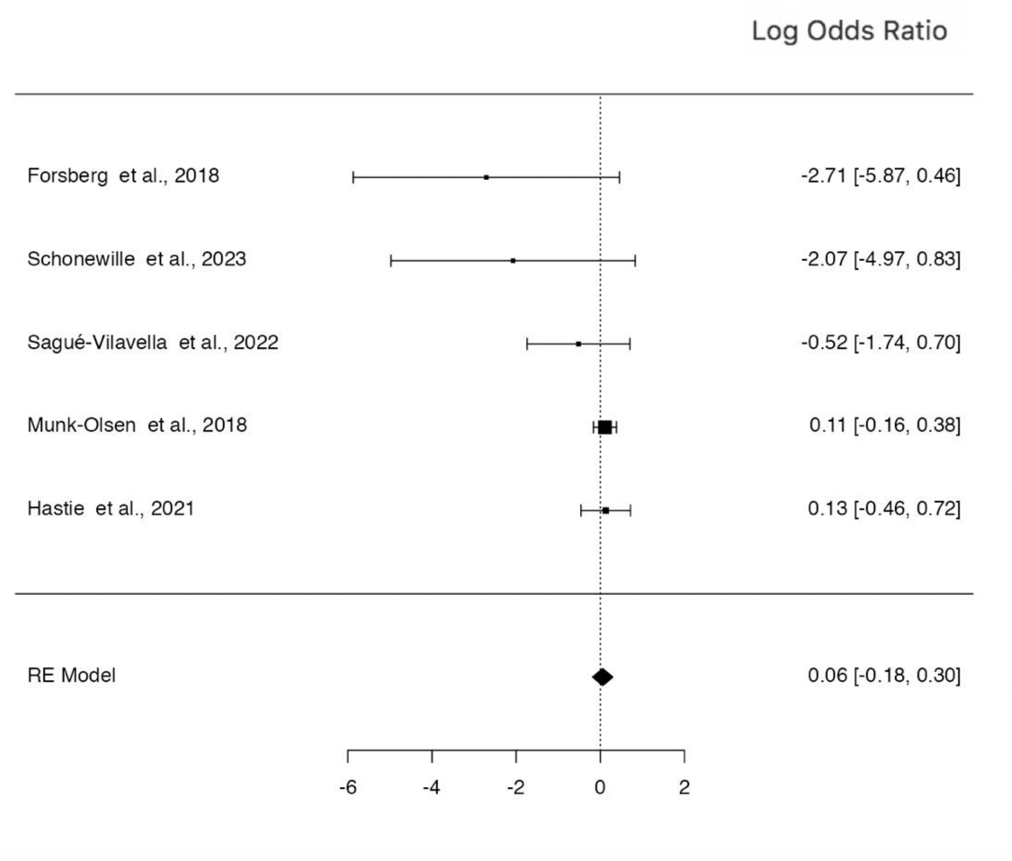

Figure S5. Association between lithium exposure (at any time) and child large for gestational age (LGA) in women with a psychiatric disorder: Forest plot.

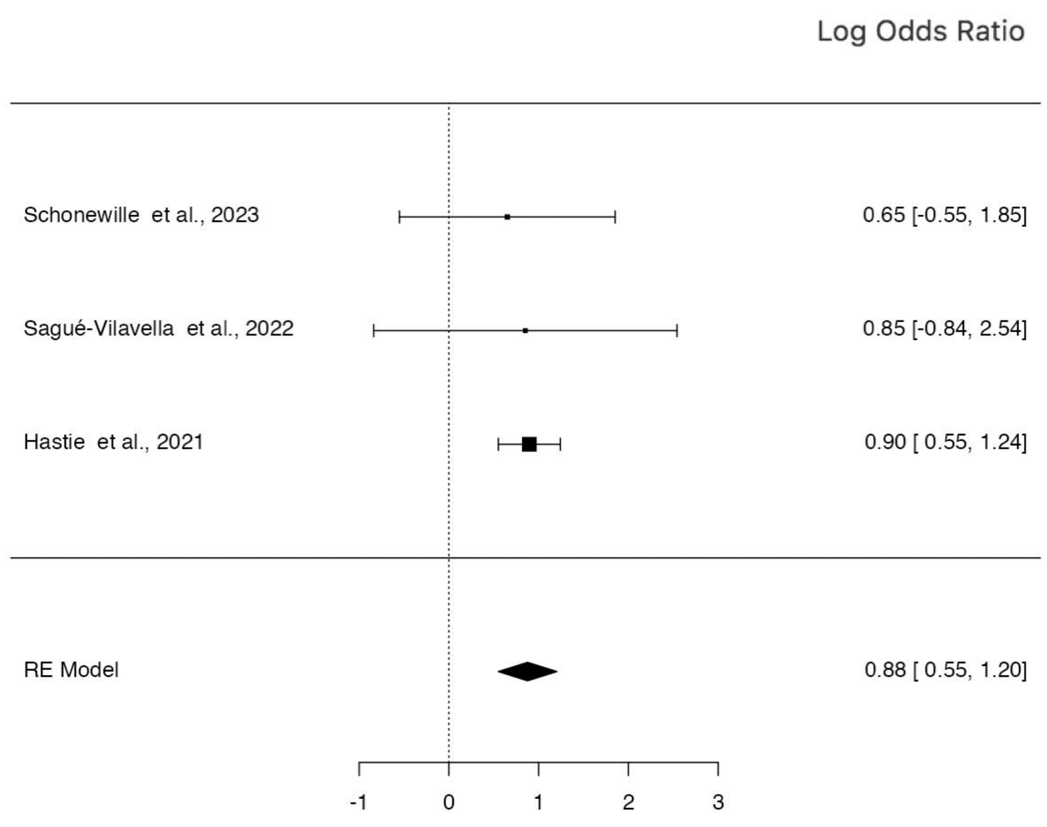

Figure S6. Association between lithium exposure (during the first trimester) and cardiac anomaly in the foetus of women with a psychiatric disorder: Forest plot.

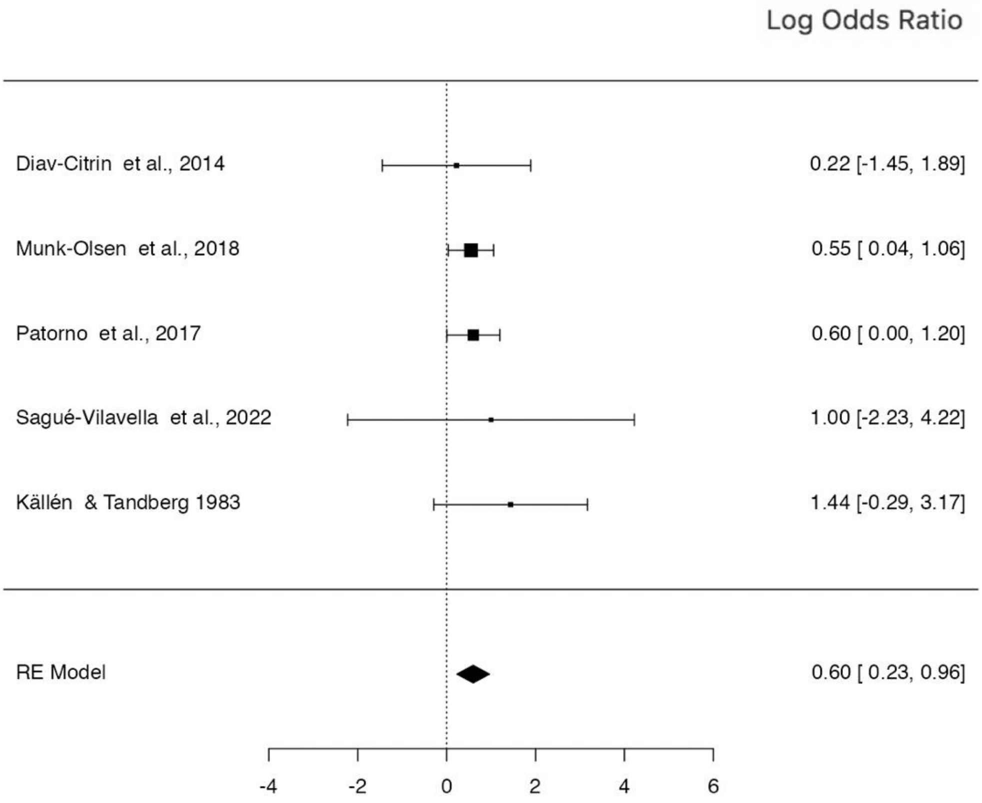

Figure S7. Association between lithium exposure (during the first trimester) and any congenital anomaly in the foetus of women with a psychiatric disorder: Forest plot.

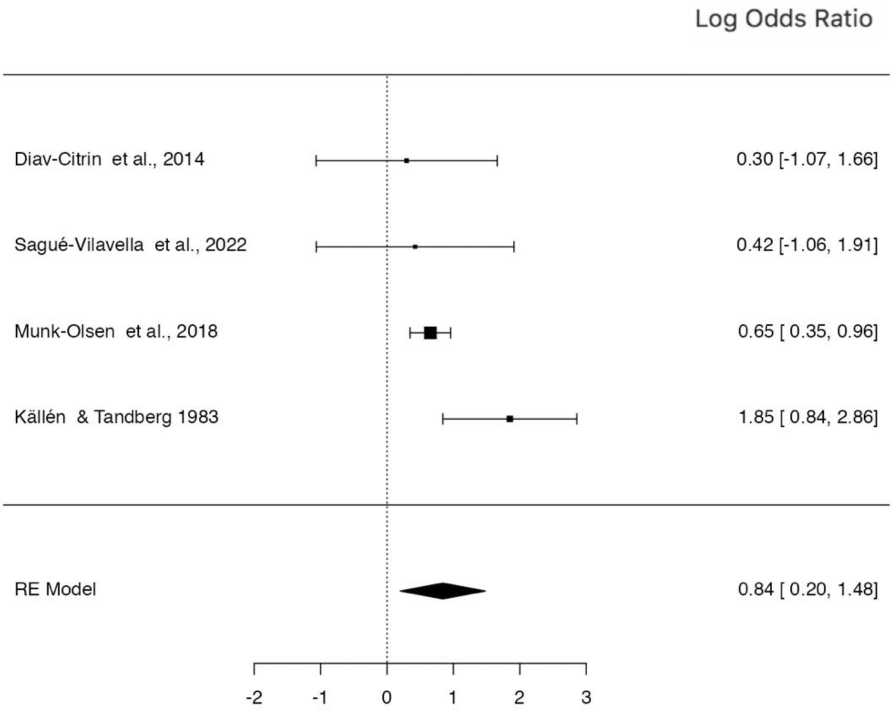

Supplementary Table S2. Summary of findings

Correlate	k	Lithium exposed women with psychiatric disorders	Lithium unexposed women with psychiatric disorders	Effect size (95%CI)	I^2
Lithium exposure at any time during pregnancy					
Cardiac anomaly	6	1776	39808	OR = 1.89 (1.37 to 2.61); ***p* < 0.001**	0%
Any congenital anomaly	7	1418	30406	OR = 1.84 (1.21 to 2.82); ***p* = 0.005**	40.2%
Preterm birth	8	1487	31054	OR = 1.99 (1.42 to 2.78); ***p* < 0.001**	42.9%
SGA	5	1218	30572	OR = 1.06 (0.83 to 1.35); *p* = 0.646	0%
LGA	3	507	9262	OR = 2.40 (1.73 to 3.33); ***p* < 0.001**	0%
Lithium exposure during the first trimester of pregnancy					
Cardiac anomaly	5	1292	30668	OR = 1.82 (1.26 to 2.62); ***p* = 0.001**	0%
Any congenital anomaly	4	856	21183	OR = 2.32 (1.22 to 4.40); ***p* = 0.010**	47.4%

95%CI = 95% confidence interval; k = number of studies with available information; N/A = not applicable; OR = odds ratio; *p* = p-value; SGA= small for gestational age; LGA= large for gestational age

References for the meta-analysis

[18] Källén, B.; Tandberg, A. Lithium and pregnancy. A cohort study on manic- depressive women. *Acta Psychiatr Scand* **1983**, *68*(2), 134-139. doi: 10.1111/j.1600-0447.1983.tb06991.x.

[19] Troyer, W.A.; Pereira, G.R.; Lannon, R.A.; Belik, J.; Yoder, M.C. Association of maternal lithium exposure and premature delivery. *J Perinatol* **1993**, *13*(2), 123-127.

[20] Diav-Citrin, O.; Shechtman, S.; Tahover, E.; Finkel-Pekarsky, V.; Arnon, J.; Kennedy, D.; Erebara, A.; Einarson, A.; Ornoy, A. Pregnancy outcome following in utero exposure to lithium: a prospective, comparative, observational study. *Am J Psychiatry* **2014**, *171*(7), 785-794. doi: 10.1176/appi.ajp.2014.12111402.

[21] Patorno, E.; Huybrechts, K.F.; Bateman, B.T.; Cohen, J.M.; Desai, R.J.; Mogun, H.; Cohen, L.S.; Hernandez-Diaz, S. Lithium use in pregnancy and the risk of cardiac malformations. *N Engl J Med* **2017**, *376*(23), 2245-2254. doi: 10.1056/NEJMoa1612222.

[22] Forsberg, L.; Adler, M.; Römer Ek, I.; Ljungdahl, M.; Navér, L.; Gustafsson, L.L.; Berglund, G.; Chotigasatien, A.; Hammar, U.; Böhm, B.; Wide, K. Maternal mood disorders and lithium exposure in utero were not associated with poor cognitive development during childhood. *Acta Paediatr* **2018**, *107*(8), 1379-1388. doi: 10.1111/apa.14152. Epub 2017 Dec 5.

[23] Munk-Olsen, T.; Liu, X.; Viktorin, A.; Brown, H.K.; Di Florio, A.; D'Onofrio, B.M.; Gomes, T.; Howard, L.M.; Khalifeh, H.; Krohn, H.; Larsson, H.; Lichtenstein, P.; Taylor, C.L.; Van Kamp, I.; Wesseloo, R.; Meltzer-Brody, S.; Vigod, S.N.; Bergink, V. Maternal and infant outcomes associated with lithium use in pregnancy: an international collaborative meta-analysis of six cohort studies. *Lancet Psychiatry* **2018**, *5*(8), 644-652. doi: 10.1016/S2215-0366(18)30180-9. Epub 2018 Jun 18.

[24] Hastie, R.; Tong, S.; Hiscock, R.; Lindquist, A.; Lindström, L.; Wikström, A.K.; Sundström-Poromaa, I. Maternal lithium use and the risk of adverse pregnancy and neonatal outcomes: a Swedish population-based cohort study. *BMC Med* **2021**, *19*(1), 291. doi: 10.1186/s12916-021-02170-7.

[25] Poels, E.M.P.; Schrijver, L.; White, T.J.H.; Roza, S.J.; Zarchev, M.G.; Bijma, H.; Honig, A.; van Kamp, I.L.; Hoogendijk, W.J.G.; Kamperman, A.M.; Bergink, V. The effect of prenatal lithium exposure on the neuropsychological development of the child. *Bipolar Disord* **2022**, *24*(3), 310-319. doi: 10.1111/bdi.13133. Epub 2021 Oct 5.

[26] Sagué-Vilavella, M.; Solé, E.; Pinzón-Espinosa, J.; Sandra-Hernández, A.; Roda, E.; Vieta, E.; Roca, A. Obstetric outcomes regarding the use of lithium in pregnant women with bipolar disorders: a prospective cohort study. *Arch Womens Ment Health* **2022**, *25*(4), 729-737. doi: 10.1007/s00737-022-01234-8. Epub 2022 May 6.

[27] Schonewille, N.N.; Terpstra, P.A.; van den Heuvel, M.E.N.; Van Pampus, M.G.; van den Heuvel, O.A.; Broekman, B.F.P. Neonatal admission after lithium use in pregnant women with bipolar disorders: a retrospective cohort study. *Int J Bipolar Disord* **2023**, 11(1), 24. doi: 10.1186/s40345-023-00306-7.

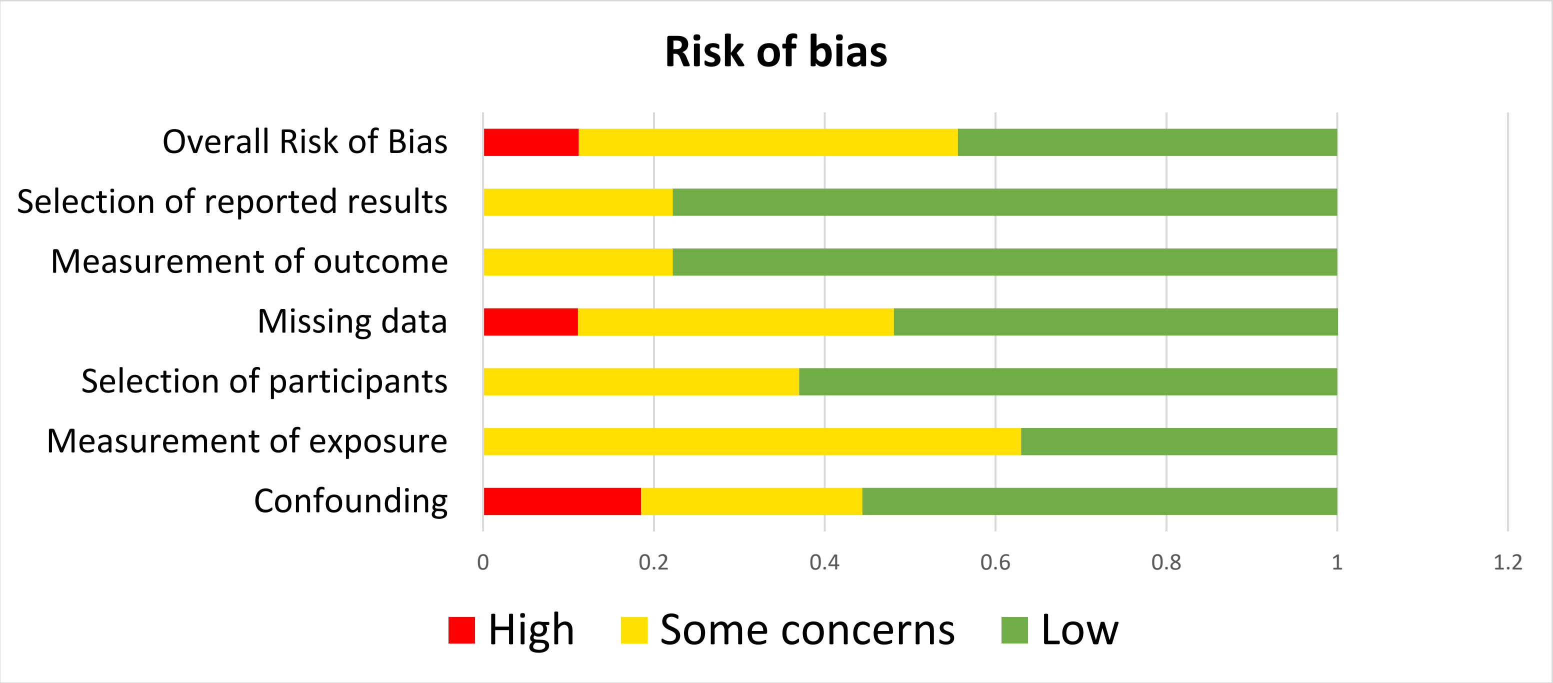

Author(s) Year	Confounding	Measurement of exposure	Selection of participants	Post-exposure intervention	Missing data	Measurement of outcome	Selection of reported results	OVERALL RISK OF BIAS
Schou, 1976	HIGH	SOME CONCERNS	SOME CONCERNS	SOME CONCERNS	HIGH	SOME CONCERNS	SOME CONCERNS	HIGH
Källén & Tandberg, 1983	HIGH	SOME CONCERNS	SOME CONCERNS	SOME CONCERNS	HIGH	SOME CONCERNS	SOME CONCERNS	HIGH
Jacobson et al., 1992	SOME CONCERNS	SOME CONCERNS	LOW	SOME CONCERNS	SOME CONCERNS	LOW	LOW	SOME CONCERNS
Troyer et al., 1993	HIGH	SOME CONCERNS	LOW	SOME CONCERNS	HIGH	LOW	SOME CONCERNS	HIGH
McKenna et al., 2005	HIGH	SOME CONCERNS	LOW	SOME CONCERNS	LOW	SOME CONCERNS	SOME CONCERNS	SOME CONCERNS
Reis & Källén, 2008	LOW	SOME CONCERNS	LOW	SOME CONCERNS	LOW	LOW	LOW	LOW
van der Lugt et al., 2011	SOME CONCERNS	SOME CONCERNS	LOW	SOME CONCERNS	SOME CONCERNS	LOW	LOW	SOME CONCERNS
Bodén et al., 2012	SOME CONCERNS	SOME CONCERNS	SOME CONCERNS	SOME CONCERNS	SOME CONCERNS	SOME CONCERNS	SOME CONCERNS	SOME CONCERNS
Frayne et al., 2013	HIGH	SOME CONCERNS	SOME CONCERNS	SOME CONCERNS	SOME CONCERNS	SOME CONCERNS	SOME CONCERNS	SOME CONCERNS
Diav-Citrin et al., 2014	LOW	SOME CONCERNS	SOME CONCERNS	SOME CONCERNS	SOME CONCERNS	LOW	LOW	SOME CONCERNS
Petersen et al., 2016	LOW	SOME CONCERNS	SOME CONCERNS	SOME CONCERNS	LOW	LOW	LOW	SOME CONCERNS
Rosso et al., 2016	SOME CONCERNS	LOW	LOW	SOME CONCERNS	SOME CONCERNS	LOW	LOW	LOW
Boyle et al., 2017	LOW	SOME CONCERNS	SOME CONCERNS	SOME CONCERNS	LOW	LOW	LOW	SOME CONCERNS
Patorno et al., 2017	LOW	LOW	SOME CONCERNS	LOW	LOW	LOW	LOW	LOW
Munk-Olsen et al., 2018	LOW	LOW	LOW	LOW	LOW	LOW	LOW	LOW
Forsberg et al., 2018	LOW	LOW	SOME CONCERNS	SOME CONCERNS	LOW	LOW	LOW	LOW
Neri et al., 2018	LOW	SOME CONCERNS	LOW	SOME CONCERNS	SOME CONCERNS	LOW	LOW	SOME CONCERNS
Poels et al., 2020	HIGH	SOME CONCERNS	SOME CONCERNS	SOME CONCERNS	HIGH	LOW	SOME CONCERNS	HIGH
Hastie et al., 2021	LOW	SOME CONCERNS	LOW	SOME CONCERNS	LOW	LOW	LOW	LOW
Molenaar et al., 2021	SOME CONCERNS	SOME CONCERNS	LOW	SOME CONCERNS	SOME CONCERNS	LOW	LOW	SOME CONCERNS
Poels et al., 2022	LOW	LOW	SOME CONCERNS	SOME CONCERNS	LOW	SOME CONCERNS	LOW	SOME CONCERNS
Sagué-Vilavella et al., 2022	LOW	LOW	LOW	SOME CONCERNS	LOW	LOW	LOW	LOW
Torfs et al., 2022	SOME CONCERNS	LOW	LOW	SOME CONCERNS	LOW	LOW	LOW	LOW
Álvarez-Silvares et al., 2023	LOW	LOW	LOW	SOME CONCERNS	SOME CONCERNS	LOW	LOW	LOW
Poels et al., 2023	LOW	LOW	LOW	SOME CONCERNS	LOW	LOW	LOW	LOW
Schonewille et al., 2023	LOW	SOME CONCERNS	LOW	SOME CONCERNS	LOW	LOW	LOW	LOW
Whaites Heinonen et al., 2023	SOME CONCERNS	LOW	LOW	SOME CONCERNS	SOME CONCERNS	LOW	LOW	SOME CONCERNS
Schrijver et al., 2024	LOW	SOME CONCERNS	LOW	SOME CONCERNS	LOW	LOW	LOW	LOW

Figure S8. Risk of Bias according to the Cochrane ROBINS-E tool (Higgins, J.P.T.; Morgan, R.L.; Rooney, A.A, Taylor, K.W.; Thayer, K.A.; Silva, R.A.; Lemeris, C.; Akl, E.A.; Bateson, T.F.; Berkman, N.D.; Glenn, B.S.; Hróbjartsson, A.; LaKind, J.S.; McAleenan, A.; Meerpohl, J.J.; Nachman, R.M.; Obbagy, J.E.; O'Connor, A.; Radke, E.G.; Savović, J.; Schünemann, H.J, Shea, B.; Tilling, K.; Verbeek, J.; Viswanathan, M.; Sterne, J.A.C. A tool to assess risk of bias in non-randomized follow-up studies of exposure effects (ROBINS-E). Environ Int 2024, 186, 108602. doi: 10.1016/j.envint.2024.108602. Epub 2024 Mar 24).

Note 1. “Measurement of exposure” has been considered at low risk of bias when the study reported on lithium blood levels, while some concerns are attributed to studies that only rely on the reported lithium prescription.

Note 2. “Selection of participants” has been considered at low risk of bias when the evaluation started close to the beginning of lithium exposure, whereas some concerns were attributed to studies in which the assessment was only retrospective through medical records.

Note 3. “Post-exposure intervention” has been considered with some concerns for all the studies because it is virtually impossible to assess all post-exposure variables and none of the studies had the chance to do so. Therefore, we decided to exclude this domain from the overall risk of bias assessment.

Based on the risk of bias assessment in the provided table, several insights can be drawn regarding the reliability and potential limitations of the studies evaluated.

The overall risk of bias varies significantly among the studies, with many marked as "High" due to multiple concerns across various domains of bias assessment. Notably, studies like Schou, 1976, Källén and Tandberg, 1983, and Frayne et al., 2018 exhibit high levels of bias primarily due to issues in confounding factors, selection of participants, and outcome measurements. These high-risk assessments suggest that the results from these studies should be interpreted with caution, as they might be influenced by factors that were not adequately controlled or accounted for during the study design or analysis.

On the other hand, studies like Álvarez et al., 2023, Poels et al., 2022, and Wei et al., 2022 show a "Low" overall risk of bias, indicating robust study designs and reliable methodologies that mitigate potential biases. These studies generally exhibit low concerns across most categories and, doing so, such studies provide more credible evidence and contribute more robustly to the body of knowledge in their respective fields.

Overall, this bias assessment highlights the varied quality of research studies and underscores the importance of critical evaluation of research methodologies to discern the reliability of study findings.

Section and Topic	Item #	Checklist item	Location where item is reported
**TITLE**			1
Title	1	Identify the report as a systematic review.	1
**ABSTRACT**			
Abstract	2	See the PRISMA 2020 for Abstracts checklist.	2
**INTRODUCTION**			2-3
Rationale	3	Describe the rationale for the review in the context of existing knowledge.	2-3
Objectives	4	Provide an explicit statement of the objective(s) or question(s) the review addresses.	3
**METHODS**			3-4
Eligibility criteria	5	Specify the inclusion and exclusion criteria for the review and how studies were grouped for the syntheses.	3-4
Information sources	6	Specify all databases, registers, websites, organisations, reference lists and other sources searched or consulted to identify studies. Specify the date when each source was last searched or consulted.	3
Search strategy	7	Present the full search strategies for all databases, registers and websites, including any filters and limits used.	3
Selection process	8	Specify the methods used to decide whether a study met the inclusion criteria of the review, including how many reviewers screened each record and each report retrieved, whether they worked independently, and if applicable, details of automation tools used in the process.	3-4
Data collection process	9	Specify the methods used to collect data from reports, including how many reviewers collected data from each report, whether they worked independently, any processes for obtaining or confirming data from study investigators, and if applicable, details of automation tools used in the process.	3-4
Data items	10a	List and define all outcomes for which data were sought. Specify whether all results that were compatible with each outcome domain in each study were sought (e.g. for all measures, time points, analyses), and if not, the methods used to decide which results to collect.	3-4
	10b	List and define all other variables for which data were sought (e.g. participant and intervention characteristics, funding sources). Describe any assumptions made about any missing or unclear information.	3-4
Study risk of bias assessment	11	Specify the methods used to assess risk of bias in the included studies, including details of the tool(s) used, how many reviewers assessed each study and whether they worked independently, and if applicable, details of automation tools used in the process.	4
Effect measures	12	Specify for each outcome the effect measure(s) (e.g. risk ratio, mean difference) used in the synthesis or presentation of results.	3-4
Synthesis methods	13a	Describe the processes used to decide which studies were eligible for each synthesis (e.g. tabulating the study intervention characteristics and comparing against the planned groups for each synthesis (item #5)).	3-4
	13b	Describe any methods required to prepare the data for presentation or synthesis, such as handling of missing summary statistics, or data conversions.	3-4
	13c	Describe any methods used to tabulate or visually display results of individual studies and syntheses.	3-4
	13d	Describe any methods used to synthesize results and provide a rationale for the choice(s). If meta-analysis was performed, describe the model(s), method(s) to identify the presence and extent of statistical heterogeneity, and software package(s) used.	3-4
	13e	Describe any methods used to explore possible causes of heterogeneity among study results (e.g. subgroup analysis, meta-regression).	3-4
	13f	Describe any sensitivity analyses conducted to assess robustness of the synthesized results.	3-4
Reporting bias assessment	14	Describe any methods used to assess risk of bias due to missing results in a synthesis (arising from reporting biases).	4
Certainty assessment	15	Describe any methods used to assess certainty (or confidence) in the body of evidence for an outcome.	3-4
**RESULTS**			4-12
Study selection	16a	Describe the results of the search and selection process, from the number of records identified in the search to the number of studies included in the review,	4-11

Section and Topic	Item #	Checklist item	Location where item is reported
		ideally using a flow diagram.	
	16b	Cite studies that might appear to meet the inclusion criteria, but which were excluded, and explain why they were excluded.	11, Suppl.
Study characteristics	17	Cite each included study and present its characteristics.	4-10
Risk of bias in studies	18	Present assessments of risk of bias for each included study.	Suppl.
Results of individual studies	19	For all outcomes, present, for each study: (a) summary statistics for each group (where appropriate) and (b) an effect estimate and its precision (e.g. confidence/credible interval), ideally using structured tables or plots.	Suppl.
Results of syntheses	20a	For each synthesis, briefly summarise the characteristics and risk of bias among contributing studies.	Suppl.
	20b	Present results of all statistical syntheses conducted. If meta-analysis was done, present for each the summary estimate and its precision (e.g. confidence/credible interval) and measures of statistical heterogeneity. If comparing groups, describe the direction of the effect.	5-11, Suppl.
	20c	Present results of all investigations of possible causes of heterogeneity among study results.	NA
	20d	Present results of all sensitivity analyses conducted to assess the robustness of the synthesized results.	NA
Reporting biases	21	Present assessments of risk of bias due to missing results (arising from reporting biases) for each synthesis assessed.	Suppl.
Certainty of evidence	22	Present assessments of certainty (or confidence) in the body of evidence for each outcome assessed.	Suppl.
**DISCUSSION**			12-15
Discussion	23a	Provide a general interpretation of the results in the context of other evidence.	12-15
	23b	Discuss any limitations of the evidence included in the review.	15
	23c	Discuss any limitations of the review processes used.	15
	23d	Discuss implications of the results for practice, policy, and future research.	12-15
**OTHER INFORMATION**			15-16, Suppl.
Registration and protocol	24a	Provide registration information for the review, including register name and registration number, or state that the review was not registered.	NA
	24b	Indicate where the review protocol can be accessed, or state that a protocol was not prepared.	15
	24c	Describe and explain any amendments to information provided at registration or in the protocol.	NA
Support	25	Describe sources of financial or non-financial support for the review, and the role of the funders or sponsors in the review.	16
Competing interests	26	Declare any competing interests of review authors.	16
Availability of data, code and other materials	27	Report which of the following are publicly available and where they can be found: template data collection forms; data extracted from included studies; data used for all analyses; analytic code; any other materials used in the review.	NA, Suppl.

From: Page MJ, McKenzie JE, Bossuyt PM, Boutron I, Hoffmann TC, Mulrow CD, et al. The PRISMA 2020 statement: an updated guideline for reporting systematic reviews. BMJ 2021;372:n71. doi: 10.1136/bmj.n71

For more information, visit: http://www.prisma-statement.org/
